# Machine learning insights into predicting biogas separation in metal-organic frameworks

**DOI:** 10.1038/s42004-024-01166-7

**Published:** 2024-05-08

**Authors:** Isabel Cooley, Samuel Boobier, Jonathan D. Hirst, Elena Besley

**Affiliations:** School of Chemistry, University of Nottingham, University Park, Nottingham, NG7 2RD UK

**Keywords:** Computational chemistry, Metal-organic frameworks, Porous materials

## Abstract

Breakthroughs in efficient use of biogas fuel depend on successful separation of carbon dioxide/methane streams and identification of appropriate separation materials. In this work, machine learning models are trained to predict biogas separation properties of metal-organic frameworks (MOFs). Training data are obtained using grand canonical Monte Carlo simulations of experimental MOFs which have been carefully curated to ensure data quality and structural viability. The models show excellent performance in predicting gas uptake and classifying MOFs according to the trade-off between gas uptake and selectivity, with *R*^2^ values consistently above 0.9 for the validation set. We make prospective predictions on an independent external set of hypothetical MOFs, and examine these predictions in comparison to the results of grand canonical Monte Carlo calculations. The best-performing trained models correctly filter out over 90% of low-performing unseen MOFs, illustrating their applicability to other MOF datasets.

## Abstract (web summary)

Breakthroughs in the efficient use of biogas fuel depend on the successful separation of carbon dioxide/methane streams and identification of appropriate separation materials. Here, machine learning models are trained to predict biogas separation properties of metal-organic frameworks.

## Introduction

Effective purification of the biogas stream obtained from decomposition of agricultural and industrial waste remains a challenging but promising goal towards a renewable source of biomethane fuel^1^ and a more sustainable alternative to fossil fuels^2^. Biogas is composed predominantly of a CH_4_/CO_2_ mixture which must be separated, along with trace contaminants, to obtain biomethane of increased purity for use in internal combustion engines^2^. Multiple established approaches^2–4^ are routinely used for upgrading the calorific content of biogas by removal of CO_2_, although these technologies can be costly and energy intensive. Among them, adsorptive^5^ and membrane^2^ separations by porous materials remain attractive options, subject to improvement in yield, efficiency, and sustainability^2–4^. Optimal porous materials provide a clear route towards improved performance; however, such a material must be selective of CO_2_ over CH_4_ while also exhibiting CO_2_ uptake sufficiently high for practical use. Many gas separations are characterised by a trade-off between selectivity and uptake^6,7^, rendering search for high-performing materials challenging. Meanwhile, certain separation processes present further complexity. For example, membrane separations additionally rely on effective diffusivity^2^.

Among prominent candidate materials for gas separation are porous metal-organic frameworks (MOFs)^8–11^. High surface area complexes of metal-containing nodes and organic linkers, MOFs have shown excellent performance for a range of chemical processes^12–16^ including several gas separations^15^. Structural variety of MOFs occupies a vast and diverse chemical space of reported synthesised MOFs^17^ and many more proposed hypothetical structures^18,19^, making prediction and tuning of all relevant separation properties inaccessible to experiment. High-throughput use of computational force-field methods provides reasonable uptake and selectivity predictions and fundamental insight into structure-property relationships. Screenings of thousands of MOFs for separation of gas mixtures are readily available in the literature^20,21^, including a recent search of nearly 7000 MOFs for biogas upgrading properties with a focus on membrane separation^22^.

Computational MOFs screenings may be radically changed by development of machine learning (ML) models suitable for predicting gas sorption properties using only features that are cheap to calculate. This can expand the size of databases that can be screened and further reduce computational cost. Similar application of ML has become prominent in every field of materials and chemistry research, with recent advances in the prediction of material properties^23^, solubility^24^, protein structure^25^, and reaction pathway^26^. ML is increasingly being applied to investigate and optimise MOFs for CO_2_ adsorption^27,28^ and gas separation^29–37^, including for CO_2_/CH_4_ separation^38–40^. There are several recent reviews of this growing area^41^.

Some studies have considered the relative performance of different ML methods reporting higher predictive accuracy for non-linear over linear methods^30^. When it comes to feature selection, structural descriptors, which can be readily and cheaply calculated, are favoured, as in the CO_2_/CH_4_ study of ref. ^38^ Linear regression models developed by ref. ^32^ to predict CH_4_ uptake and working capacity appear to perform well using only three features, all of them structural. However, features which capture chemical information such as Henry constants^42^, binding energy or the Voronoi energy introduced by ref. ^29^ can improve the quality of ML models.

Previous ML studies of MOFs tend to be trained on large databases composed primarily of hypothetical MOFs without significant curation^30,38^. While this is a useful way to employ large amounts of data, it risks using unviable MOF structures which display issues relating to structural determination procedures^43,44^ or, in the case of hypothetical MOFs, synthesisability^45^. The importance of data curation is becoming increasingly recognised in the material and chemical domains, with studies showing that well-curated datasets give more accurate and insightful models^46–49^. In this light, curation procedures are gaining traction within high-throughput MOF workflows^43,44,50–52^. Understanding of the capabilities of ML models trained using experimental MOF structures curated for viability is therefore essential.

In this work, we develop a ML model to predict performance of MOFs for biogas upgrading using a well-curated and high quality dataset. One should not underestimate the importance of care in preparation of the dataset. A small number of carefully selected features cover key structural and chemical information. We focus on established (rather than newly invented) descriptors that can provide chemical insight, even if they are, in some cases, relatively expensive to compute. The resultant models are highly accurate and subsequent analysis of the models provides insight into the features of high-performing MOFs for biogas separation.

## Results

### Preparation of a high-quality dataset

A high-quality dataset is an essential prerequisite for ML-assisted high-throughput screening. Here, we use an experimental MOF subset which was obtained from the Cambridge Structural Database (CSD)^53^ as part of a previous high-throughput search for MOFs with biogas upgrading properties by Glover and Besley^22^ in which the dataset was algorithmically stripped of solvents and filtered according to geometric criteria. Visualisation of the initial dataset comprising 6768 stripped MOF structures revealed significant issues necessitating further curation (see Supplementary Note 1). Limitations of datasets which may negatively affect high-throughput and machine learning MOF studies are gaining attention. These include persistence of unfeasible structures^43,44^ as well as persistence of duplicate structures which may affect diversity and lead to data leakage in machine learning^54,55^. Curation procedures improve the quality of MOF datasets, and are beginning to be applied in contemporary studies^43,44,50–52^.

A strict curation workflow, as detailed in the Methods Section 4.1 and shown in Fig. S1 of Supplementary Note 1, was applied to remove unfeasible structures as well as duplicates of the same structure, after which only 1910 MOFs remained in the dataset. The distribution of metal centres present in this dataset is illustrated in Fig. S2 of Supplementary Note 1. The most abundant metal in the dataset is Zn, followed by Cu and Co. The large number of removed MOF structures highlights the critical prevalence of unfeasible structures in MOF databases, which should not be ignored. During the curation stage, overlapping and missing atoms were encountered in nearly 10% of MOFs structures, and the proportion of unrealistic structures increased dramatically when the oxidation state of metal centres was considered (at this stage, more than half of the remaining structures were flagged for removal). The CSD MOF dataset is well-known and commonly utilised, and it is far from the only database which suffers from the issues encountered.

For the remaining MOFs, Grand Canonical Monte Carlo (GCMC) simulations were used to calculate key metrics of biogas separation performance under working conditions of 10 bar pressure and 298 K temperature. Details of the GCMC setup are given in the Methods Section 4.2; the accuracy and reliability of the setup has been validated previously^56^ by comparing its performance for CH_4_ and CO_2_ uptake in the MFM family of copper paddlewheel-based MOFs with existing experimental data^57^. Gas uptake simulations were separately performed to obtain the loading values for both single component (SC) gases and a binary mixture (BM) of 50/50 CO_2_/CH_4_ gas. Selectivity, *S*, of CO_2_ over CH_4_ was calculated from the binary mixture CO_2_ and CH_4_ uptake values, namely, from the loading of CO_2_ and the loading of CH_4_ when a MOF system was simulated in equilibrium with a 50/50 binary mixture of CO_2_ and CH_4_ gases (see Methods section 4.2). Simulation conditions were selected for relevance to the separation within the landscape of industrial conditions which vary depending on specifics of process and materials and can be optimised for a given setup. In particular, the common pressure swing adsorption (PSA) technique tends to require adsorption at high pressures at or above 10 bar^58,59^ and desorption at pressures of 1 bar or below^38,58,60^. Temperature swing adsorption (TSA) can be carried out at ambient pressures^61^ with adsorption temperatures between 273 K and room temperature and desorption temperatures elevated by a margin on the order of 100 K^62^. Membrane separation, meanwhile, uses pressures of a few bar with no demand for elevated temperature^2^. With regard to the selected gas composition, biogas feedstocks vary, with CH_4_ composing 50−65% and CO_2_ composing 35-50% of a mixture (and trace gases also present)^2,22,63^. The 50/50 mixture was selected as an example of a realistic biogas composition comparable to previous work^22^.

**Fig. 1:**
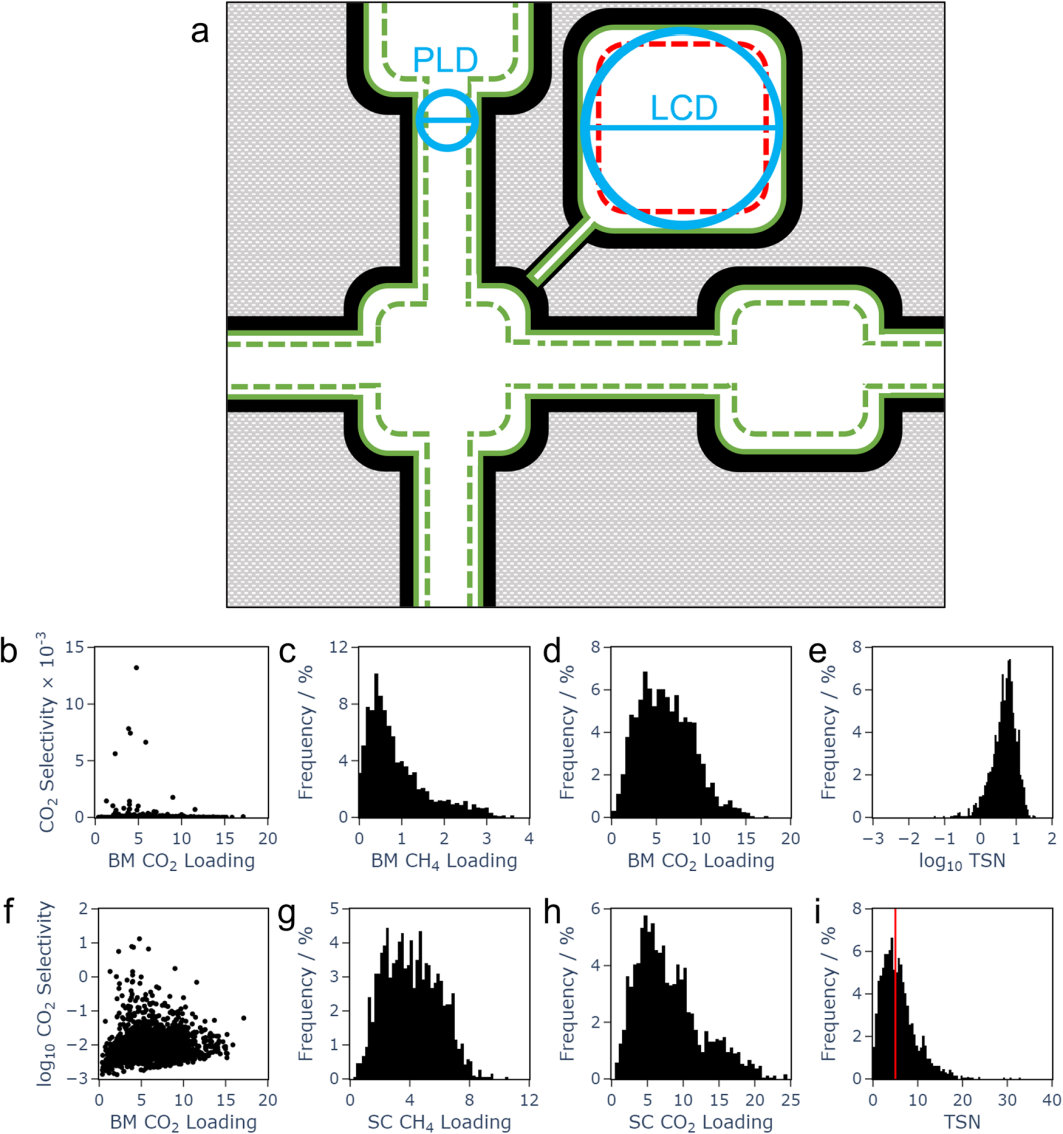
Structural features of MOFs used in the ML model and the range of values for uptake metrics, selectivity and TSN used in the curated data set. **a** An illustration of structural features; blue circles show pore limiting diameter (PLD) and largest cavity diameter (LCD), which describe pore size; green dashed lines show accessible area (ASA) as measured using the centre of a probe (1.86 Å radius), and green solid lines enclose internal volume as measured using a point probe, which is often expressed as a void fraction. Red dashed lines indicate non-accessible area. Plots of binary mixture CO_2_ loading against CO_2_ selectivity, with a (**b**) linear and (**f**) logarithmic scale; (**c**−**e**) and (**g**−**i**) ranges of the six prediction targets studied in this work. Units for loading and TSN are mol kg^−1^; Selectivity is unitless. For the trade off between selectivity and uptake metric, TSN, the indicated cutoff of 5 mol kg^−1^ was used to classify low and high TSN MOFs.

The ranges of the uptake metrics in the curated dataset are illustrated in Fig. 1c, d, g, h which show that single component CH_4_ uptake reaches nearly 12 mol kg^−1^ (Fig. 1g) whilst single component uptake of CO_2_ is notably higher, approaching 25 mol kg^−1^ (Fig. 1h). Binary mixture uptake (Fig. 1c, d) is lower, particularly for the weaker adsorbent CH_4_, although binary mixture CO_2_ loading above 17 mol kg^−1^ is observed, which compares well to previously reported values^64^.

The range of values which selectivity takes in the curated dataset may be seen in Fig. 1b, f, in which a binary mixture CO_2_ loading is plotted against CO_2_ selectivity. Very high values of selectivity above 10^4^ are observed within the curated dataset, although high selectivity can be a consequence of very low CH_4_ loading in MOFs whose CO_2_ loading is not usefully high. Indeed, Fig. 1b, f illustrate the existence of a trade-off relationship between uptake and selectivity. None of the MOFs which display exceptionally high selectivity also have very high loading. An ideal MOF would possess both, and be found in the upper right portion of the plots. Instead, there is a significant population of MOFs in the lower left, branching into the lower right (high loading) and upper left (high selectivity). High uptake and selectivity of the dominant gas are both of importance to gas separations; it is desirable to identify structures displaying both. In the relative absence of MOFs which unite both metrics, it is instructive to identify those presenting a useful compromise between the two, as quantified here by a metric known as trade off between selectivity and uptake, ${\rm{TSN}}_{C{O}_{2}/C{H}_{4}}$^6,7^ (see Methods Section 4.2), which is herein referred to as TSN. The range of values which TSN takes within the curated dataset is illustrated in Fig. 1i.

**Fig. 2:**
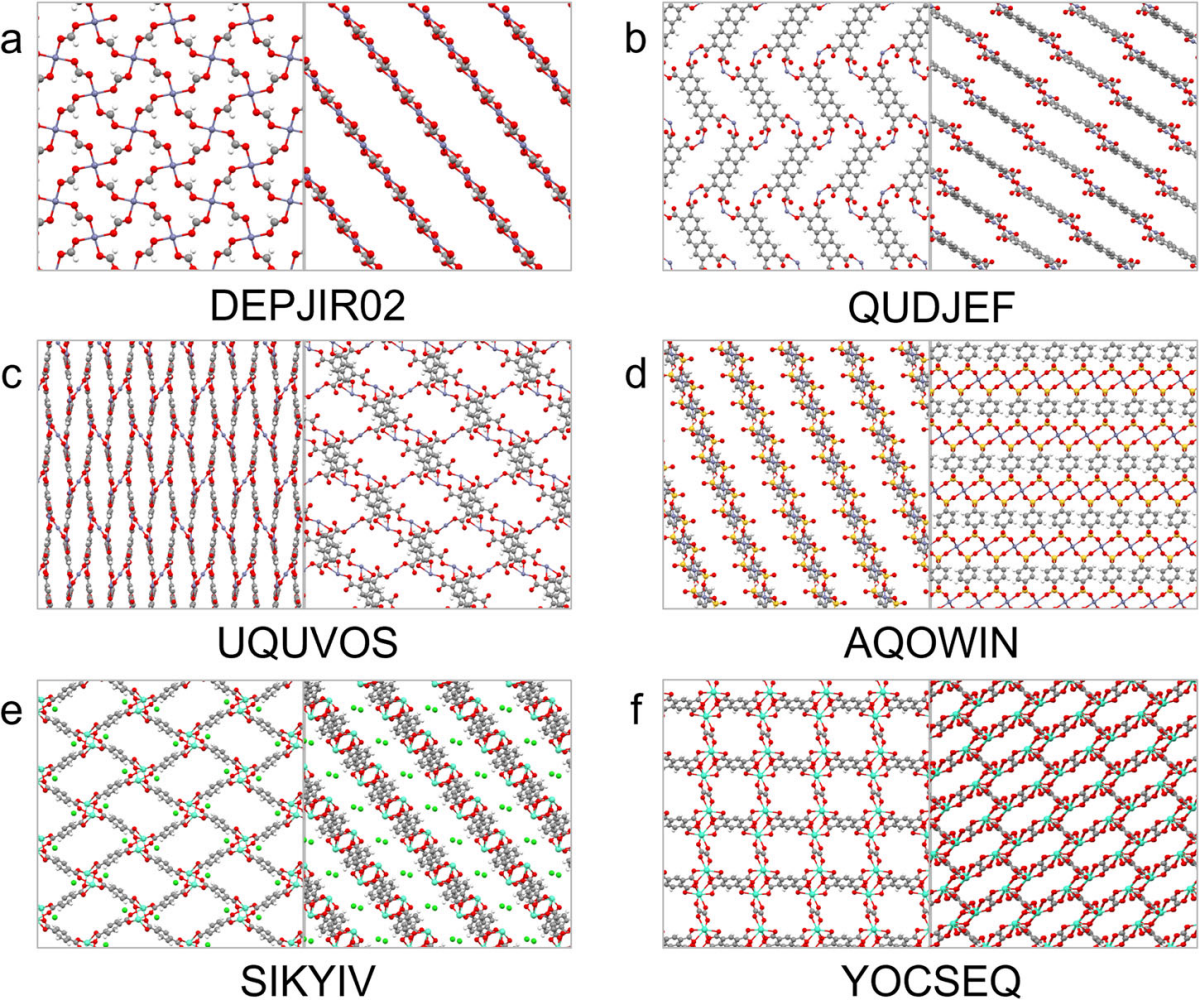
Visualisations of the structures of the six MOFs for which TSN at 10 bar is greater than 22 as predicted by GCMC simulations, visualised along two different axes. **a** DEPJIR02, metal is Zn (**b**) QUDJEF, metal is Zn (**c**) UQUVOS, metal is Zn (**d**) AQOWIN, metal is Zn (**e**) SIKYV, metal is Eu (**f**) YOCSEQ, metal is Cd.

Table 1 gives the names of the six MOFs whose calculated TSN is greater than 22 mol kg^−1^, along with values of TSN, selectivity and binary mixture CO_2_ loading, and ranks of these values within the dataset. It also includes the pore limiting diameter (PLD) and void fraction (VF) of each structure in order to illustrate relationships between structure and uptake properties, as well as the topology of each structure. Structural values were taken from the data of ref. ^22^ and topology was determined using the CrystalNets.jl^65^ web application (https://progs.coudert.name/topology) with the SingleNodes clustering option. To further facilitate detailed structural examination, visualisations of each of the six MOFs are presented in Fig. 2. The six highest-TSN MOFs all rank highly for selectivity, and most also rank highly for binary mixture CO_2_ uptake. Interestingly, many of them possess common structural features. The metal centres of the four MOFs with highest TSN are Zn. Meanwhile, the sql square lattice topology features significantly and several of the six presented MOFs possess approximately square channels. The structures of the five MOFs with highest TSN are two dimensional (2D) except for the MOF with reference code UQUVOS which has a three dimensional (3D) structure made up of connected 2D sheets. Further, the six MOFs have small pore limiting diameter, including two among the four highest-TSN MOFs with PLD very close to 3.80 Å, the kinetic diameter of methane and the smallest PLD allowed in the dataset. VF values range from 0.5 to 0.7.

**Table 1:** Details of the seven MOFs for which TSN is greater than 22 mol kg^−1^ according to the GCMC calculations: refcodes, TSN values, GCMC selectivity values and rank within the dataset, GCMC binary mixture CO_2_ loading and rank within the dataset, pore limiting diameter (PLD), void fraction (VF) and topology^65^

MOF Refcode	TSN /mol kg^−1^	S (rank)	BM CO_2_ Loading /mol kg^−1^ (rank)	PLD /Å	VF	Topology
DEPJIR02	32.86	695.89 (15)	11.56 (80)	3.81	0.66	sql
QUDJEF	30.67	61.50 (90)	17.15 (1)	4.27	0.70	sql^a^
UQUVOS	29.19	1765.15 (6)	8.99 (335)	4.42	0.57	hcb
AQOWIN	23.89	244.94 (30)	10.00 (191)	3.80	0.62	sql
SIKYIV	23.55	52.86 (103)	13.67 (27)	4.30	0.69	sql
YOCSEQ	22.36	6633 (4)	5.85 (940)	4.83	0.50	fsc

^a^QUDJEF was assigned a 2-fold catenated sql topology.

The abundance of Zn centres among the six top-performing MOFs is likely to be at least in part due to the high abundance of Zn-MOFs in the dataset as a whole, in which Zn is the most commonly occurring metal (see Fig. S2). Among 1910 curated MOFs, 542 possess a Zn centre, which amounts to 28% of the dataset. While a significant amount, this is lower than the prevalence of Zn among the top 6 MOFs (67%). It is useful to make similar comparisons to the base dataset for the other structural observations made, to indicate the extent to which they are simply a result of trends in the base data, and to what extent they are specific to the high performers. In terms of dimensionality, 597 of the 1910 MOFs in the base dataset (31%) are 2D, compared to five of the six top performers (83%). For PLD, two of the top performing MOFs have PLD under 3.82 Å, while only 13 of the 1910 MOFs (<1%) have PLD under the same threshold (when rounded). Meanwhile, all of the top performers have PLD under 4.84 Å, compared to 914 (48%) of the curated dataset. For VF, 1230 of the 1910 curated MOFs (64%) fall within the 0.5−0.7 range covered by the top MOFs.

We note that it is possible that some of the identified top MOFs may display flexibility in response to guest adsorption or other external stimuli. There are several modes of flexibility available to MOFs, including the subnetwork displacement mode which applies specifically to 2D layered and 3D interpenetrated structures and involves relocation of separate networks (e.g., layers of 2D MOFs) in relation to each other^66^. While this and other kinds of flexibility can often be useful features of MOF when they are observed, the rigid approximation does not account for them.

The results of the high-throughput GCMC calculations^22^ have been able to identify potentially promising structures for biogas upgrading and provide guidance on structural features which may promote strong biogas upgrading properties. However, acquisition of these results on a large scale is ultimately limited by the computational cost of GCMC calculations. This warrants the development of ML methods able to obtain equivalent information on a shorter timescale. If successful, ML models may allow pre-selection of the most promising MOFs for a targeted property, bypassing large numbers of GCMC simulations and focusing more costly efforts where they are most relevant. Moreover, while structural guidance was provided from the GCMC results by examination of individual MOFs, ML methods may extend this guidance to a larger scale, providing quantitative information about how the structure of a MOF may affect its performance. Development of ML models for prediction of biogas upgrading performance is addressed in subsequent sections.

### Target and descriptor selection

ML methods were developed to make predictions regarding target values relevant to biogas upgrading using only cheaply calculated descriptors of MOF structures and using the GCMC dataset as training data. The target values to be predicted were introduced in the previous section: single component and binary mixture uptakes of CO_2_ and CH_4_, which relate to the affinity of a MOF for each gas, including in the presence of its competitor in the mixture, as well as TSN, which describes the trade-off between uptake and selectivity and is particularly relevant for gas separations. Further details on selection of target values can be found in Supplementary Note 2; Table S1 presents an analysis of the minimum, maximum, mean, and median of the target values, and Figs. S3 and S4 show the distribution of the target values. Initial analysis of the data revealed that the distribution of TSN was highly skewed and likely to present problems when training ML methods. Therefore, log_10_(TSN) was additionally used to reduce the skew and improve the distribution of the data.

**Fig. 3:**
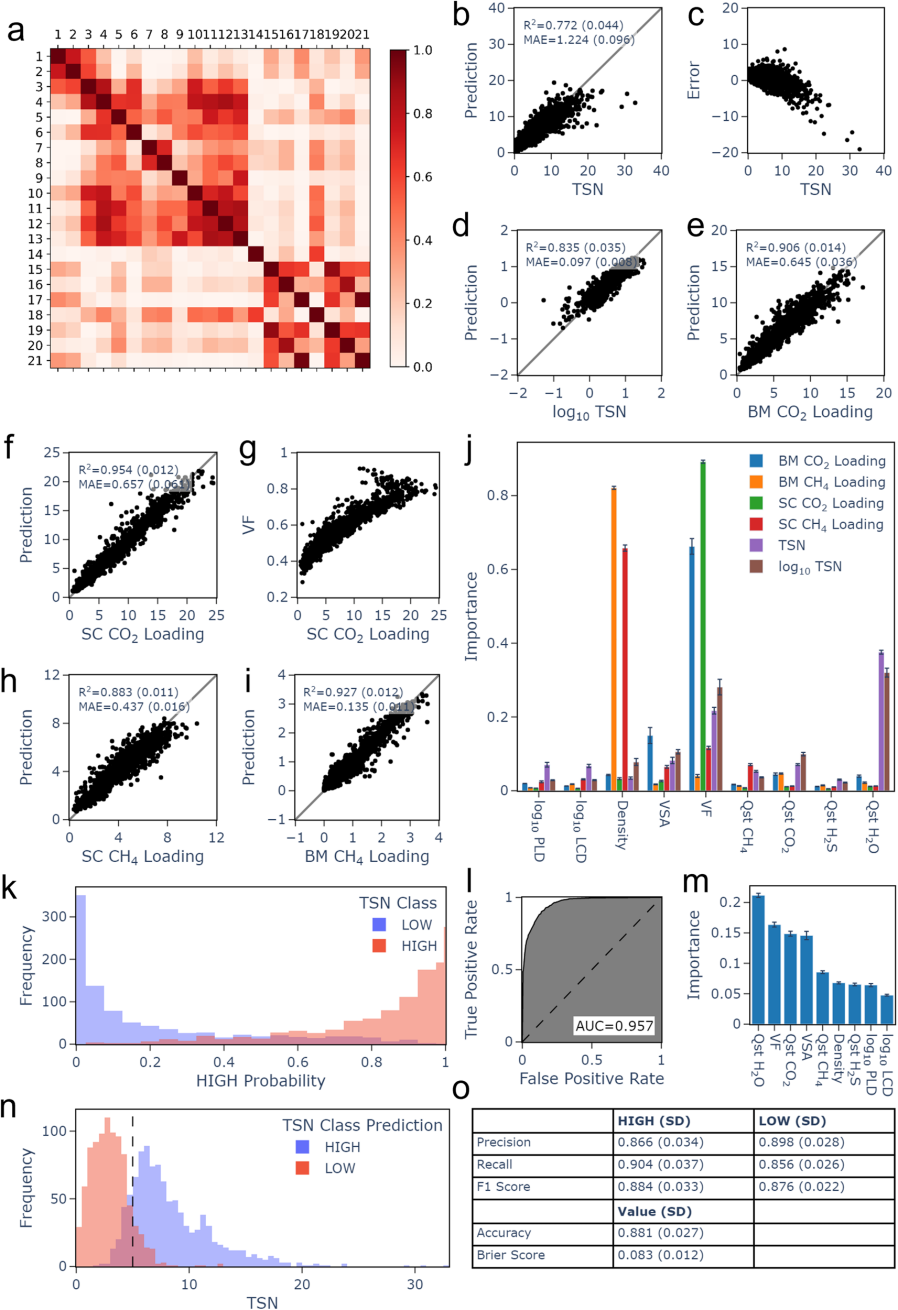
Descriptor selection and random forest results using 10-fold cross validation and nine final descriptors. (**a**) Correlation heat map for descriptors proposed in ref. ^22^ (numbered as in Table S3) (**b**, **d**−**f**) and (**h**, **i**) Regression target predictions (**c**) Error of TSN predictions compared to TSN range (**g**) Relationship between VF, which was identified as the most important descriptor in this model, and SC CO2 Loading. (**j**) Relative importance of each descriptor for each regression target. (**k**) Frequency of HIGH TSN prediction probability compared to the real class. (**l**)TSN classification model receiver operating characteristic curve. (**m**) Relative importance of each descriptor for TSN classification. (**n**) Frequency of HIGH and LOW predictions compared to TSN range. **o** Metrics for TSN classification model. Throughout, the standard deviation across 10 folds is shown in parentheses as a measure of model variability.

Descriptors used as features for a ML model must be cheaply obtained and relevant to the target being predicted. As a starting point for this work, descriptors were taken from the dataset of Glover and Besley^22^ previously used in the early stages of a screening for biogas upgrading with a focus on membrane separation. The 21 descriptors include a mixture of energetic and structural information which has been shown to be desirable for models of this kind^29,42^. Features selected from this set are listed in Table 2 along with the method and software used to calculate them (see other details in Table S6), and the full set of 21 features is similarly described in Table S2 of Supplementary Note 3. Through an analysis of the descriptor values (See Figs. S5–S10 of Supplementary Note 3) it was noted that the distributions of many descriptors were skewed. Hence, to allow the model to discriminate between different descriptor values, a log_10_ scale was applied to pore limiting diameter (PLD), largest cavity diameter (LCD), pore volume (PV) and some chemical descriptors at infinite dilution, namely, Henry constant (K_0_), diffusion coefficient (${\rm{D}}_{0}^{C}$), and permeability (*P*_0_). For any pair of correlated descriptors (Fig. 3a, also Table S5 and Fig. S15), only one was retained. This led to the removal of K_0_ descriptors in favour of heat of adsorption, ${\rm{Q}}_{0}^{st}$, and the removal of pore volume and GSA surface area in favour of void fraction (VF). Analysis and distribution of the log_10_ scaled descriptors can be found in Table S4 and Figs. S11−S14 of Section 3.2 in Supplementary Note 3. Full correlation analysis is presented in Section 3.3 of Supplementary Note 3 (Figs. S16−S21).

**Table 2:** The 9 descriptors used to train the ML model in this work, selected from among the 21 descriptors of Glover and Besley^22^

Descriptor	Description	Method	Software
PLD/Å	Diameter of the largest sphere that can percolate through the MOF	Voronoi network	Zeo++^69^
LCD/Å	Diameter of the largest sphere that fits inside the MOF	Voronoi network	Zeo++^69^
Density/g cm^−3^	Mass of MOF per unit volume		Zeo++^69^
VSA/m^2^ cm^−3^	Surface area accessible to the centre of a probe (r = 1.86 Å) per unit volume	Voronoi network MC sampling	Zeo++^69^
VF	Fraction of the volume not occupied by MOF atoms (calculated with point probe)	Voronoi network MC sampling	Zeo++^69^
${\rm{Q}}_{0}^{st}$ (CH_4_)/kJ mol ^− 1 ^	Heat of adsorption of CH_4_ in the MOF at infinite dilution	Force fields GCMC	RASPA^72^
${\rm{Q}}_{0}^{st}$ (CO_2_)/kJ mol ^− 1^	Heat of adsorption of CO_**2**_ in the MOF at infinite dilution	Force fields GCMC	RASPA^72^
${\rm{Q}}_{0}^{st}$ (H_2_S)/kJ mol ^− 1^	Heat of adsorption of H_2_S in the MOF at infinite dilution	Force fields GCMC	RASPA^72^
${\rm{Q}}_{0}^{st}$ (H_2_O)/kJ mol ^− 1 ^	Heat of adsorption of H_2_O in the MOF at infinite dilution	Force fields GCMC	RASPA^72^

Where relevant: MC = Monte Carlo, GCMC = grand canonical Monte Carlo, MD = molecular dynamics, r = probe radius.

In summary, only a small number of relevant descriptors, given in Table 2, was selected to allow the ML model to be transparent and easily interpreted^24,67^, and we used our understanding of gas selectivity and uptake to remove the less appropriate descriptors. Diffusion coefficients of gases and permeability at infinite dilution were removed as these descriptors relate specifically to membrane separations. The values used as targets in this study are thermodynamic properties, which, although relevant to several industrial applications, do not alone describe industrial workflows like membrane separation. Furthermore, the diffusion coefficients and permeability descriptors are obtained using molecular dynamics simulations and come with an additional computational cost (and, hence, reduced training efficiency) as compared to the other descriptors.

### Random forest results

The complete ML protocol used in this study can be found in Fig. S22 of Supplementary Note 4. Plots of predictions using each ML method for each target can be seen in Figs. S23−S25. The corresponding plots of error against target range can be seen in Figs. S26−S28. Machine learning results using random forest are shown in Fig. 3 using 10-fold cross validation. Additional models were built using Multiple Linear Regression (MLR), Support Vector Machine (SVM), and k-Nearest Neighbours (kNN); results using these methods can be found in Supplementary Note 5. For the six regression targets, RF and SVM gave comparable results, outperforming MLR. RF models gave mean absolute error in the range of 0.097 to 1.224, and in all cases smaller than the standard deviation of the target values, showing the utility of the models. The best models were built for single component and binary mixture CO_2_ loading and RF (Fig. 3e, f) with *R*^2^ > 0.9. The predictions for TSN (Fig. 3b, c) are very skewed, giving large underpredictions for higher TSN MOFs.

Thus, we see that the regression models are able to predict uptakes more efficiently than TSN (or indirectly selectivity), which is itself ultimately based on uptake values. This likely relates to the fact that for the training set, TSN data are skewed to low values, and also to propagation of errors. Specifically, the equation for calculating selectivity (Eq (1) of the Methods section) involves division by the quantity of adsorbed CH_4_ in a binary mixture simulation. Calculated CH_4_ loading values are generally small, and prediction errors, which may be small when predicting only CH_4_ loading, are amplified, lowering performance of the model. Meanwhile, a small number of CH_4_ loading values are particularly small, resulting in a select few unusually high selectivity and TSN values and the observed low-TSN skew in the training data. In order to resolve this, additional classification models were built for TSN with the aim of identifying high and low TSN MOFs, an important task in MOFs screening. The benchmark TSN value of 5 mol kg^−1^ was selected to differentiate between high and low TSN MOFs. The selection of the benchmark value is discussed in Section S2.1 of Supplementary Note 2.

The classification models for TSN gave excellent results for kNN, SVM and RF, with RF giving the best accuracy of 0.881 and area under the receiver-operator curve (AUC) of 0.957 (Fig. 3l, o; also see Figs. S29 and S30). The few misclassifications for RF were localised around the interface between high TSN and low TSN, as shown in Fig. 3n where the class prediction is presented against the actual TSN. Almost all the incorrect classifications are within ± 2 mol kg^−1^ TSN of the interface. The probability of assigning to each class was examined against the real class (Fig. 3k). It was observed that the more confident the model, the more likely the the correct class had been predicted, with very few cases of high confidence resulting in an incorrect prediction. The complete ML protocol used in this study can be found in Fig. S22 of Supplementary Note 4, and the full metrics for regression and classification models can be seen in Table S7 of Supplementary Note 5.

## Discussion

### Interpretability of machine learning predictions

To interpret the RF models, the average descriptor importance was calculated from the models (Fig. 3j, m). For the regression models, density of the MOF was important for predicting single component and binary mixture CH_4_ loading, whilst volume fraction available for gas uptake in the MOF was the key descriptor for single component and binary mixture CO_2_ loading, and heat of adsorption of water in MOF at infinite dilution, ${\rm{Q}}_{0}^{st}$ (H_2_O), for TSN. The strong influence of volume fraction on single component CO_2_ loading was examined by plotting void fraction against single component CO_2_ loading (Fig. 3g), indicating that single component CO_2_ loading increases with void fraction up to a certain point, but for the few MOFs with very high void fraction, single component CO_2_ loading begins to decrease again. Although the correlation between this descriptor and the target is strong, the model that contains 9 descriptors (Fig. 3f) gives more accurate predictions than using VF alone.

We find that for the most important features, particularly for the TSN targets, a mixture of structural and energetic descriptors is required, supporting evidence from previous studies^29,42^. For the gas uptake targets specifically, structural features dominate whilst energetic features contribute relatively little to the machine learning models. Among the structural features, void fraction stands out particularly as having very high importance for CO_2_ loading and strong contribution to a number of the other models, most prominently to the TSN classification model and the TSN regression models (CO_2_ loading contributes directly to TSN). The dependence of CO_2_ loading on void fraction indicates that under the 10 bar conditions studied, available pore space is relevant to how readily new gas molecules may enter the MOF as unfavourable arrangements of guest molecules are necessitated. Indeed, some of the smaller-VF MOFs may have already reached saturation by this point, meaning that only structures with sufficient VF are able to continue to adsorb. Meanwhile, Fig. 3g indicates that at the highest void fraction values in the dataset, adsorption is reduced compared to at the most favourable void fractions. Here, interactions with pore walls are decreased and adsorption is reduced.

It is interesting that the energetic feature displaying the most overall importance for predicting the target values is heat of adsorption of water, *Q*_*st*_ (H_2_O), which provides a description of the hydrophilicity and polarity of a MOF structure. Such a description accounts for the selectivity contribution to TSN, with highly polar MOFs likely to unite affinity for CO_2_ with lack of affinity for CH_4_. For both the RF regression and the classification models, Q_*st*_ (H_2_O) provides a more important description of TSN than the heat of adsorption of either of the two individual gases for both the regression and the classification models.

The models were further analysed by examining the best- and worst-predicted MOFs for structural similarities. This was to determine the types of MOFs for which the models provided excellent and poor predictions. The analysis for the regression model with the best overall performance, random forest single component CO_2_ loading, and the TSN random forest classification model can be found in Supplementary Notes 5 and 6 (see analysis of the outliers in Tables S10 and S11). For the CO_2_ regression model, the majority of the largest errors correspond to an overprediction of the loading in MOFs displaying very high void fraction. Meanwhile, the majority of the best-predicted structures possess narrow pores, with smaller pore limiting diameter than the poorly predicted MOFs. For the classification model, void fraction also seems to display a relationship with prediction quality, with particularly well-predicted MOFs having void fraction falling largely within defined ranges, and low-performing MOFs which were incorrectly classified as high-performing displaying larger void fraction values. Pore limiting diameter varies among both correctly classified and incorrectly classified MOFs, but higher maximum pore limiting diameter is seen for the poorly classified MOFs than for the well-classified structures.

In the context of a MOF screening, it is instructive to ascertain the performance of a model for the MOFs displaying the best performance. In this case, therefore, the six MOFs identified as most promising for biogas upgrading by GCMC and identified in Fig. 2 are considered. Since these MOFs were identified based on TSN, the results of the RF TSN classification model were checked. It was confirmed that the TSN classification model correctly classified all six identified top-performers, all with high-performing probability above 0.9 with the exception of YOCSEQ (probability=0.63), suggesting that the model is successful in correctly classifying the very high-performing MOFs most desired in a screening.

### External test data

The best-predicted regression target, single component CO_2_ loading, and the TSN classification model were retrained using the full training set and their performance was tested on a dataset of unseen MOFs originating from an external source. To generate this dataset, 1000 structures were selected at random from the Northwestern hypothetical database^18^ and subjected to the same curation procedure applied to the training data, including the application of geometrical and charge criteria used by ref. ^22^ and with the addition of a check for similarity to the training set to avoid data leakage^54,55^. No MOFs were found to be identical to training set structures. The curation left a total of 330 structures in the external test dataset. The distribution of metals present in these MOFs is shown in Fig. S2 Supplementary Note 1; the dataset is heavily dominated by Zn and Cu MOFs. The nine selected descriptors were calculated for each MOF as described in the Methods section to allow predictions to be made using the ML models. Relevant targets were also calculated using GCMC as described in the Methods section for comparison to predictions. The unseen test set descriptors and targets were analysed by the same statistics as the training set, as shown in Table S8. The ranges distributions of the descriptors and targets are shown in Figs. S33−S37. The correlation of the unseen test set descriptors can be found in Fig. S38. The full metrics for test set predictions are shown in Table S9.

**Fig. 4:**
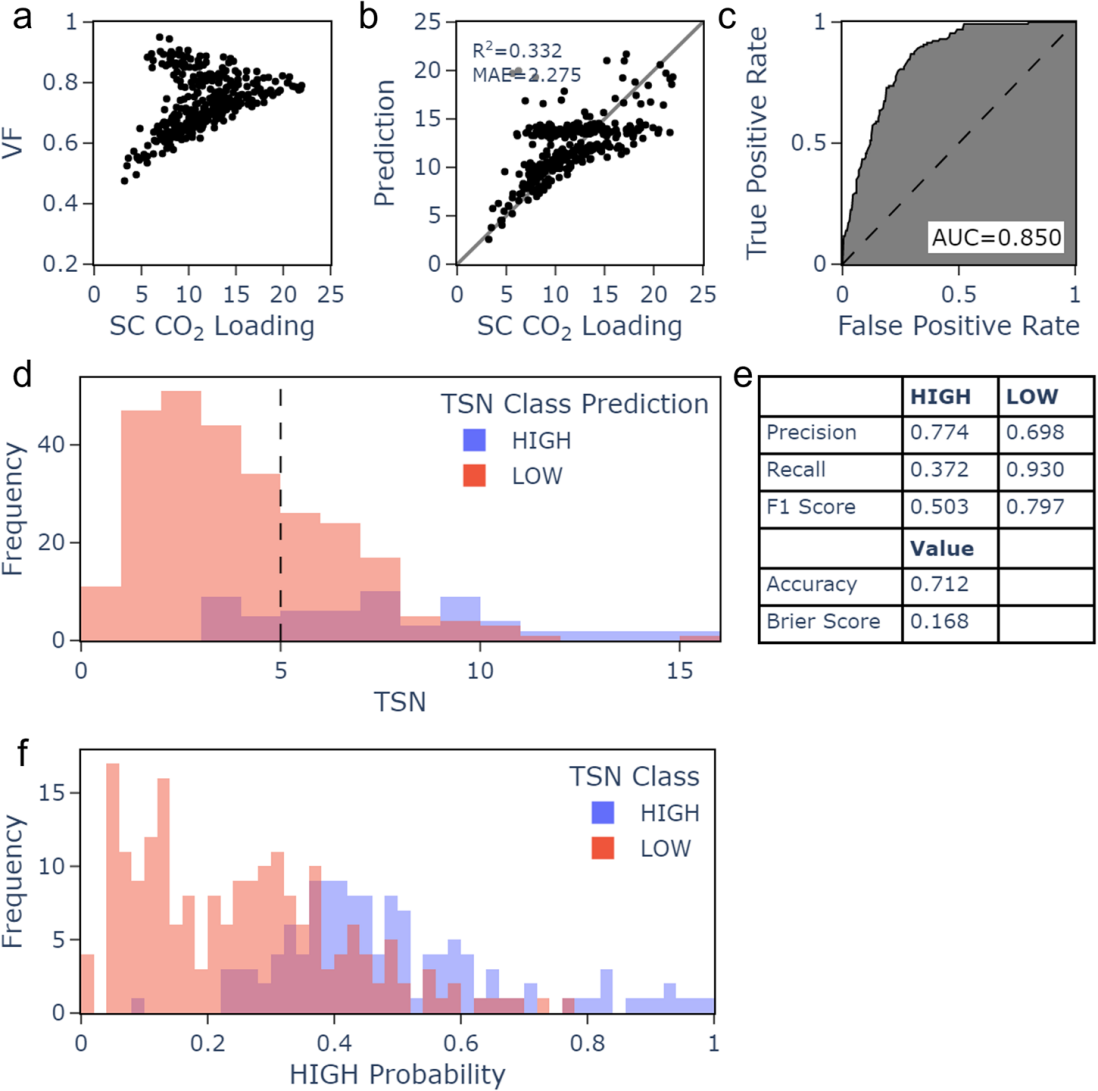
External test set results using random forest retrained on the full training set and 9 final descriptors. (**a**) Relationship between VF and SC CO_2_ Loading (**b**) Predictions for SC CO_2_ Loading (**c**) Receiver-operator characteristic curve for TSN classification. (**d**) Frequency of HIGH and LOW predictions compared to TSN range. (**e**) TSN classification metrics. (**f**) Frequency of HIGH TSN prediction probability compared to the real class.

The results are shown in Fig. 4 for RF. The mean absolute error for regression models was lower for RF (2.275) than SVM or MLR, and much lower than the standard deviation of the external test data (3.796). This model had a modest *R*^2^ of 0.332. However, an examination of Fig. 4a, b shows that the modest *R*^2^ is due to underprediction of mid and high loading MOFs. Interestingly, the correlation between single component CO_2_ loading and void fraction is different to the training set (Fig. 3g) and, due to the predictions’ strong dependence on void fraction, the predictions are affected accordingly. For the TSN classification models, accuracy (0.712) and area under the curve (0.850) was found with RF, with comparable results for RF and kNN. The recall for HIGH MOFs was much lower than the cross validation results, partially due to the low number of HIGH MOFs in the external test set. The model was particularly good at identifying LOW MOFs (Fig. 4d, e). However, several MOFs with very high TSN were predicted LOW. The model was good at assigning the correct label when the probability was < 0.2 or > 0.8 and less so at intermediate values. To further assess the importance of each descriptor in the model, random forest models were rebuilt for the regression targets with 10-fold cross validation leaving out each descriptor in turn (see Section 5.4 of Supplementary Note 5). Figs. S31 and S32 show the mean metric and the standard deviation error across the 10 folds. Models were also trained with the full training dataset and tested on the unseen test set. The full prediction and error plots can be seen in Figs. S39−S44. The classification models were retrained using the full training set and tested on the unseen test set. The analysis for the cross validation was repeated and can be seen in Figs. S45 and S46.

The underprediction of the performance of HIGH MOFs displaying high void fraction illustrated in Fig. 4a, b for the external test set appears to be a more extreme case of similar underprediction observed for high-void fraction MOFs in the training and validation sets. Comparison of Fig. 4a alongside Fig. 3g reveals that for both the validation set and the test set there is a peak in single component CO_2_ loading at a void fraction of around 0.8, and that the test set contains proportionally many more points above this peak than the validation set does. This offers an explanation for the difference between the model performance for the training set and for the external test set: the test set contains more MOFs with particularly high void fractions leading to comparatively low loading. With fewer of these kinds of MOFs seen in training, model predictions are poorer. The feature space covered by the external test set, which is composed of hypothetical MOFs, is not the same as the feature space the models were trained on. In addition to providing valuable information about the performance of the trained models, this highlights an interesting point about the merits of detailed study of the similarities between commonly used hypothetical and real sets of MOFs. The benefits of hypothetical MOF databases are defined by the fact that they contain MOFs which have not been produced experimentally, so some level of dissimilarity between the two is advantageous. However, a database which significantly departs from structures similar to those known to be experimentally synthesisable risks containing sets of coordinates which are not useful to pursue. It is vital to understand the strengths and limitations of hypothetical MOF databases. This was partially addressed in work by ref. ^68^ comparing the feature space covered by MOF databases. The work identified differences between hyopthetical and real datasets and a lack of diversity in hypothetical MOFs (including, as seen in this work, a limited number of metal centres present in hypothetical MOFs), with resulting implications on screening conclusions. Consistent scrutiny of hypothetical MOF databases is needed as they continue to be widely utilised in material design and selection.

**Fig. 5:**
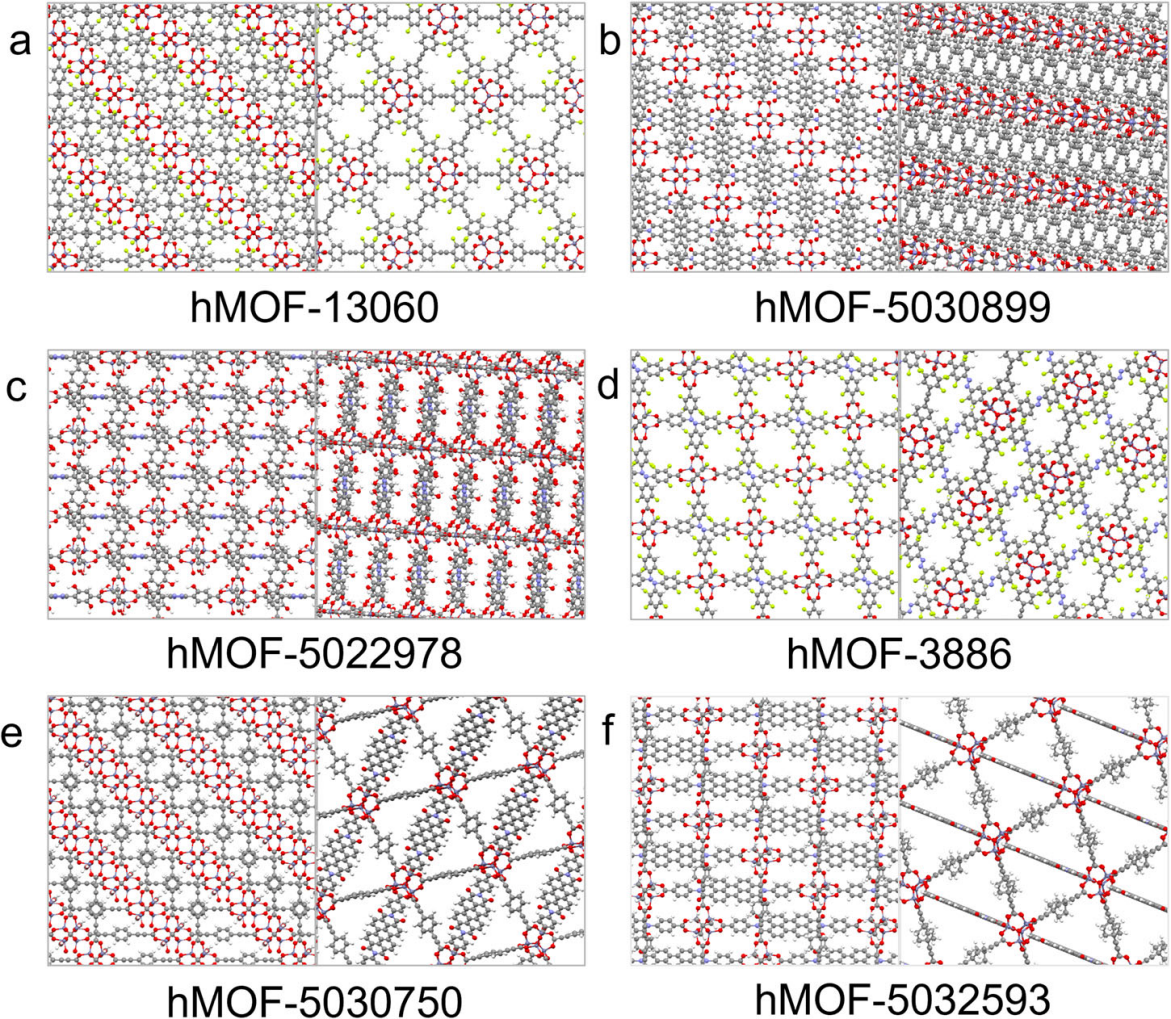
Visualisations of the structures of the six MOFs predicted to have high TSN with the highest probability among the external test set by the random forest classification model. The MOFs are (**a**) hMOF-13060, metal is Zn (**b**) hMOF-5030899, metal is Zn (**c**) hMOF-5022978, metal is Zn (**d**) hMOF-3886, metal is Zn (**e**) hMOF-5030750, metal is Zn (**f**) hMOF-5032593, metal is Zn.

To identify the MOFs likely to be best performing according to the predictions of the random forest ML model, the six MOFs from the external test set classified as high TSN with the highest probability are detailed in Table 3 and visualised in Fig. 5. Table 3 also includes relevant geometrical features PLD and VF, and includes topology^65^ determined as described in section 2.1, as well as degree of interpenetration, also determined using CrystalNets.jl^65^. Interpenetration is widely observed among the identified promising hMOFs. Similarly, the six MOFs predicted by the random forest regression model to display the highest single component CO_2_ loading are detailed in Table S12 and visualised in Fig. S47 of Supplementary Note 7. In general, the predictions are fairly successful, with the predicted top TSN MOFs displaying high GCMC TSN, clearly above the threshold of 5 mol kg^−1^ and the predicted top loading MOFs displaying predicted loading close to their GCMC values. Several of the top MOFs for both properties display structural similarities: all top-TSN MOFs and most top-SC CO_2_ loading MOFs possess interpenetrated frameworks based on the pcu (primitive cubic) lattice. Higher degrees of interpenetration are observed for the top-TSN MOFs than for the top-uptake MOFs. For the uptake model, the top MOFs have void fraction very close to the value of 0.8 which corresponds to the loading peak previously mentioned. All 6 of the MOFs predicted to display the highest single-component CO_2_ loading have VF between 0.79 and 0.87, in common with 86 (26%) of the 330 MOFs in the external test set. Meanwhile, 5 of these 6 (83%) have VF between 0.79 and 0.82, in common with 47 (14%) of the 330 external test set structures. For the TSN model, the top MOFs generally possess VF lower than 0.8. This is true of all six predicted top-TSN MOFs, in common with 242 of the 330-strong test set (73%). Five of the six top predictions have VF lower than 0.72, in common with 145 (44%) of the 330 test MOFs. A range of values for pore limiting diameter is observed, in line with the lower feature importance of this metric, although top MOFs for loading generally have higher pore limiting diameter than top MOFs for TSN. The six top-SC-CO_2_ loading MOFs have PLD ranging from 6.69 Å to 11.5 Å, a range covered by 109 (33%) of the full curated test set, while the six top-TSN MOFs have PLD ranging from 4.40 Å to 6.69 Å, a range covered by 154 (47%) of the full curated test set. None of the top predicted MOFs are two dimensional; the external dataset of hypothetical MOFs contained only 3D structures. All of the top six TSN MOFs and the majority of the top six MOFs for single component CO_2_ loading (4 of the 6, 67%) possess Zn centres. This is in line with similar observations seen in the training and validation set, but must largely be attributed to the significant dominance of Zn centres in the test set (247 of 330, or 75%, see Figure S2 of Supplementary Note 1).

**Table 3:** Details of the six MOFs predicted to display high TSN with the highest probability among the external test set according to the RF classification model: the identifier, probability, calculated (GCMC) TSN, pore limiting diameter (PLD), void fraction (VF), topology, and degree of interpenetration (DI)

Numerical	High TSN	GCMC TSN	PLD	VF	Topology	DI
Identifier	probability	/mol kg^−1^	/Å			
13060	1.00	11.0	4.71	0.62	pcu	4
5030899	0.994	9.60	5.11	0.67	pcu	4
5022978	0.966	6.33	5.26	0.71	pcu	3
3886	0.956	12.2	6.69	0.79	pcu	2
5030750	0.938	10.9	4.40	0.65	pcu	4
5032593	0.930	7.04	6.42	0.71	pcu	3

## Methods

### Dataset curation and cleaning

The curation workflow used to refine the dataset to contain only physically viable MOFs is outlined below. Further details are given in Supplementary Note 1. Structures containing no metal, no carbon or only one or two elements were first identified and removed from the initial dataset taken from ref. ^22^ of 6768 MOFs, leaving a total of 6663 structures. To combat overlapping atoms, any structures with any atom-atom distances less than 0.5 Å were next removed, leaving 6,638 structures in the dataset. Any D atoms present in any crystallographic information files were replaced with H and then all files with no H were removed, leaving 6359 structures. Issues dealt with up to this stage would be clear from visualisation.

Oxidation state counting was then used to remove structures with unviable oxidation states using a published MOF Oxidation State And Electron Count (MOSAEC) code^44^. The MOSAEC code was applied to all 6359 remaining structures and any that were flagged with any features indicating problematic oxidation states were removed. A total of 3553 structures were flagged as problematic, leaving only 3086 MOFs in the dataset. A filter for dimensionality was then applied: the dimensionality of each of the 3086 structures was determined algorithmically using the Zeo++ software package^69^. Of these, 1715 3D structures, 686 2D structures, 611 1D structures and 74 0D structures were identified. All 1D and 0D structures were removed from the database, leaving a total of 2401 MOFs. Of these 2401 MOFs, charge equilibration calculations necessary for the simulations were unable to complete for 20 structures, leaving a total of 2381.

A final step was removal of duplicate structures^54,55^. Weisfeiler-Lehman structure hashes for undecorated (agnostic to atom type) structural graphs of each MOF were obtained using the Pymatgen materials analysis python library^70^ and the NetworkX package^71^. For any groups of structures with identical hashes, only one MOF was retained. Additionally, groups of structures whose CSD refcode is based on the same six-letter string are based on the same MOF, possibly with changes to the synthesis procedure, and are therefore expected to by highly similar. Only one MOF was retained for each six-letter string. Following deduplication, and therefore following the full curation procedure, 1910 MOFs remained in the CSD dataset. An equivalent curation procedure was applied to the external test set, yielding 330 curated structures.

### Calculation of descriptors and labels

Computational details of the Monte Carlo setup used within the RASPA software package^72^ to determine absolute gas uptake are outlined below. Interactions between components of the system were modelled using Lennard-Jones potentials, considering host-guest and guest-guest interactions, but no host-host interactions. The guest molecules CO_2_ and CH_4_ were modelled using the TraPPE formalism, in which CO_2_ is treated with a 3-site model with each site possessing a partial charge and Lennard-Jones parameters, and CH_4_ is treated using a single site model with no charge but with Lennard-Jones parameters. Lennard-Jones parameters for the framework were taken from the Universal Force Field (UFF)^73^. Framework partial charges necessary to model interactions of framework atoms with CO_2_ were determined prior to the uptake simulations using the extended charge equilibration (eQeq) method available within the RASPA software package (version 2.0.35)^72^.

During GCMC simulations, van der Waals interactions were calculated with a cutoff of 12.8 Å, above which the potential was shifted to zero without tail corrections. Often, tail corrections can be a convenient choice to make the adsorption results less sensitive to the details of the truncation^50^. Unlike atoms were treated using Lorentz-Berthelot mixing rules. Sufficient replicas of the unit cell of each MOF were used so that all perpendicular distances were at least 25.6 Å (two times the cutoff). Electrostatic interactions where required were calculated using the Ewald method with precision of 10^−6^. The MOF system was in equilibrium with an imaginary reservoir of gas molecules, either pure CO_2_, pure CH_4_ or a CO_2_/CH_4_ mixture with each gas present with a mole fraction of 0.5. Gas phase fugacities of the components were calculated using the Peng-Robinson equation of state. The temperature used was 298 K and the external pressure modelled was 10 bar. Monte Carlo moves available to the system were translation of a guest, rotation of a guest, insertion or deletion of a guest (grouped together by the software under a move called swap) and in the binary mixture case changing a guest’s identity from CO_2_ to CH_4_ or the reverse. Simulations were run for 10,000 equilibration cycles followed by 10,000 production cycles, where the number of steps in a cycle is equal to the number of molecules in the system, or to 20 if there are fewer than 20 molecules in the system. The computational cost of these GCMC calculations of gas adsorption under relevant conditions varies depending on the MOF considered. The time for an uptake calculation to complete for a single MOF ranges from several hours to multiple days when running on a high-performance compute cluster possessing an Intel Xeon Gold 6138 20C 2.0 GHz CPU.

Further separation metrics are determined from binary mixture CO_2_ and CH_4_ loading. Selectivity, *S*, quantifies the preferential adsorption of CO_2_ over CH_4_ and is given by Eq. (1), where *q*_*i*_ is the quantity of gas *i* in the adsorbed phase, and *y*_*i*_ is the mole fraction of gas *i* in the gas phase.

1 $${S}_{C{O}_{2}/C{H}_{4}}=\frac{{q}_{C{O}_{2}}{y}_{C{H}_{4}}}{{q}_{C{H}_{4}}{y}_{C{O}_{2}}}$$

$TS{N}_{C{O}_{2}/C{H}_{4}}$ quantifies the trade-off between ${S}_{C{O}_{2}/C{H}_{4}}$ and binary mixture CO_2_ uptake. It is given in equation (2), and is referred to in this work as TSN.

2 $$TS{N}_{C{O}_{2}/C{H}_{4}}={N}_{C{O}_{2}}\,{{\mbox{log}}}\,({S}_{C{O}_{2}/C{H}_{4}})$$

Descriptors for the training data used in this study were taken from a previous high-throughput screening of the training MOFs for membrane separation of biogas mixtures^22^. Equivalent descriptors were calculated for the external test set as follows, mirroring the setup used in the previous screening for consistency.

Structural descriptors, PLD, LCD, density, VSA, GSA, VF and PV, were calculated using the Zeo++ software package using high accuracy settings in all cases^69^. Surface area was calculated with a probe radius of 1.86 Å and with 5000 Monte Carlo cycles. This is the size of a spherical probe conventionally used to represent the N_2_ molecule, which is commonly used for experimental surface area measurements^74^. It is also consistent with the methods used by Glover and Besley to generate other data used in this work^22^. Volume was calculated with a point probe and 50,000 Monte Carlo cycles. Structural features for a single MOF can be calculated within seconds. The energetic features, infinite dilution heats of adsorption, were calculated using the Widom insertion method with 100,000 Monte Carlo cycles at 298 K. Moves available for selection were translation, rotation and reinsertion. A heat of adsorption value can be obtained in under half an hour.

### Machine learning

Machine learning was implemented with *Python/Scikit-learn*^75^. Separate models were built for the six regression targets and one classification target using Support Vector Machine and Random Forest. Additionally, for regression Multiple Linear Regression was used and for classification k-Nearest Neighbours were also built. The number of trees used for Random Forest models was 500, with the default values used for all other parameters. Hyperparameters for the Support Vector Machine models were optimised using the training set and a 5-fold cross validation grid search within the 10-fold cross validation protocol (nested cross validation) to avoid data leakage. From a wide range of values, the optimal values for *γ* and *C* were found for all models, in addition to the optimal *ϵ* value for regression models. *C* is the regularisation parameter and specifies the penalty for an incorrect prediction. *γ* is the kernel coefficient, a term in the kernel which transforms the data in the algorithm. *ϵ* is a term in the regression model which specifies the allowed error between the data and function for which no penalty is applied. The kernel was set to the radial basis function (rbf) and the default values were used for all other hyperparameters. The optimal number of neighbours for k-Nearest Neighbours was optimised between 1-99. Models were initially tested using 10-fold cross validation using the full dataset. By analysing the predictions for each fold, average metrics were calculated with the error as the standard deviation of the metrics across the 10 folds. For regression models, Mean Absolute Error (MAE) and *R*^2^ score (coefficient of determination) were used as metrics. For the TSN classification model, both the predicted class and probability of that class were obtained, where HIGH was assigned the positive label, i.e., a probability of 0.9 denoted 90% confidence the instance belonged to the HIGH class and 10% confidence the instance belonged to the LOW class. Models were assessed with the precision, recall, F1 score of the classes and the overall accuracy of the models. In addition, the Brier score, the receiver operating characteristic (ROC), and the resultant area under the curve (AUC) were calculated from the class probabilities. For full explanation of metrics used in this work, see Supplementary Note 4. The average importance of each descriptor was assessed using the standard protocol employed in the feature_importances_ attribute of each RF model in the 10-fold cross validation. Again the error was the standard deviation of the importance across the 10 folds. The optimal hyperparamters were recalculated using 5-fold cross validation for the full dataset. These parameters were used to train models using the full dataset. The resultant models were used to predict targets for the external test data. These predictions were analysed using the same methods mentioned above.

## Conclusions

In this work, GCMC simulations have been used to obtain computational measures of biogas separation metrics in a range of curated MOF structures with a view to identify MOFs promising for biogas upgrading. Highly accurate machine learning models have been trained to make predictions regarding these metrics efficiently and with a lower computational cost than GCMC. Regression models were trained to predict CO_2_ and CH_4_ loading, and classification models were trained to classify MOFs as high-performing or low-performing. Models were trained on a carefully curated set of real structures, ensuring the data was high quality. An advantage of machine learning is that after the model has been trained and the descriptors have been calculated, predictions are almost instantaneous. The descriptors used can be computationally obtained several times more cheaply than the biogas upgrading metrics predicted. The machine learning protocol therefore represents a clear cost reduction for high-throughput screening compared to a conventional GCMC-only approach.

The models were extensively tested both on validation data and on an external test set of hypothetical MOFs. Models displayed strong performance, especially for CO_2_ and CH_4_ loading regression models and TSN classification models, with random forest models showing the best overall performance. Predicting an external test set of hypothetical MOFs was challenging. This can be rationalised by the distribution of the training and test sets, which do not cover precisely the same areas of target and feature space. There is therefore scope for future improvement of supplementing the training data to increase the coverage of the models. The difference between the performance of the models on data from different sources also highlights the diversity of data between different kinds of MOF databases, and the differences between hypothetical and real MOF datasets.

The results of both the GCMC simulations and the machine learning models were used to identify selected MOFs which may be promising for biogas upgrading, and also to identify structural features which are common to high-TSN MOFs. It was seen that 2D MOFs with narrow separation between sheets, as well as interpenetrated frameworks based on square or cubic topology, are among those which may be useful for the application. It was also seen that void fractions around 0.8 facilitate optimum uptake of CO_2_ while slightly lower void fractions around 0.7 facilitate an optimum trade-off between uptake and selectivity.

## Model availability

In order to use the model, first descriptors should be calculated by following the steps described in Section 4.2. All the data and code required to run the models presented in this work is available on GitHub at https://github.com/samuel-boobier/ML-MOFs. In addition, simple instructions of how to reproduce the models or make predictions for your own test sets are given. Finally, sample RASPA input files and curation procedures are provided.

## Supplementary information

Supplementary Information

## Data Availability

All correspondence and material requests should be made to Professor Elena Besley, School of Chemistry, University of Nottingham, University Park, Nottingham, NG7 2RD, United Kingdom. Email: Elena.Besley@nottingham.ac.uk. Full details of the code and datasets used in this work can be accessed at https://github.com/samuel-boobier/ML-MOFs. Further analysis of the data and models presented in this work are provided as Supporting Information.
