# Supplementary material for: Machine learning insights into predicting biogas separation in metal-organic frameworks
Source: Commun Chem. 2024 May 8;7:102. doi: 10.1038/s42004-024-01166-7 (PMC11549324; doi:10.1038/s42004-024-01166-7)
Supplement: Supplementary file 1 — Supplementary Information [file 42004_2024_1166_MOESM1_ESM.pdf]

Supporting Information: Machine Learning Insights into Predicting Biogas Separation in Metal Organic Frameworks

Isabel Cooley, Samuel Boobier, Jonathan D. Hirst and Elena Besley*

School of Chemistry, University of Nottingham, University Park, Nottingham, NG7 2RD, United Kingdom.

Contributing authors: Isabel.Cooley@nottingham.ac.uk; samuel.boobier@gmail.com; Jonathan.Hirst@nottingham.ac.uk; Elena.Besley@nottingham.ac.uk;

Supplementary Note 1 Dataset Preparation and Analysis

The curation workflow used to refine the dataset used for Grand Canonical Monte Carlo (GCMC) analysis and machine learning (ML) training and cross validation is outlined below. Initially, 6,768 solvent-evacuated MOFs were taken from the work of Glover and Besley. [1] Some structures contained either no metal or no carbon, so could not be considered as metal organic frameworks, while others contained only one or two elements. These problems may be the result of over-zealous solvent removal algorithms. [2] Meanwhile, some contained overlapping atoms or lacked H atoms, which may be due to problems in initial crystal structure determination. [3] All structures containing no metal, no carbon, or only one or two elements were first identified and removed from the dataset, leaving a total of 6,663 structures.

Overlapping atoms were next combated. Any structures with any atom-atom distances less than 0.5 Å were removed from the dataset. This is a reasonably lenient bond length criterion, as 0.5 Å is substantially shorter than the H_2 bond (0.74 Å). Therefore, any MOFs that are removed at this stage can be confidently considered to be problematic structures. Following application of this criterion, 6,638 structures remained.

The structural information files of some of the MOFs contained references to D atoms in place of H in some places. Computational force fields do not as a rule distinguish between isotopes. For simplicity, all D atoms were replaced with H. Since this did not involve removal of any MOFs from the database, 6,638 structures still remained. It was then possible to remove all structures which still contained no H atom, following which 6,359 structures remained.

In addition to this collection of observed symptoms of problematic structures, oxidation state counting was used to remove structures with unviable oxidation states using a published MOF Oxidation State And Electron Count (MOSAEC) code. [4] The code is designed to read a crystallographic information file (cif) of a MOF and identify metal atoms with any of a list of features which indicate a high chance of problematic oxidation states. The MOSAEC code was applied to all 6,359 remaining structures assuming a neutral framework and any that were flagged with any one of the problematic features were removed. A total of 3,554 structures were flagged as problematic, leaving only 3,086 MOFs in the dataset.

A further filter was for dimensionality: the raw database contained MOFs in 3D, 2D, and 1D, as well as 0D structures which were not periodic in any direction and so would be better described as molecules or metal complexes than as MOFs. The dimensionality of each of the 3,086 structures was determined algorithmically using the Zeo++ software package. [5] Of these, 1,715 3D structures, 686 2D structures, 611 1D structures and 74 0D structures were identified. Both 3D and 2D structures can constitute MOFs, and can have advanced and valuable properties, including in gas separation. 1D structures

may be considered to fit the definition of MOFs, but are likely to possess structural properties and exhibit behaviour that is different to more conventional MOFs, and 1D structures may arise from erroneous removal of connecting struts during solvent stripping. All 1D and 0D structures were removed from the database, leaving a total of 2,401 MOFs. Of these 2,401 MOFs, charge equilibration calculations necessary for the simulations were unable to complete for 20 structures, leaving a total of 2,381.

The inclusion of duplicate MOFs in databases can cause further problems in machine learning studies. They reduce diversity of training data and can lead to data leakage by inclusion of structures in test sets which are identical or highly similar to those in test sets. [6, 7] In the CSD, the refcode of a structure is an identifying string of 6 letters. The 6 letters may have a 2-digit number appended, and materials identified by the same string of 6 letters with different digits appended are based on the same MOF structure, but may differ by coordinate translations, inclusion of solvent, or similar, potentially leading to duplicates of similar structures. Indeed, the dataset of 2,381 MOFs contained several near-identical copies of the MOF Cu-BTC (refcode DOTSOV), which negatively affected the statistics of the dataset in initial model fitting. Similar repetition of MOF structures may also be seen in cases with entirely different refcodes. In these cases, duplication cannot be identified by simple comparison of names. [6] Therefore, a workflow based on comparison of structures was implemented to remove duplicate MOFs from among the 2,381 structures. This was based on similar workflows previously implemented in the literature [6, 7] and proceeded as follows.

First, an undecorated (agnostic to atom type) structural graph of each MOF in the training set was obtained using the Pymatgen materials analysis python library. [8] From each graph, a Weisfeiler-Lehman structure hash was generated using the NetworkX package. [9] Two identical Weisfeiler-Lehman hashes indicate structural graphs which are the same (although similarity of hashes short of an exact match gives no additional information). Therefore, for any groups of MOFs with identical hashes, only one MOF was retained in the dataset. Finally, to account for identifiable cases which may have been missed by the hash comparison, any remaining MOFs whose CSD refcode was based on a 6-letter string already represented in the dataset were also removed. The workflow may have removed some MOFs which differed substantially from their partners, but in general will have removed duplicates and improved the diversity of the database.

Following the curation procedure, 1,910 MOFs remained in the dataset. This is a small dataset compared to those used in some previous high-throughput and machine learning studies of MOFs, but it contains only structures which have been synthesised experimentally and have passed through a stringent filtration process for structural viability, relevance and deduplication. The procedure used to curate the training set is summarised in Figure S1. This includes summary of steps taken by Glover and Besley [1] prior to the current work as well as steps taken as part of this work.

A number of the issues addressed by the curation procedure in this work have been considered before, and therefore the selected methods may be compared to previous works. For example, the work of Velioglu and Keskin [10] addressed three common features of MOF databases: (i) the presence of residual solvent molecules, (ii) the absence of hydrogen atoms and (iii) the erroneous removal of charge balancing ions. In the case of solvent molecules, these are routinely removed from cif files as part of MOF studies, but the algorithm for removal may affect observed results. This work made use of cif files obtained from the screening of Glover and Besley, which had been stripped of solvent molecules using the script published alongside the CSD MOF subset by Moghadam et al. [11] Missing hydrogen atoms were dealt with in this work by removal of any cif which contained no hydrogen (following replacement of D atoms with H), as discussed above. Meanwhile, the possibility of missing charge balancing ions was accounted for as part of the application of the MOSAEC script [4] for oxidation state counting. At this stage, structures were only permitted to pass if reasonable oxidation states were assigned to their atoms assuming a neutral framework. Therefore, any framework for which vital charge balancing ions had been stripped would not pass this stage. In the case of both missing hydrogen atoms and missing charge balancing ions, structures were eliminated from the dataset; replacement of these missing components was not attempted.

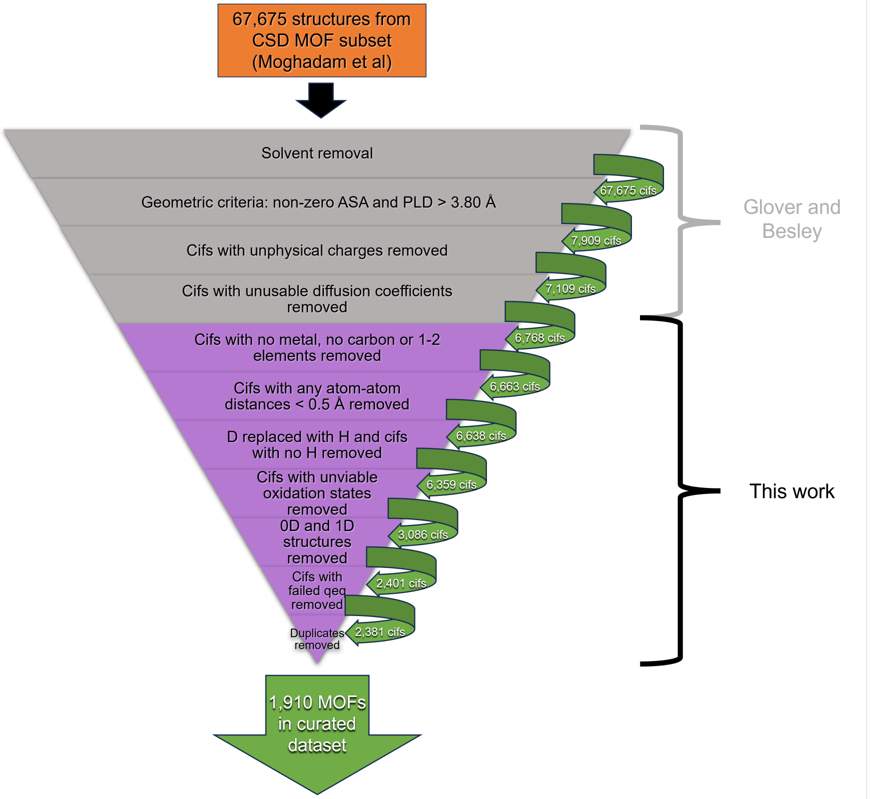

Fig. S1 A summary of the curation steps which have been applied to the CSD MOF subset [11] to generate the curated dataset used in this work. Steps taken by Glover and Besley [1] to produce the initial dataset used in this work are shown in grey and steps taken as part of this work are shown in purple. Removal of solvents by Glover and Besley [1] made use of the solvent removal algorithm published alongside the CSD MOF subset. [11]

To allow consideration of the diversity of the dataset, a bar chart giving the frequency of occurrence of different metal atoms among the 1,910 curated MOFs is shown in Figure S2. An external test set taken from the Northwestern hypothetical database [12] was also used to assess the transferability of trained models. This test set is discussed in detail below and in the main text. It underwent the same curation procedures as the training and validation set, with the addition of a check for any structures with Weisfeiler-Lehman hashes matching those in the training set, which found no matches. Beginning with 1,000 hypothetical MOFs taken at random from the database, the curation procedure yielded 330 total structures. The abundance of different metal atoms among this external test set is also illustrated in Figure S2.

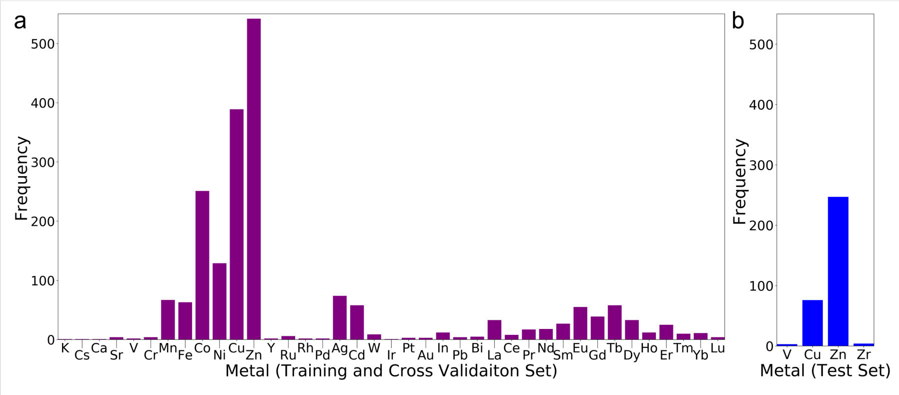

Fig. S2 Bar charts displaying the abundance of metal atoms in (a) the curated training set (1,910 total MOFs) and (b) the external test set used in this work (330 total MOFs).

A total of 39 different metals are represented in the training and cross-validation set. These occur with a range of different frequencies. The most abundant metal is Zn, which occurs in 542 MOFs, followed by Cu in 389 MOFs and Co in 251 MOFs. The metal centres in the external test set display less diversity. Only 4 different metals are represented and the dataset is heavily dominated by Zn (247 MOFs) and Cu (76 MOFs).

Table S1 Analysis of the target values predicted in this work

№	Descriptor	Minimum	Maximum	Mean	Median	Std Dev
1	BM CO2 loading (mol/kg)	3.62e-01	1.71e+01	6.05e+00	5.76e+00	3.00e+00
2	BM CH4 loading (mol/kg)	3.61e-04	3.61e+00	8.79e-01	6.52e-01	7.26e-01
3	SC CO2 loading (mol/kg)	5.14e-01	2.45e+01	8.04e+00	7.06e+00	4.57e+00
4	SC CH4 loading (mol/kg)	3.45e-01	1.04e+01	4.11e+00	4.01e+00	1.80e+00
5	TSN	5.22e-02	3.29e+01	5.87e+00	5.11e+00	3.99e+00
6	LOG_{10} TSN	-1.28e+00	1.52e+00	6.57e-01	7.08e-01	3.45e-01

Supplementary Note 2
Target Value Selection

Initially, five target values were considered: BM CO_2 loading (mol/kg); BM CH_4 loading (mol/kg); SC CO_2 loading (mol/kg); SC CH_4 loading (mol/kg); and TSN. Table S1 shows an analysis of the minimum, maximum, mean, and median of the target values. Figures S3 and S4 shows the distribution of the target values. It was noted that all target values were skewed to low values. This was especially the case for TSN, where the mean and median were 5.87 and 5.11 respectively, but the maximum value was at 32.9. To combat this skew, a log10 scale was applied to TSN, which centred the data.

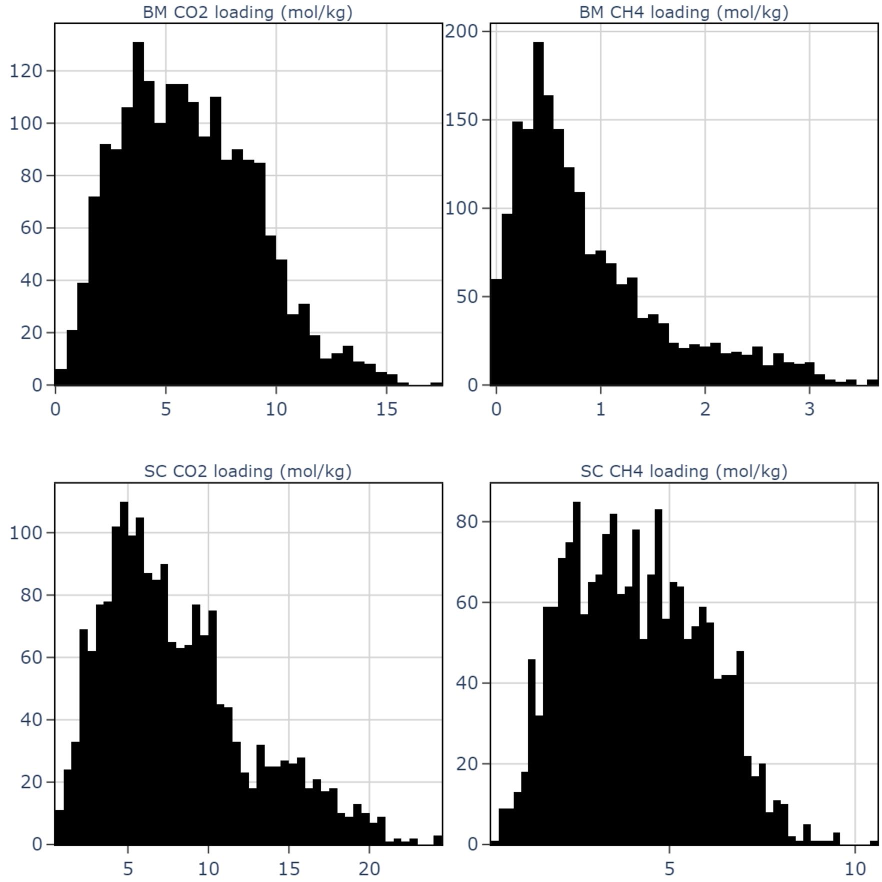

Fig. S3 Distribution of ranges for target values 1-4

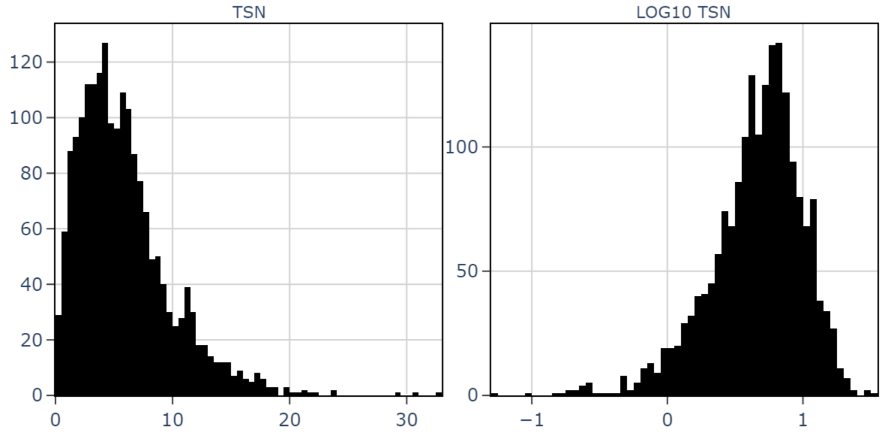

Fig. S4 Distribution of ranges for target values 5-6

2.1 The TSN Benchmark

For a binary classification model to make useful predictions relating to TSN, some benchmark value of TSN must be selected above which TSN is considered to be high and below which it is considered to be low. TSN metrics have been used in relation to related gas mixtures in previous literature, [13, 14] though they have not been widely applied and therefore a suitable benchmark value is not immediately obvious. As an example, $\text{TSN}_{H_2S+CO_2/C_1-C_3}$ was used as a metric in a screening of 606 hydrophobic MOFs for separation of H_2S and CO_2 from a natural gas mixture containing 7 parts CH_4, 1 part CO_2 and 0.2031 parts other gases by Qiao et al. [14] Hydrophobic MOFs were here defined as those for which $K_H(H_2O) < 2.6 \times 10^{-6}$, and were the focus due to their increased likelihood of water stability. Considering only hydrophobic MOFs, however, is likely to have limited the search to lower values of $\text{TSN}_{H_2S+CO_2/C_1-C_3}$ than otherwise. In this case the highest $\text{TSN}_{H_2S+CO_2/C_1-C_3}$ value calculated was 4.26 and a benchmark value of 2 mol kg^{-1} was used to identify potentially useful MOFs.

Assessment of the available literature relating to the limits of porous materials for the separation at hand shows that values of TSN_{CO_2/CH_4} notably higher than 2 mol kg^{-1} are achievable. A recent review of experimental studies of MOFs for CO_2 capture and separation [15] identified a wide range of MOFs that have been synthesised and applied to CO_2 uptake problems under various conditions in the past two decades. Over this large time period, various kinds of MOF were considered in the context of uptake and separation under various conditions of temperature and pressure, resulting in structures being deemed useful with CO_2 uptakes ranging from below 1 mol kg^{-1} [16, 17] to above 20 mol kg^{-1}. [18] Selectivity values are less accessible to experimental studies, but a handful were identified for selectivity of CO_2 over CH_4, also spanning a wide range, from below 10 to well over 100. Selectivity values are prone to

fluctuation and strong dependence on conditions, but where they are available in addition to raw uptakes, TSN may be calculated. Using the reported selectivity and uptake values, TSN_{CO_2/CH_4} upwards of 30 is observed. [18] In the context of the search undertaken here, which requires MOFs with high selectivity and uptake, these very high values of TSN_{CO_2/CH_4} are of particular interest, especially if they can be achieved under relevant conditions.

In line with the potential for selectivity and uptake values that yield high TSN, previous high-throughput screenings seeking related metrics have used benchmarks of $S_{CO_2/CH_4} = 5$ [19], $S_{CO_2/CH_4} = 10$ [19], CO_2 loading = 1 mol kg^{-1} (0.15 bar) [20], and CO_2 loading = 4 mol kg^{-1} (1 bar) [20] to define high-performing MOFs. The variation of benchmark values is in some cases down to the conditions at which the uptake processes are studied, and is sometimes down to the desired purpose of the model. A model with a higher threshold will classify fewer members of a dataset as high-performing and so reduce the number of structures on which more expensive calculations are carried out as part of a screening. However, models trained with higher benchmarks have been shown to identify a greater fraction of false positives, [19] and will also be more likely to miss high-performing structures.

Interestingly, a practically relevant zeolite currently used in natural gas scrubbing [19] to separate CO_2 and CH_4, zeolite 13X has a CO_2 uptake of around 6 mol kg^{-1} at 10 bar [21, 22] and its CO_2 selectivity in the presence of a CO_2/CH_4 1:9 gas mixture at 10 bar has been given as 14. This would correspond to a TSN of around 6.9 mol kg^{-1}, although selectivity and thus TSN are likely to be somewhat lower for an equal composition gas mixture.

When proposing new materials, it is productive to identify those with better performance than current benchmarks. With this in mind, a benchmark value of TSN = 5 mol kg^{-1} is used here. Using this value, it is expected that a high proportion of MOFs which a model predicts to have a strong performance will have values of TSN higher than that of materials used in current practice, without overly diluting the high-performing pool with low-performing MOFs. Additionally, all of those MOFs with very high TSN which may be of particular interest are expected to be classified as high performing. This threshold is also likely to identify materials with similar or better performance to those identified by benchmarks used for related values in recent high-throughput screenings for CO_2 uptake and selectivity. Using the higher of the thresholds taken from previous screenings, [19, 20], a MOF with CO_2 uptake of 4 mol $kg^{-}1$ and selectivity of 10 would possess a TSN of 4, though precise comparison is not possible due to the difference in the conditions used here and in previous works.

Examining the GCMC data in the context of the benchmark value of TSN = 5 mol kg^{-1} provides further confirmation for the prudence of the choice. In the first place, with a benchmark value of 5, 976 members of the curated MOF dataset are considered to be high-performing, leaving 934 low-performing MOFs. This is a useful spread of data: a machine learning model trained to classify MOFs into the two categories will be fed a sufficiently large volume of data

from each category to facilitate effective learning. This consideration is lent extra relevance here because of the relative sparsity of total data necessitated by the use of the curated MOF dataset. Meanwhile, if trained effectively and applied to an unseen dataset with a similar distribution of MOFs to the training set, such a model would be expected to identify around half of that dataset as potentially high-performing, allowing half of the structures to be rejected before any GCMC calculations are attempted and significantly improving the efficiency of a given screening. The well-performing curated MOFs identified by the TSN = 5 mol kg^{-1} threshold include a small proportion of hydrophobic MOFs (by the criteria used by Qiao et al. and elsewhere in the literature), [14] which may be particularly useful to identify. Additionally, initial predictions of TSN displayed statistical deterioration close to a value of 5 mol kg^{-1}, meaning use of a higher threshold may lead to less confidence in classification.

Supplementary Note 3 Descriptor Selection

3.1 Initial Descriptor Set

An initial set of 21 descriptors was chosen to represent important MOF properties. These descriptors originated from the work of Glover and Besley. The list of 21 descriptors, along with a brief description of each and information about how they were calculated, is given in Table S2, in which cyan shaded descriptors are those used in this work (see Table 2 of the main text).

The descriptors were analysed in Table S3 and the ranges were plotted in Figures S5 to S10. The distributions of several descriptors were found to be skewed, in particular permeability and Henry constant descriptors (P_CH4, P_CO0, P_H2S, K0_CH4, K0_CO2, K0_H20, and K0_H2S). PLD, LCD, and diffusion coefficient descriptors (DC_CH4, DC_CO0, and DC_H2S) were also skewed to a lesser extent.

Table S2 A complete set of 21 descriptors of Glover and Besley [1]; descriptors shown in cyan shaded were used to train the ML model in this work. Where relevant: MC = Monte Carlo, GCMC = grand canonical Monte Carlo, MD = molecular dynamics, r = probe radius.

Descriptor	Description	Method	Software
**PLD /Å**	**Diameter of the largest sphere that can percolate through the MOF**	**Voronoi network**	**Zeo++** [5]
**LCD /Å**	**Diameter of the largest sphere that fits inside the MOF**	**Voronoi network**	**Zeo++** [5]
**Density /g cm^{-3}**	**Mass of MOF per unit volume**		**Zeo++** [5]
**VSA /m^2 cm^{-3}**	**Surface area accessible to the centre of a probe (r = 1.86 Å) per unit volume**	**Voronoi network MC sampling**	**Zeo++** [5]
GSA /m^2 g^{-1}	Surface area accessible to the centre of a probe (r = 1.86 Å) per unit mass	Voronoi network MC sampling	Zeo++ [5]
**VF**	**Fraction of the volume not occupied by MOF atoms**	**Voronoi network MC sampling**	**Zeo++** [5]
PV /cm^3 g^{-1}	Volume accessible to a probe (r = 0 Å) per unit mass	Voronoi network MC sampling	Zeo++ [5]
$K_0(CH_4)$ /mol kg^{-1} Pa^{-1}	Henry constant of CH_4 in the MOF at infinite dilution	Force fields GCMC	RASPA [23]
$K_0(CO_2)$ /mol kg^{-1} Pa^{-1}	Henry constant of CO_2 in the MOF at infinite dilution	Force fields GCMC	RASPA [23]
$K_0(H_2S)$ /mol kg^{-1} Pa^{-1}	Henry constant of H_2S in the MOF at infinite dilution	Force fields GCMC	RASPA [23]
$K_0(H_2O)$ /mol kg^{-1} Pa^{-1}	Henry constant of H_2O in the MOF at infinite dilution	Force fields GCMC	RASPA [23]
**$Q_0^{st}(CH_4)$ /kJ mol−1**	**Heat of adsorption of CH_4 in the MOF at infinite dilution**	**Force fields GCMC**	**RASPA** [23]
**$Q_0^{st}(CO_2)$ /kJ mol−1**	**Heat of adsorption of CO_2 in the MOF at infinite dilution**	**Force fields GCMC**	**RASPA** [23]
**$Q_0^{st}(H_2S)$ /kJ mol−1**	**Heat of adsorption of H_2S in the MOF at infinite dilution**	**Force fields GCMC**	**RASPA** [23]
**$Q_0^{st}(H_2O)$ /kJ mol−1**	**Heat of adsorption of H_2O in the MOF at infinite dilution**	**Force fields GCMC**	**RASPA** [23]
$D_0^C(CH_4)$ /cm^2 s^{-1}	Diffusion coefficient of CH_4 in the MOF at infinite dilution	Force fields MD	RASPA [23]
$D_0^C(CO_2)$ /cm^2 s^{-1}	Diffusion coefficient of CO_2 in the MOF at infinite dilution	Force fields MD	RASPA [23]
$D_0^C(H_2S)$ /cm^2 s^{-1}	Diffusion coefficient of H_2S in the MOF at infinite dilution	Force fields MD	RASPA [23]
$D_0^C(H_2O)$ /cm^2 s^{-1}	Diffusion coefficient of H_2O in the MOF at infinite dilution	Force fields MD	RASPA [23]
$P_0(CH_4)$ /Barrer	Permeability of CH_4 in the MOF at infinite dilution	Force fields GCMC, MD	RASPA [23]
$P_0(CO_2)$ /Barrer	Permeability of CO_2 in the MOF at infinite dilution	Force fields GCMC, MD	RASPA [23]
$P_0(H_2S)$ /Barrer	Permeability of H_2S in the MOF at infinite dilution	Force fields GCMC, MD	RASPA [23]
$P_0(H_2O)$ /Barrer	Permeability of H_2O in the MOF at infinite dilution	Force fields GCMC, MD	RASPA [23]

Table S3 Analysis of the 21 initial descriptors considered in this work

№	Descriptor	Minimum	Maximum	Mean	Median	Std Dev
1	PLD	3.80e+00	2.31e+01	5.62e+00	4.89e+00	2.11e+00
2	LCD	4.21e+00	3.20e+01	7.55e+00	6.49e+00	3.37e+00
3	Density (g/cc)	2.07e-01	5.84e+00	1.18e+00	1.15e+00	4.26e-01
4	VSA (m2/cc)	7.33e+01	3.09e+03	1.22e+03	1.16e+03	5.88e+02
5	GSA (m2/g)	4.34e+01	6.23e+03	1.31e+03	9.62e+02	1.07e+03
6	VF	2.84e-01	9.13e-01	5.80e-01	5.67e-01	1.06e-01
7	PV (cc/g)	9.35e-02	4.40e+00	5.90e-01	4.91e-01	3.95e-01
8	K0_CH4	5.97e-07	4.88e-04	2.28e-05	1.53e-05	2.68e-05
9	K0_CO2	7.52e-07	1.15e+01	1.74e-02	1.27e-04	3.18e-01
10	K0_H2S	1.43e-06	3.00e+01	1.84e-02	3.61e-04	6.87e-01
11	K0_H2O	1.58e-07	5.68e+12	6.36e+09	2.42e-04	1.71e+11
12	DC_CH4	2.74e-06	5.20e-03	5.41e-04	4.01e-04	4.80e-04
13	DC_CO2	9.21e-07	1.19e-03	1.90e-04	1.51e-04	1.65e-04
14	DC_H2S	1.34e-07	2.16e-03	1.94e-04	1.37e-04	1.96e-04
15	P_CH4	9.51e+03	6.83e+08	4.20e+06	1.86e+06	2.09e+07
16	P_CO2	4.10e+04	9.19e+10	1.12e+08	6.40e+06	2.30e+09
17	P_H2S	3.04e+05	2.31e+11	1.77e+08	1.49e+07	5.29e+09
18	Qst_CH4	-3.20e+01	-7.80e+00	-1.91e+01	-1.91e+01	3.85e+00
19	Qst_CO2	-7.81e+01	-1.05e+01	-3.08e+01	-2.88e+01	9.41e+00
20	Qst_H2S	-7.71e+01	-1.15e+01	-3.08e+01	-3.04e+01	7.47e+00
21	Qst_H2O	-1.46e+02	-7.84e+00	-4.66e+01	-3.71e+01	3.11e+01

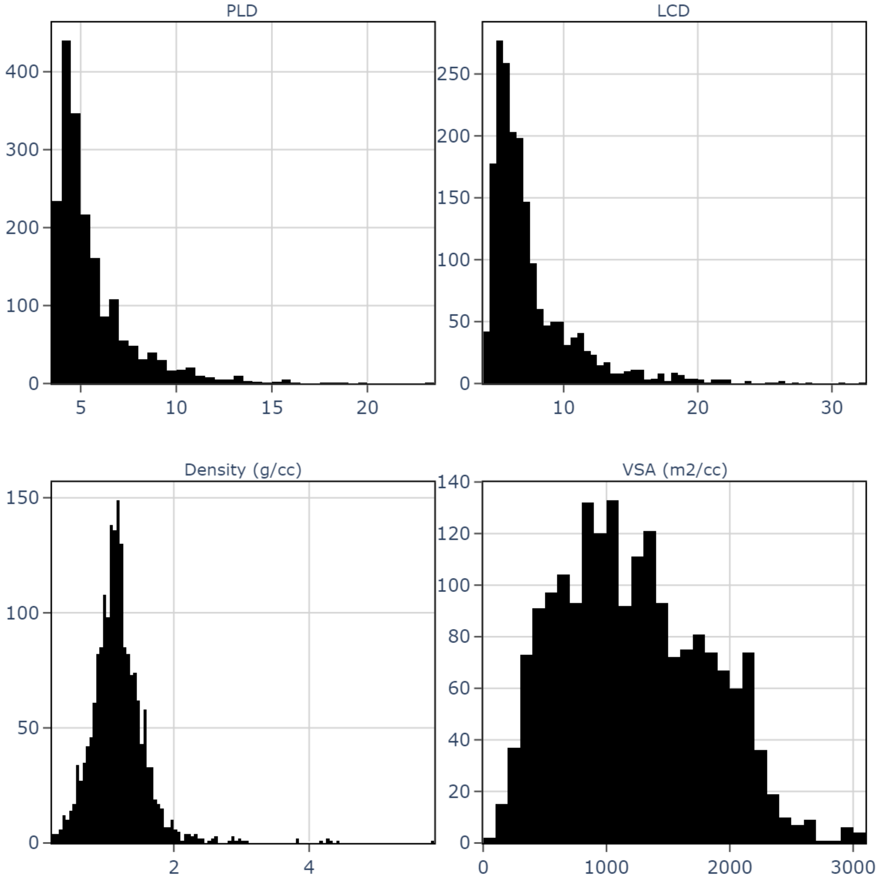

Fig. S5 Distribution of ranges for initial descriptors 1-4

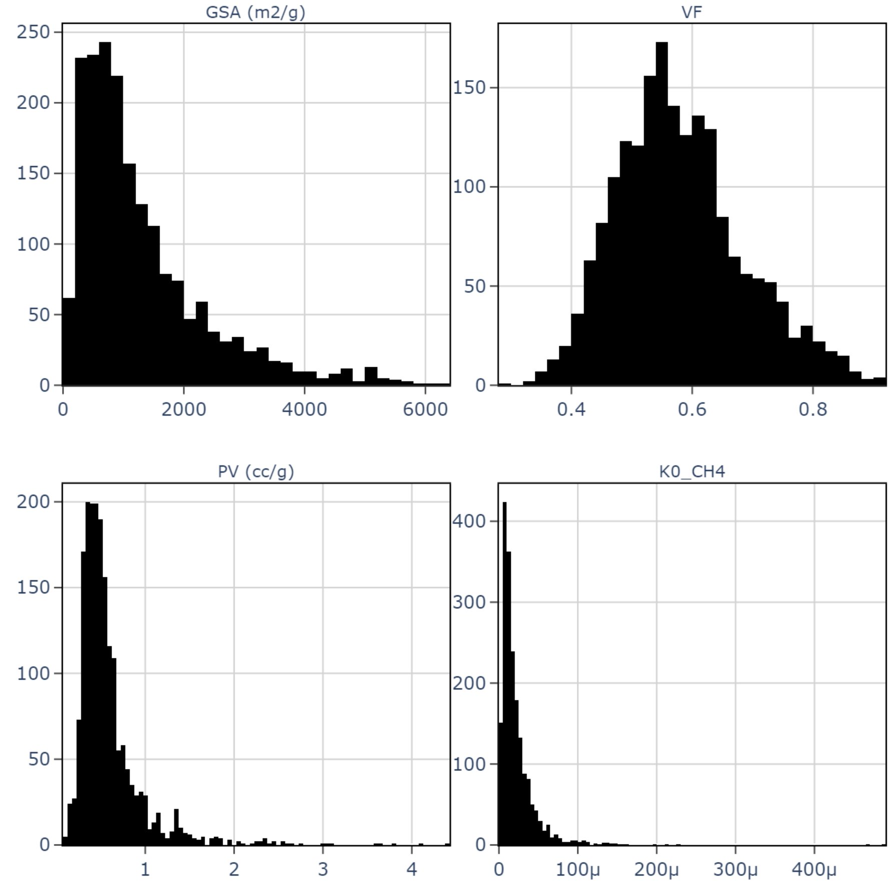

Fig. S6 Distribution of ranges for initial descriptors 5-8

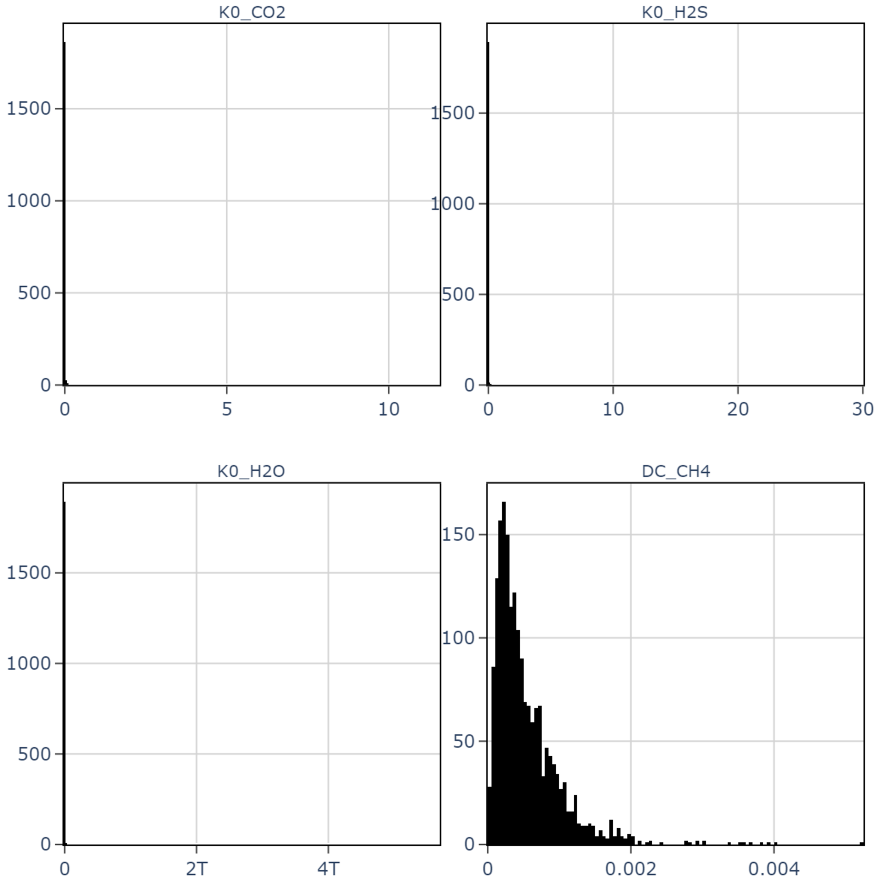

Fig. S7 Distribution of ranges for initial descriptors 9-12

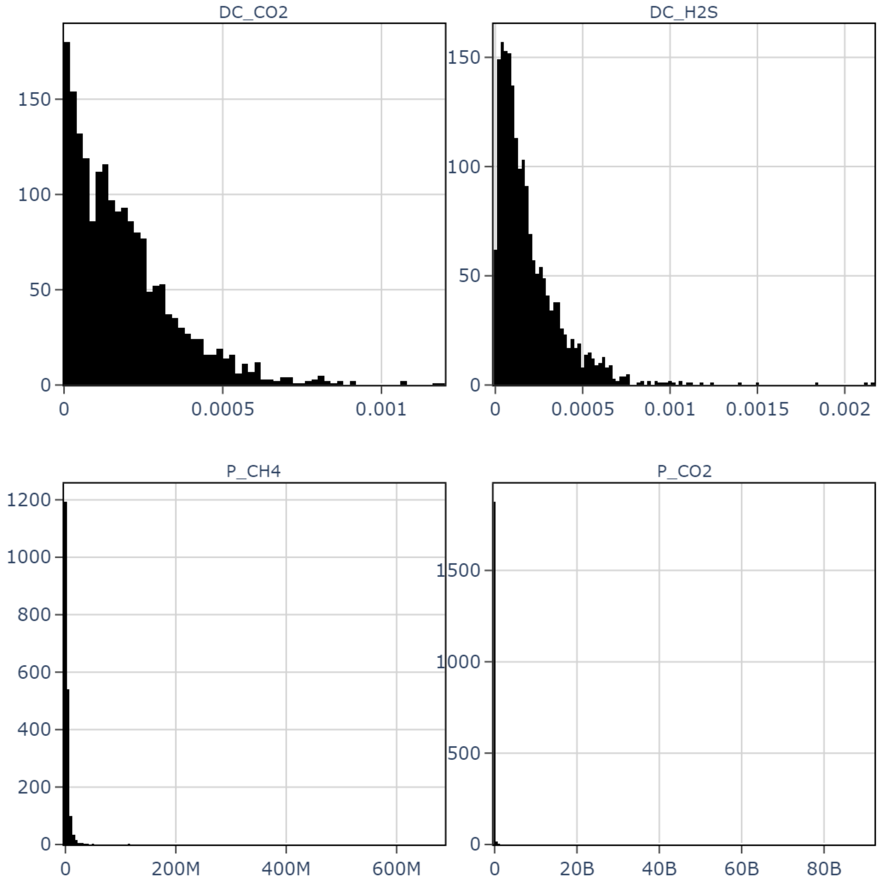

Fig. S8 Distribution of ranges for initial descriptors 13-16

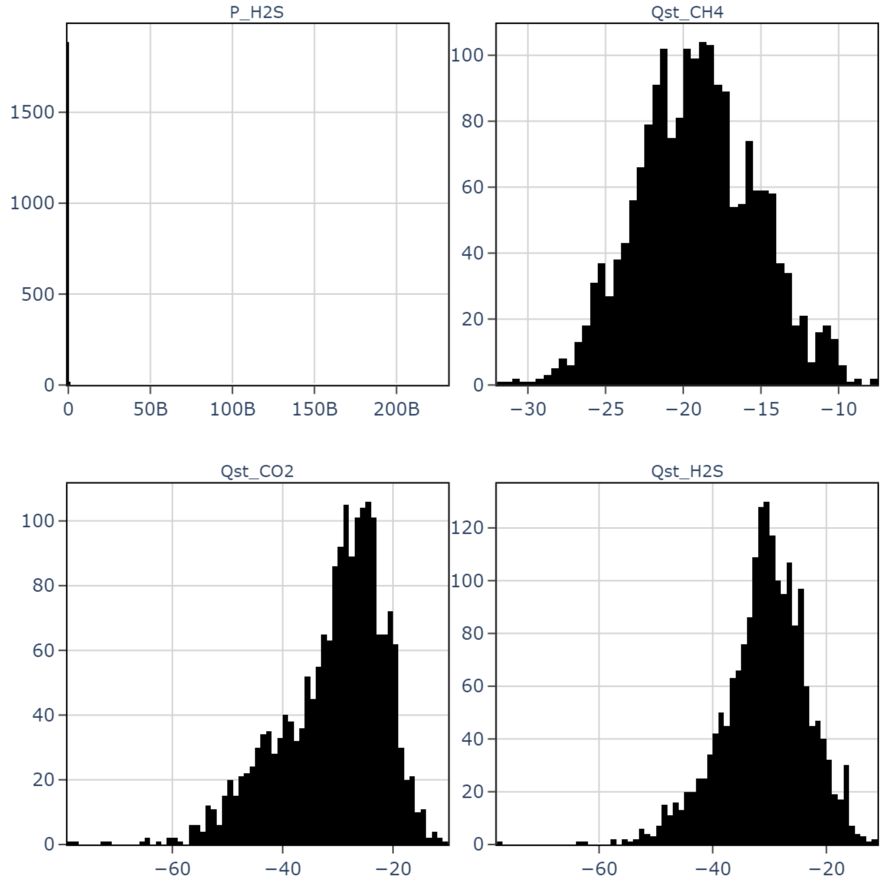

Fig. S9 Distribution of ranges for initial descriptors 17-20

Table S4 Analysis of log10 scaled descriptors

№	Descriptor	Minimum	Maximum	Mean	Median	Std Dev
1	PLD log10	5.80e-01	1.36e+00	7.28e-01	6.90e-01	1.30e-01
2	LCD log10	6.25e-01	1.51e+00	8.48e-01	8.12e-01	1.51e-01
3	PV (cc/g) log10	-1.03e+00	6.43e-01	-2.90e-01	-3.09e-01	2.17e-01
4	K0_CH4 log10	-6.22e+00	-3.31e+00	-4.81e+00	-4.82e+00	3.73e-01
5	K0_CO2 log10	-6.12e+00	1.06e+00	-3.72e+00	-3.90e+00	8.86e-01
6	K0_H2S log10	-5.84e+00	1.48e+00	-3.39e+00	-3.44e+00	7.45e-01
7	K0_H2O log10	-6.80e+00	1.28e+01	-1.80e+00	-3.62e+00	4.19e+00
8	DC_CH4 log10	-5.56e+00	-2.28e+00	-3.41e+00	-3.40e+00	3.74e-01
9	DC_CO2 log10	-6.04e+00	-2.92e+00	-3.94e+00	-3.82e+00	5.23e-01
10	DC_H2S log10	-6.87e+00	-2.67e+00	-3.93e+00	-3.86e+00	5.04e-01
11	P_CH4 log10	3.98e+00	8.83e+00	6.30e+00	6.27e+00	4.38e-01
12	P_CO2 log10	4.61e+00	1.10e+01	6.86e+00	6.81e+00	6.19e-01
13	P_H2S log10	5.48e+00	1.14e+01	7.20e+00	7.17e+00	6.12e-01

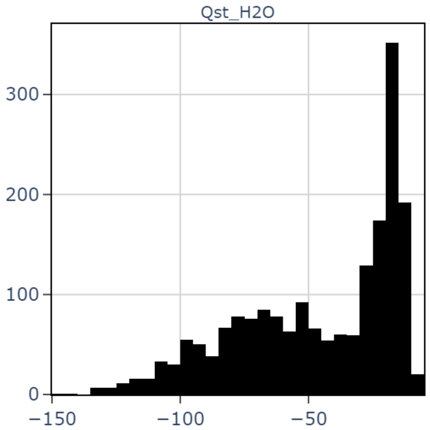

Fig. S10 Distribution of ranges for initial descriptor 21

3.2 Rescaling Descriptors

The skewed descriptors were scaled by log10. Analysis and distribution of these log10 scaled descriptors are shown in Table S4 and Figures S11 to S14. These new descriptors were less skewed than the original descriptors and were retained for the machine learning models.

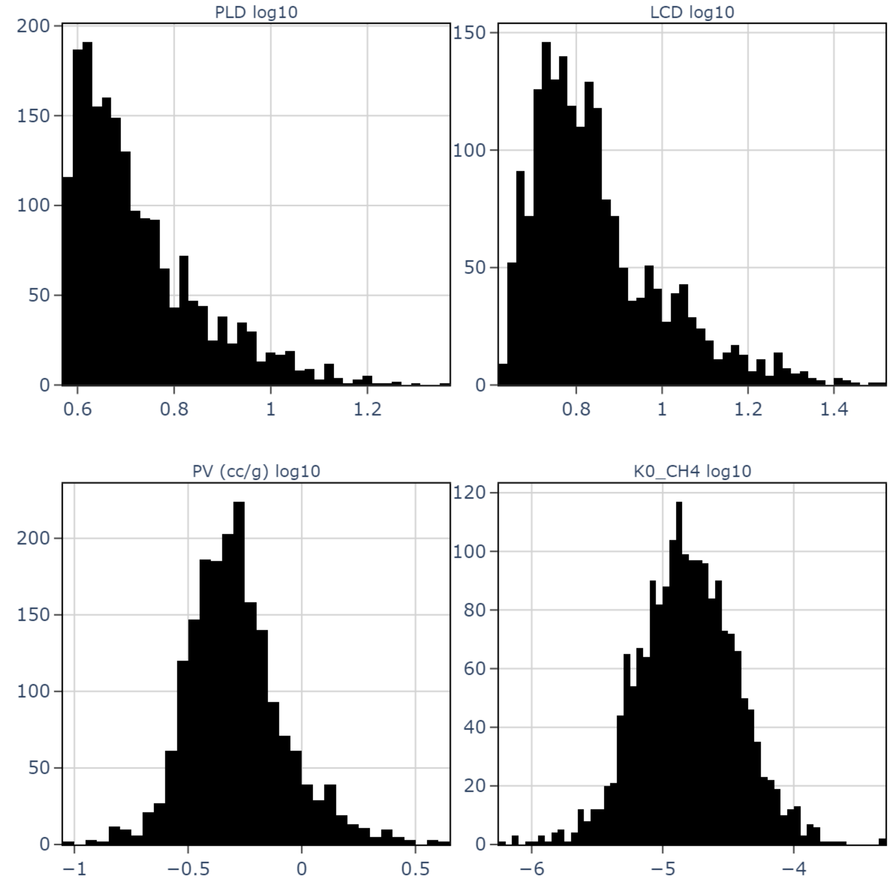

Fig. S11 Distribution of ranges for log10 scaled descriptors 1-4

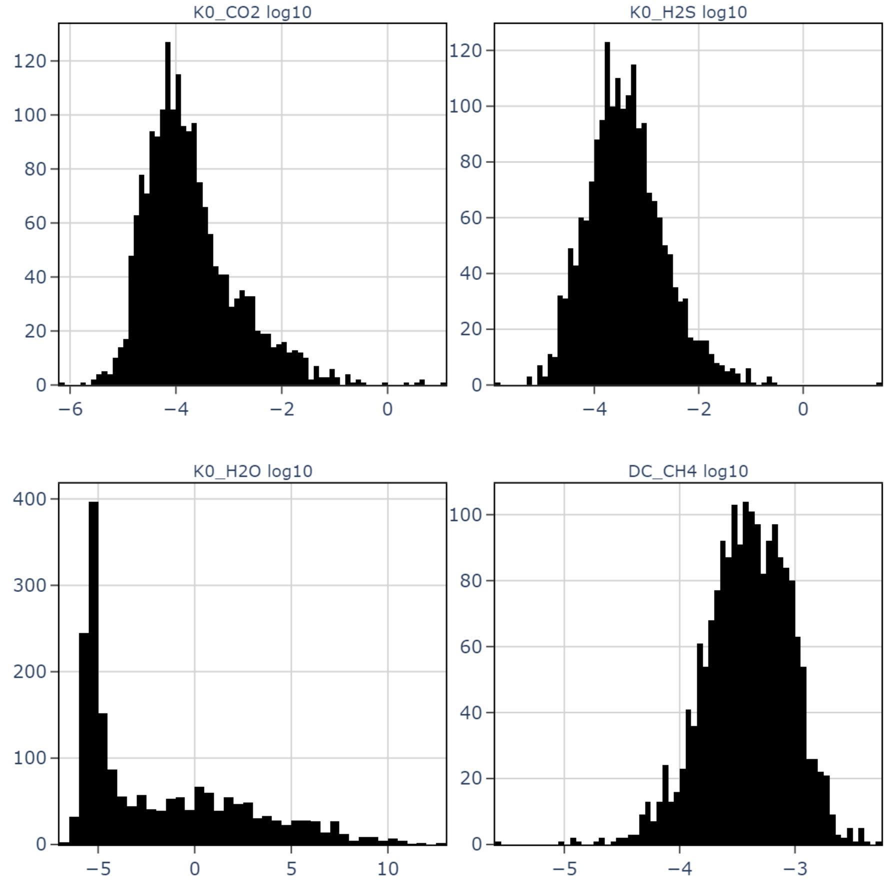

Fig. S12 Distribution of ranges for log10 scaled descriptors 5-8

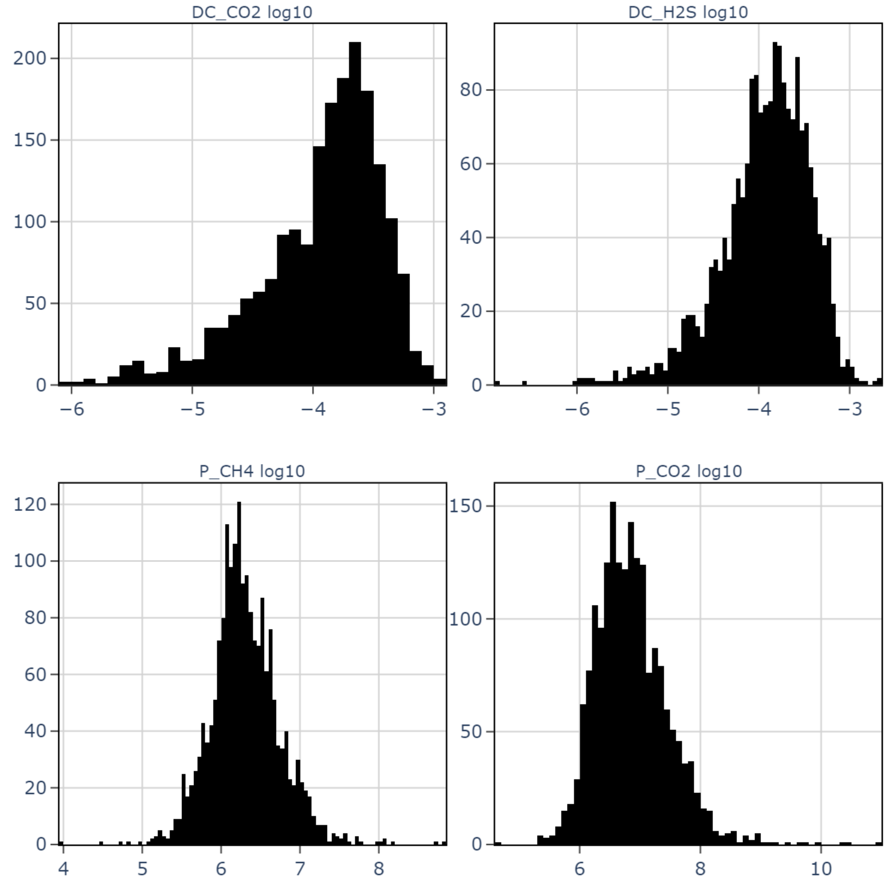

Fig. S13 Distribution of ranges for log10 scaled descriptors 9-12

Table S5 Selected top correlated descriptors

Descriptor 1	Descriptor 2	R^2
Qst_H2O	K0_H2O log10	0.9755
Qst_CO2	K0_CO2 log10	0.9070
Qst_H2S	K0_H2S log10	0.8791
GSA (m2/g)	PV (cc/g) log10	0.8562
VF	PV (cc/g) log10	0.8299
GSA (m2/g)	VF	0.8218
Qst_CH4	K0_CH4 log10	0.6232

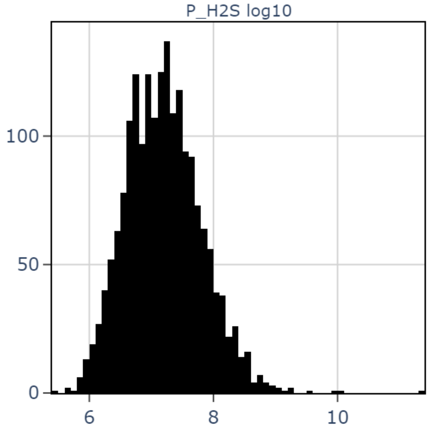

Fig. S14 Distribution of ranges for log10 scaled descriptor 13

3.3 Correlation Analysis

Descriptors were compared pairwise to check and removed correlated features. Pearson's R^2 was calculated with pandas corr() function and a heat map was produced to visualise the results. Selected top correlations are shown in Table S5 and the heat map is shown in Figure S15.

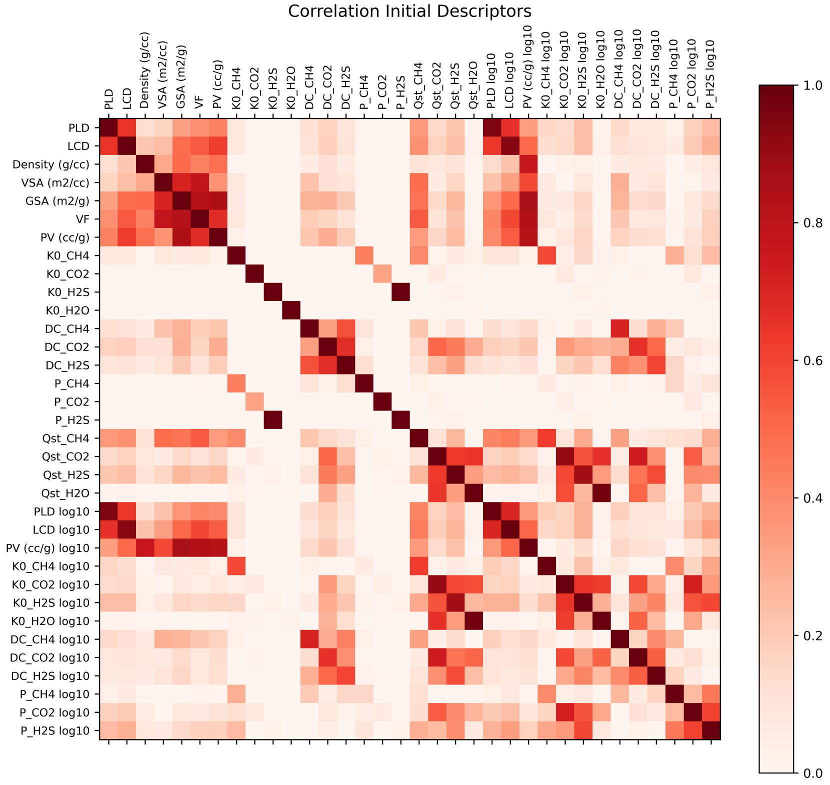

Fig. S15 Heat map of the correlation of descriptors considered in this work

It was noted that geometric features tended to be correlated. In particular void fraction (VF) and pore volume (PV (cc/g)) and VF and gravimetric surface area (GSA (m2/g)). In addition, Henry constant descriptors (K0_CO2, K0_H20, and K0_H2S) tended to correlate with heat of adsorption descriptors (Qst_CO2, Qst_H20, and Qst_H2S). This was seen to a lesser extent between K0_CH4 and Qst_CH4. Plots of these correlated descriptors are shown in Figures S18 to S21.

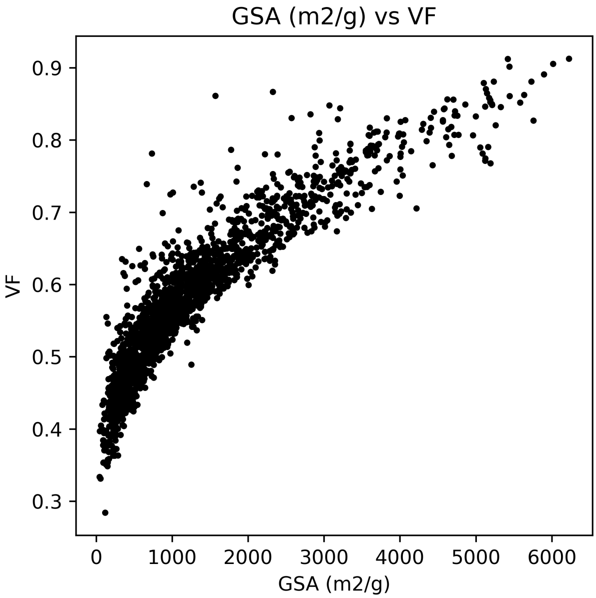

Fig. S16 Correlation between void fraction and gravimetric surface area

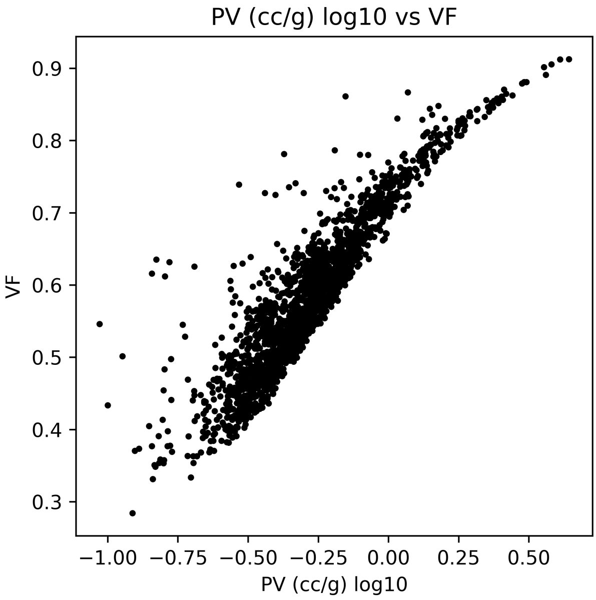

Fig. S17 Correlation between void fraction and pore volume

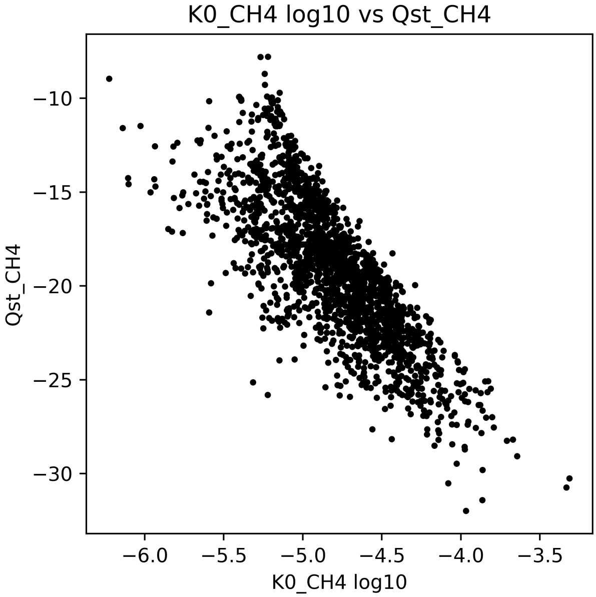

Fig. S18 Correlation between Henry constant and heat of absorption for CH_4

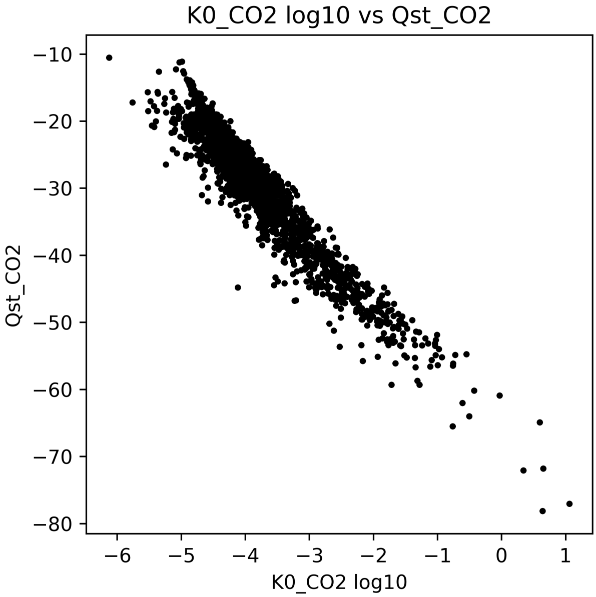

Fig. S19 Correlation between Henry constant and heat of absorption for CO_2

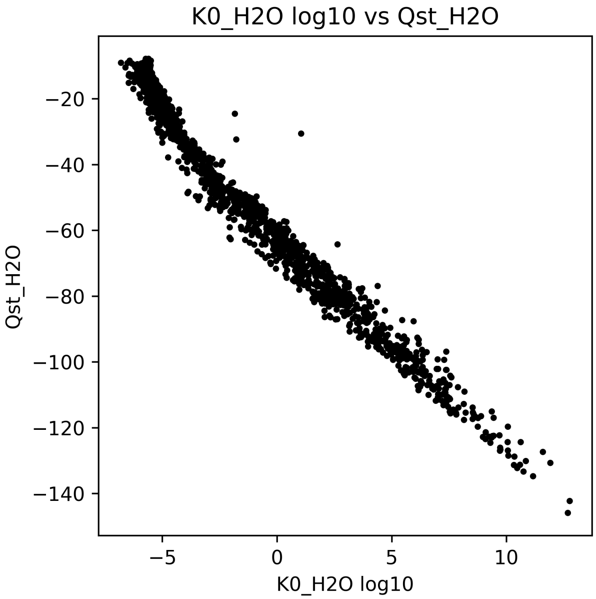

Fig. S20 Correlation between Henry constant and heat of absorption for H_2O

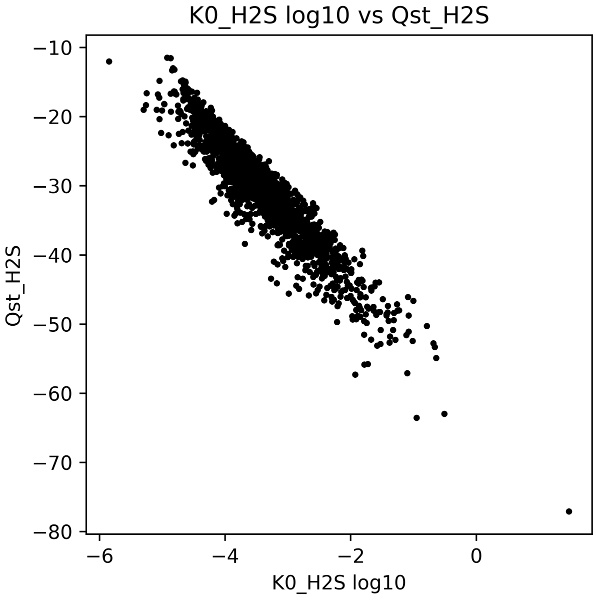

Fig. S21 Correlation between Henry constant and heat of absorption for H_2S

Table S6 Final set of 9 descriptors brought forward to machine learning

№	Descriptor	Minimum	Maximum	Mean	Median	Std Dev
1	PLD log10	5.80e-01	1.36e+00	7.28e-01	6.90e-01	1.30e-01
2	LCD log10	6.25e-01	1.51e+00	8.48e-01	8.12e-01	1.51e-01
3	Density (g/cc)	2.07e-01	5.84e+00	1.18e+00	1.15e+00	4.26e-01
4	VSA (m2/cc)	7.33e+01	3.09e+03	1.22e+03	1.16e+03	5.88e+02
5	VF	2.84e-01	9.13e-01	5.80e-01	5.67e-01	1.06e-01
6	Qst_CH4	-3.20e+01	-7.80e+00	-1.91e+01	-1.91e+01	3.85e+00
7	Qst_CO2	-7.81e+01	-1.05e+01	-3.08e+01	-2.88e+01	9.41e+00
8	Qst_H2S	-7.71e+01	-1.15e+01	-3.08e+01	-3.04e+01	7.47e+00
9	Qst_H2O	-1.46e+02	-7.84e+00	-4.66e+01	-3.71e+01	3.11e+01

3.4 Final Descriptor Set

From an initial set of 21, five descriptors had a log10 scale applied to improve their distributions. Pore volume, gravimetric surface area and Henry constant descriptors were removed due to their similarity to other descriptors found by correlation analysis. In addition, permeability and diffusion coefficient descriptors were discarded as they were deemed less relevant to the target values under consideration. Table S6 shows the final 9 descriptors brought forward as inputs for machine learning models.

Supplementary Note 4 Machine Learning Workflow

Figure S22 shows the machine learning protocol used in this study. The following sections show plots for the full results of 10-fold cross validation and unseen test set predictions.

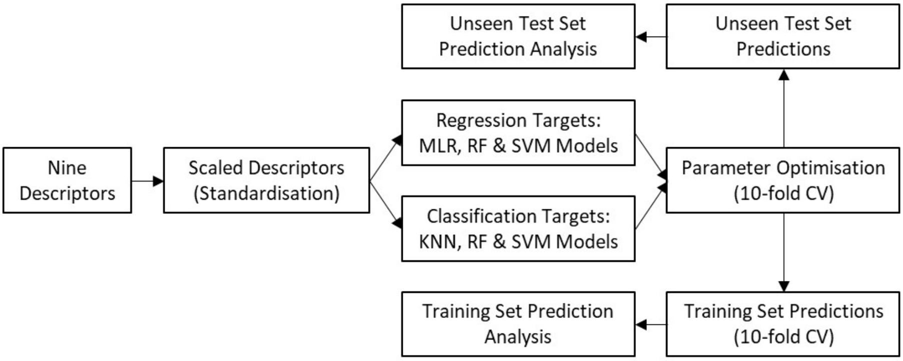

Fig. S22 Machine Learning workflow for generating and testing models

4.1 Machine Learning Metrics

4.1.1 Regression Metrics

R^2 and mean absolute error were used as metrics. R^2 was defined as the coefficient of determination, also called r2_score in *scikit-learn*. The equation for R^2 is shown in Equation 1, where n is the number of samples, $\hat{y}_i$ is the predicted value for the corresponding true value y_i, and $\bar{y}$ is the mean of the true values. An examination of the equation reveals that R^2 can be negative if the summed absolute error is larger than the summed difference to the mean. It follows that R^2=1 is prefect correlation and an R^2 close to zero denotes little predictive power.

$$R^2(y,\hat{y}) = 1 - \frac{\sum_{i=1}^{n}(y_i - \hat{y}_i)^2}{\sum_{i=1}^{n}(y_i - \bar{y})^2} \tag{1}$$

The mean absolute error is defined using the standard formulation in Equation 2.

$$MAE(y,\hat{y}) = \frac{\sum_{i=1}^{n}|y_i - \hat{y}_i|}{n} \tag{2}$$

4.1.2 Classification Metrics

Models were assessed using the standard measures of precision, recall, F1 score, and accuracy, as defined in Equations 3 to 6. We assume the reader is familiar with the confusion matrix and the definition of true positive (TP), true negative (TN), false positive (FP), and false negative (FN). Precision measure the rate of correct positive predictions and recall measures the rate of correctly predicting positive classes. F1 Score is the harmonic mean of precision and recall. Accuracy is the overall rate of correct predictions.

$$Precision = \frac{TP}{TP + FP} \tag{3}$$

$$Recall = \frac{TP}{TP + FN} \tag{4}$$

$$F1Score = 2 \times \frac{Precision \times Recall}{Precision + Recall} \tag{5}$$

$$Accuracy = \frac{TP + TN}{TP + TN + FP + FN} \tag{6}$$

In addition, the Brier score was computed, shown in Equation 7. This is a measure of the difference between the probability of the label, p_i, (in this case HIGH) and the actual outcome, o_i (set at 1 for positive, or HIGH, and 0 for negative or LOW). n is the number of samples.

$$BrierScore = \frac{\sum_{i=1}^{n}(p_i - o_i)^2}{n} \tag{7}$$

Lastly the receiver operating characteristic (ROC) curves were plotted and the resultant areas under the curve (AUC) were calculated. The ROC shows the predictive ability of the model as the discrimination threshold is varied. It is a plot of false positive rate against true positive rate. A value of 0.5 is comparable to random assignment and a value of 1 denotes perfect predictions.

Supplementary Note 5 Machine Learning Results

5.1 Regression Plots in Full

Regression models were built using Multiple Linear Regression (MLR), Random Forest (RF) and Support Vector Machine (SVM) for six targets. The models were assessed with 10-fold cross validation, using nested cross validation (5-fold) to optimise model parameters. Plots of predictions using each machine learning method for each target can be seen in Figures S23 to S25. The corresponding plots of error against target range can be seen in Figures S26 to S28.

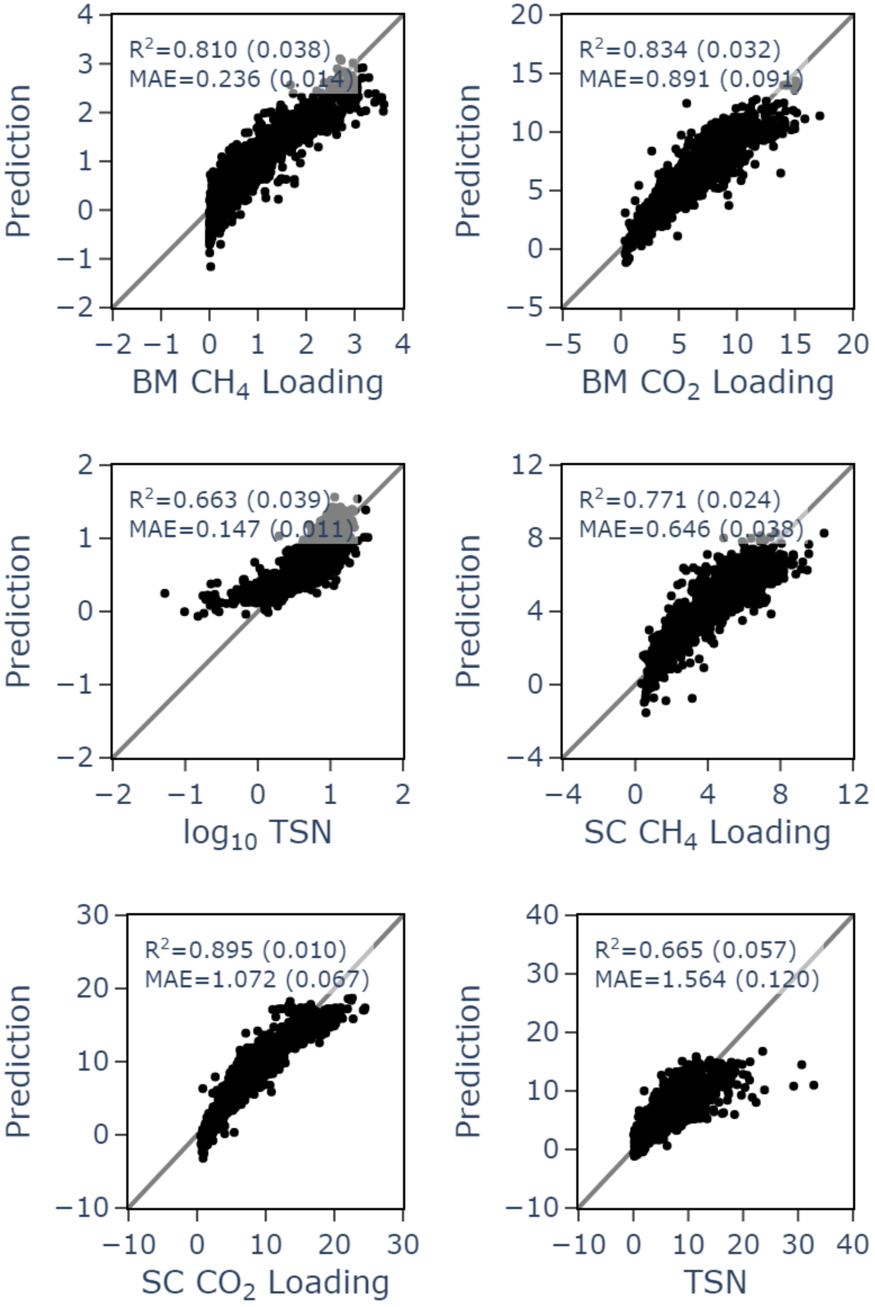

Fig. S23 Full regression predictions for MLR. All loading and TSN values in mol kg^{-1}.

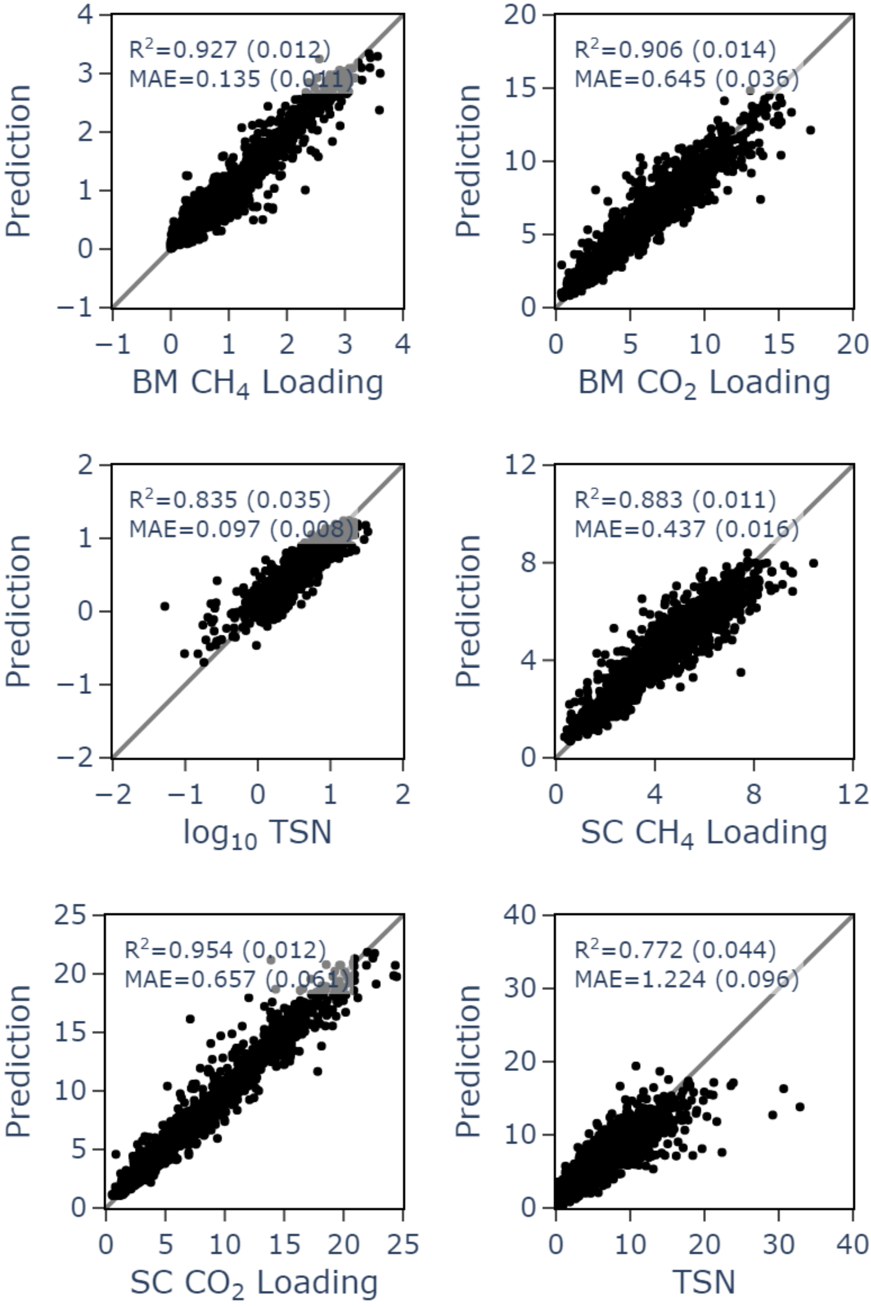

Fig. S24 Full regression predictions for RF

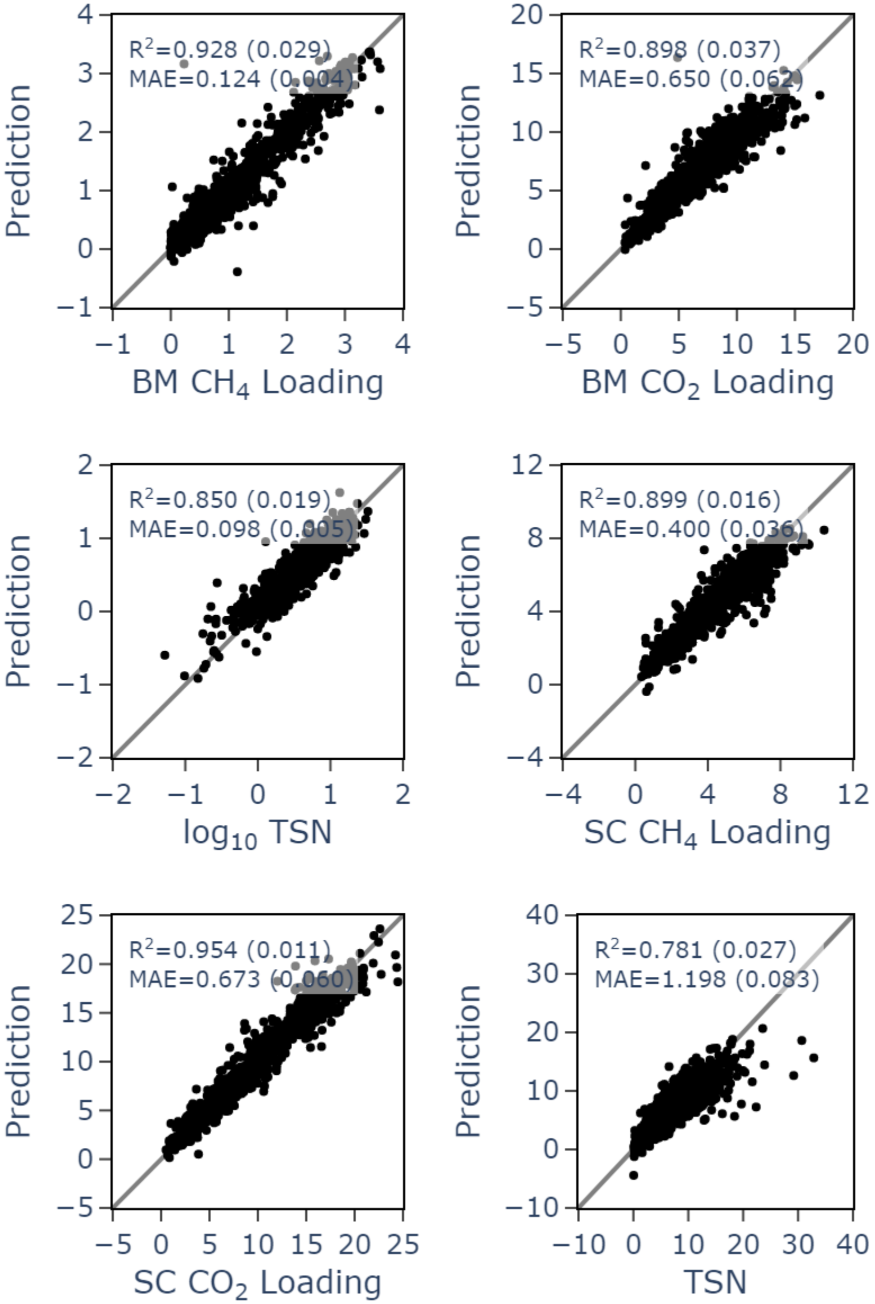

Fig. S25 Full regression predictions for SVM. All loading and TSN values in mol kg^{-1}.

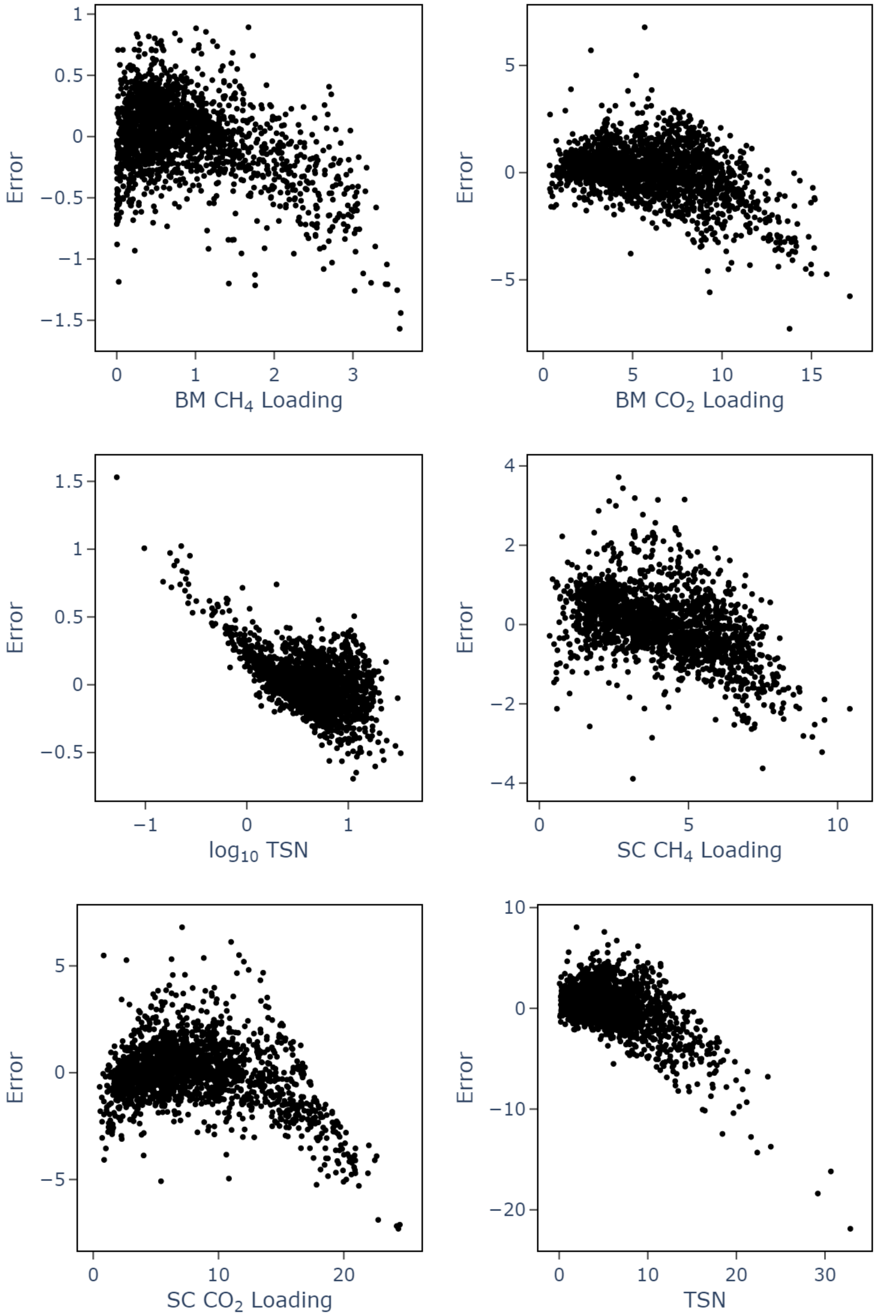

Fig. S26 Full error against target range for MLR

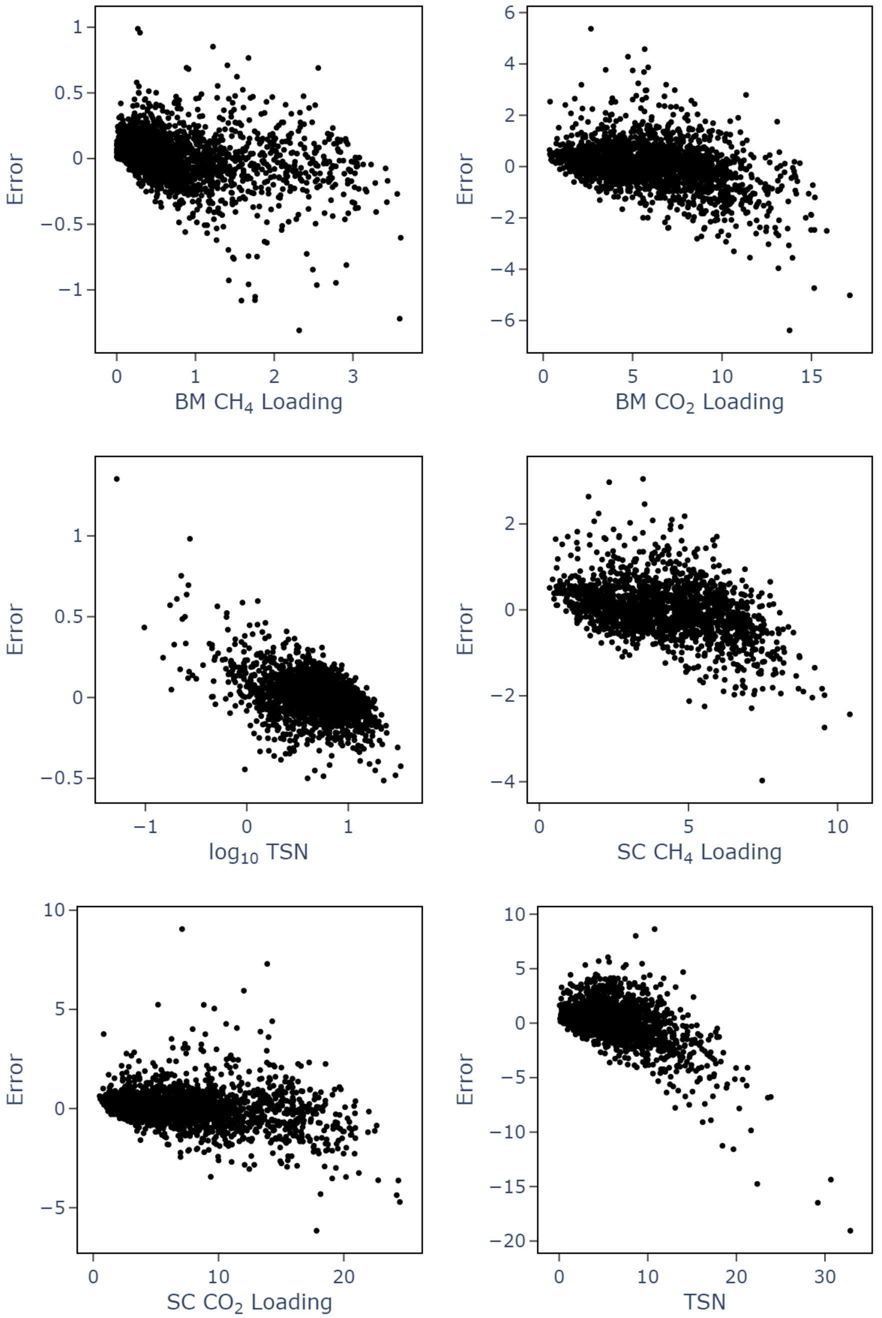

Fig. S27 Full error against target range for RF. All loading and TSN values in mol kg^{-1}.

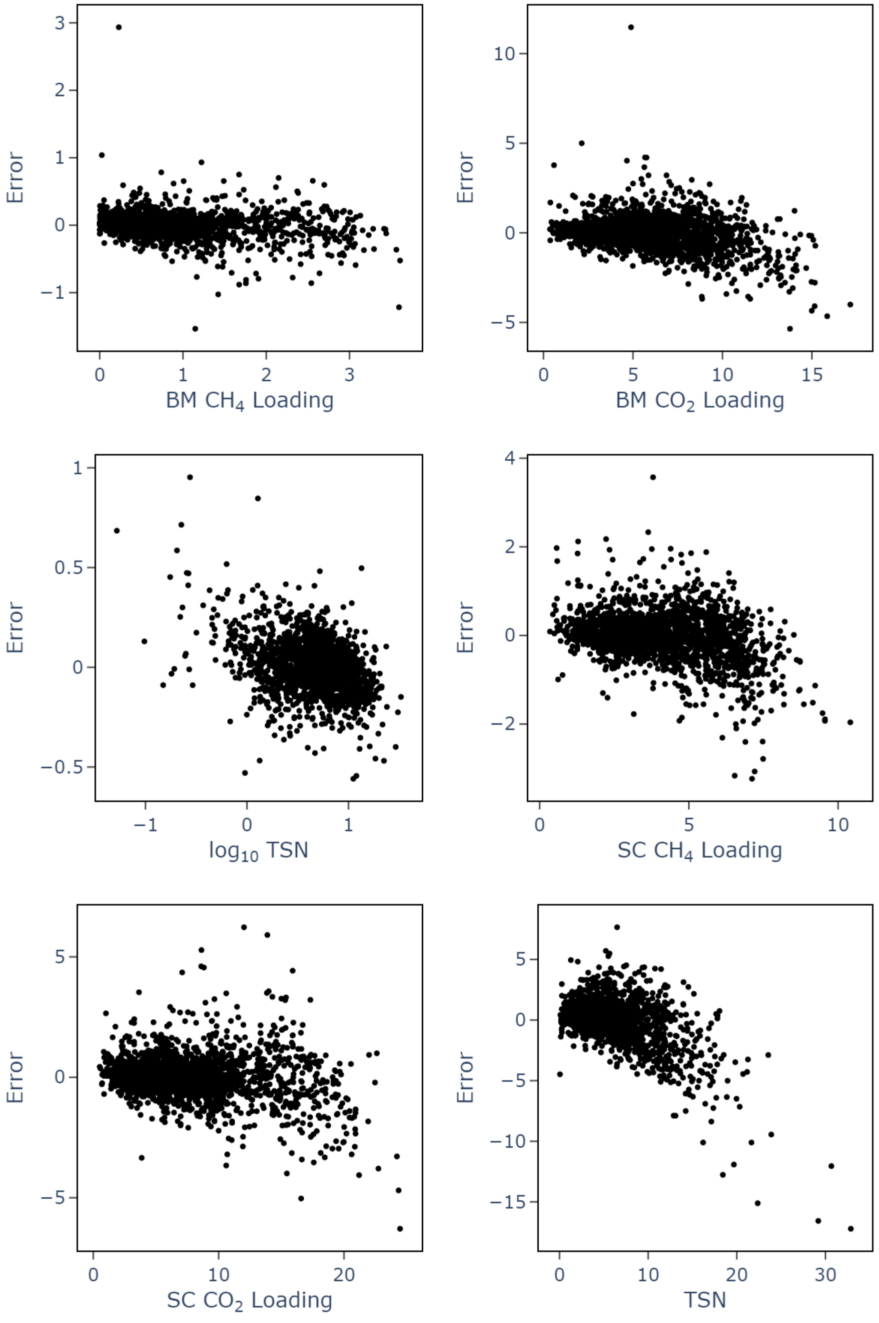

Fig. S28 Full error against target range for SVM. All loading and TSN values in mol kg^{-1}.

5.2 Classification Plots in Full

Classification models were built for TSN Class, where LOW = TSN $<$ 5 and HIGH = TSN $>$ 5. The machine learning methods k-Nearest Neighbours (KNN), RF and SVM were used to build the classification models, which was assessed with 10-fold cross validation. The receiver operator characteristic curve (ROC) was plotted for the resultant predictions, as shown in Figure S29. In addition, a histogram of the TSN values of each predicted class was plotted. Finally, a histogram of HIGH class probability for each class was generated. These can be seen in Figure S30.

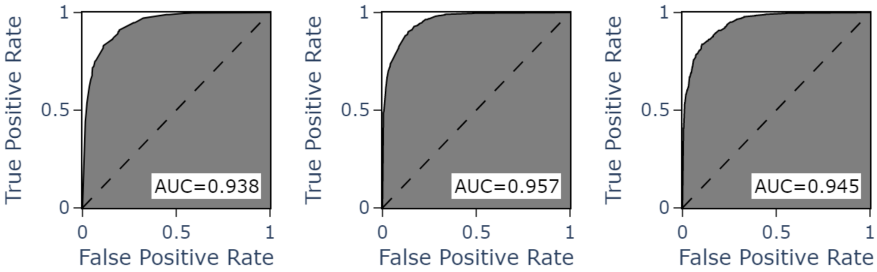

Fig. S29 Receiver operating characteristic curves for 10-fold cross validation results: KNN (left); RF (middle); SVM (right)

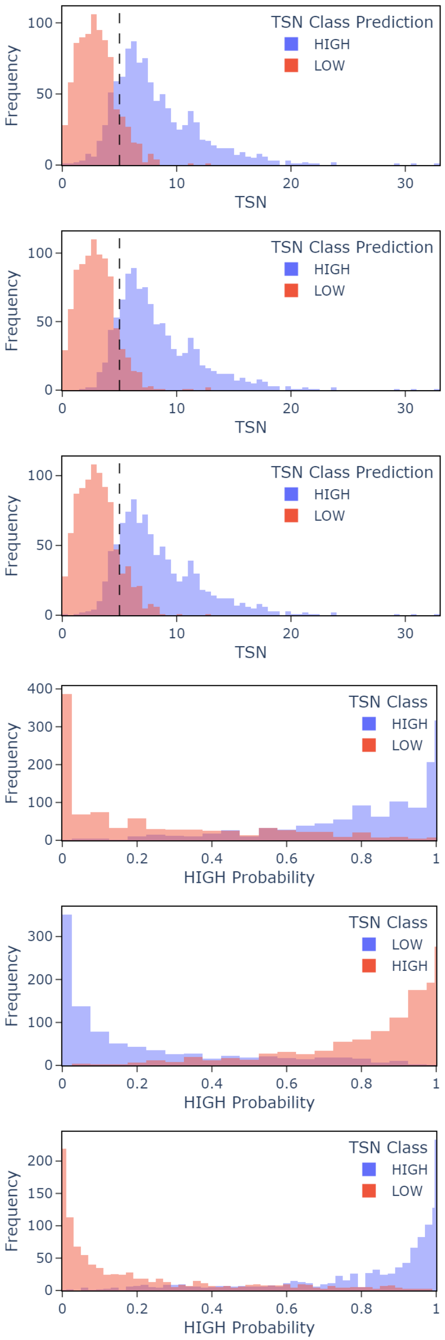

Fig. S30 Top half: Histogram of the TSN values of each predicted class for 10-fold cross validation results: KNN (top); RF (middle); SVM (bottom). Bottom half: Histogram of HIGH class probability for each class: KNN (top); RF (middle); SVM (bottom).

5.3 Regression and Classification Metrics in Full

The full metrics for regression and classification models can be seen in Table S7.

Table S7 Metrics for 10-fold cross validation results. The metrics are the average across 10 folds and the error is the standard deviation. MAE is in mol kg^{-1}, except for LOG10 TSN, for which MAE is in $\log_{10}$(mol kg^{-1}) units.

	Regression					
No	Target	Method	R^2	Error	MAE	Error
1	TSN	MLR	0.665	0.057	1.564	0.120
2	LOG10 TSN	MLR	0.663	0.039	0.147	0.011
3	SC CO_2 loading	MLR	0.895	0.010	1.072	0.067
4	BM CO_2 loading	MLR	0.834	0.032	0.891	0.091
5	SC CH_4 loading	MLR	0.771	0.024	0.646	0.038
6	BM CH_4 loading	MLR	0.810	0.038	0.236	0.014
7	TSN	SVM	0.781	0.027	1.198	0.083
8	LOG10 TSN	SVM	0.850	0.019	0.098	0.005
9	SC CO_2 loading	SVM	0.954	0.011	0.673	0.060
10	BM CO_2 loading	SVM	0.898	0.037	0.650	0.062
11	SC CH_4 loading	SVM	0.899	0.016	0.400	0.036
12	BM CH_4 loading	SVM	0.928	0.029	0.124	0.004
13	TSN	RF	0.772	0.044	1.224	0.096
14	LOG10 TSN	RF	0.835	0.035	0.097	0.008
15	SC CO_2 loading	RF	0.954	0.012	0.657	0.061
16	BM CO_2 loading	RF	0.906	0.014	0.645	0.036
17	SC CH_4 loading	RF	0.883	0.011	0.437	0.016
18	BM CH_4 loading	RF	0.927	0.012	0.135	0.011
	Classification					
	Metric	Method	HIGH	Error	LOW	Error
19	Precision	kNN	0.831	0.028	0.875	0.040
20	Recall	kNN	0.890	0.029	0.811	0.033
21	F1 Score	kNN	0.859	0.023	0.841	0.031
			Value	Error		
22	Accuracy	kNN	0.851	0.025		
23	Briers	kNN	0.099	0.013		
24	AUC	kNN	0.938			
			HIGH	Error	LOW	Error
25	Precision	SVM	0.856	0.036	0.863	0.038
26	Recall	SVM	0.868	0.045	0.849	0.033
27	F1 Score	SVM	0.861	0.029	0.855	0.019
			Value	Error		
28	Accuracy	SVM	0.859	0.022		
29	Briers	SVM	0.095	0.008		
30	AUC	SVM	0.945			
			HIGH	Error	LOW	Error
31	Precision	RF	0.866	0.034	0.898	0.028
32	Recall	RF	0.904	0.037	0.856	0.026
33	F1 Score	RF	0.884	0.033	0.876	0.022
			Value	Error		
34	Accuracy	RF	0.881	0.027		
35	Briers	RF	0.083	0.012		
36	AUC	RF	0.957			

5.4 Leave one out descriptor plots

For the regression targets, random forest models were rebuilt with 10-fold cross validation leaving out each descriptor in turn. This was to further assess the importance of each descriptor to the model. The value shown is the mean metric across the 10 folds and the error is the standard deviation across the 10 folds. This is shown in Figures S31 and S32.

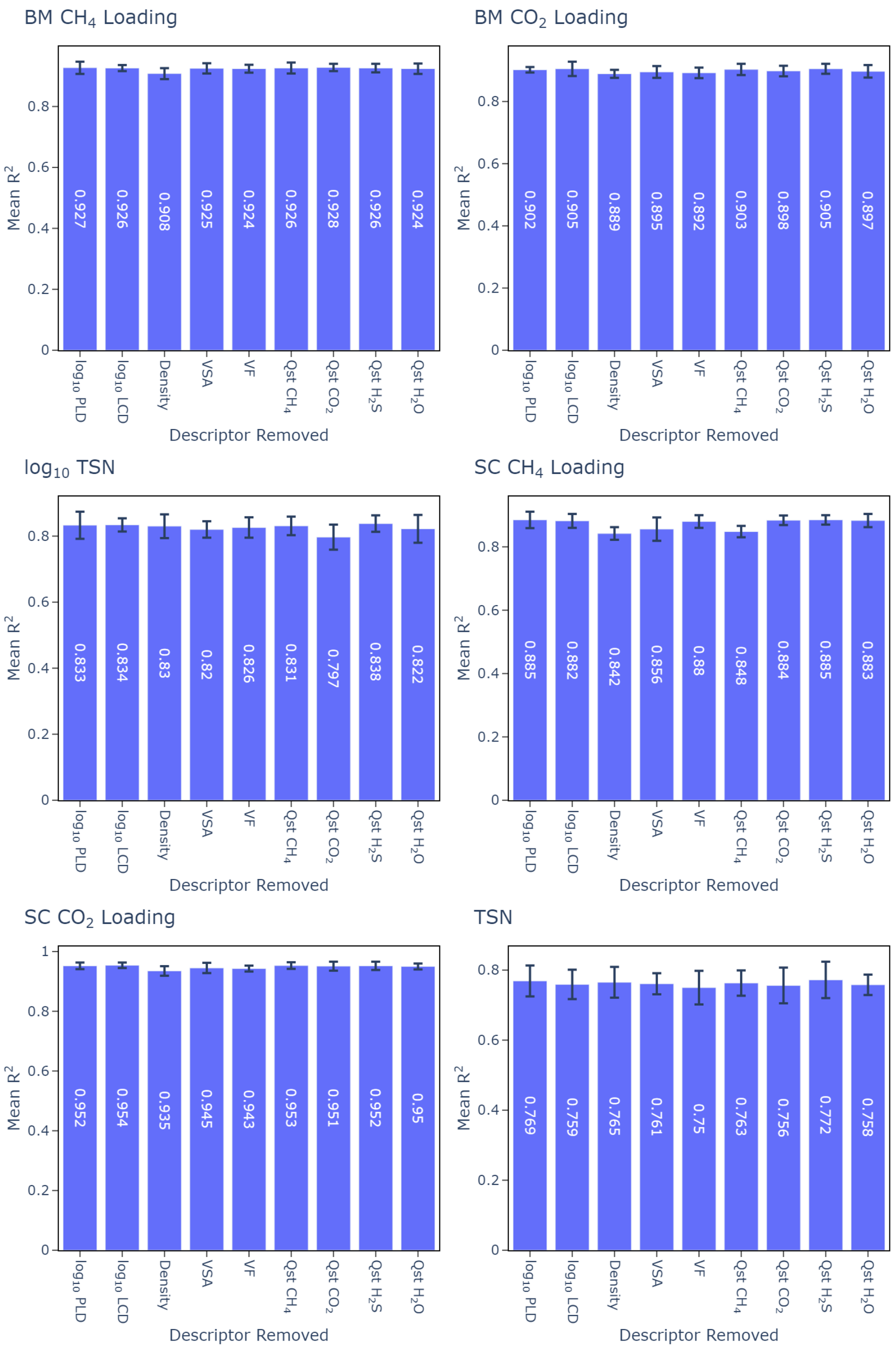

Fig. S31 R^2 of models built using random forest for regression targets, leaving one descriptor in turn

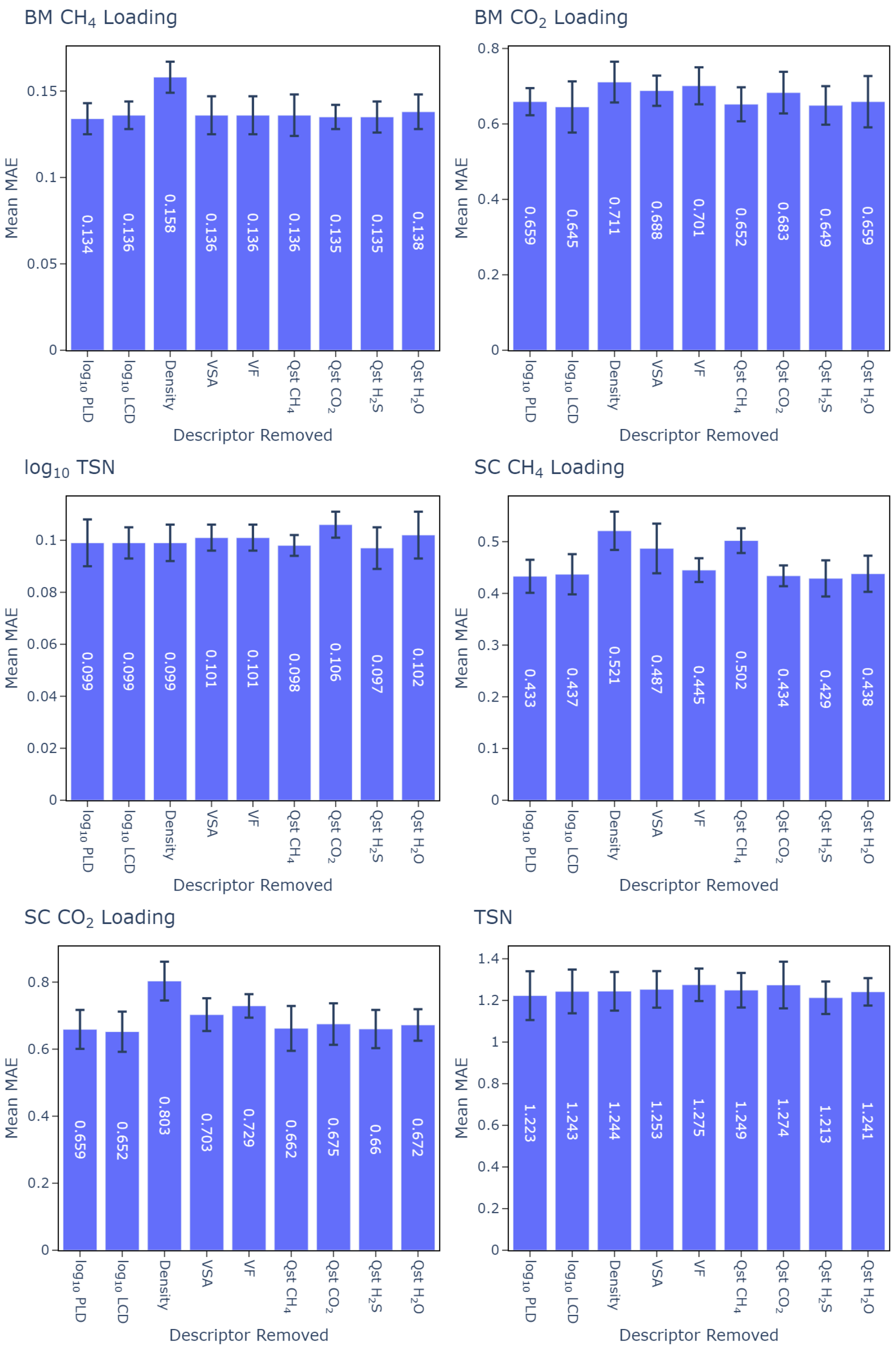

Fig. S32 MAE of models built using random forest for regression targets, leaving one descriptor in turn

Table S8 Analysis of unseen test set descriptors and targets

№	Descriptor/Target	Minimum	Maximum	Mean	Median	Std Dev
1	PLD log10	0.58	1.39	0.84	0.81	0.17
2	LCD log10	0.66	1.49	0.96	0.94	0.16
3	Density (g/cc)	0.13	1.74	0.72	0.70	0.29
4	VSA (m2/cc)	61.03	5269.02	819.63	679.06	650.29
5	VF	0.48	0.95	0.74	0.74	0.09
6	Qst_CH4	-22.58	-5.20	-13.00	-12.58	3.57
7	Qst_CO2	-47.44	-7.26	-20.96	-20.13	5.85
8	Qst_H2S	-41.20	-9.02	-21.55	-20.29	5.71
9	Qst_H2O	-105.37	-5.46	-39.59	-49.43	23.14
10	BM CO2 loading (mol/kg)	2.08	15.30	6.96	6.63	2.45
11	BM CH4 loading (mol/kg)	0.20	3.40	1.67	1.70	0.67
12	SC CO2 loading (mol/kg)	3.21	21.94	11.66	11.38	3.79
13	SC CH4 loading (mol/kg)	1.76	9.22	4.44	4.24	1.31
14	TSN	0.39	15.80	4.74	4.09	2.96
15	LOG10 TSN	-0.41	1.20	0.59	0.61	0.29

5.5 Unseen Test Set

The unseen test set descriptors and targets were analysed by the same statistics as the training set, as shown in Table S8. The ranges distributions of the descriptors and targets are shown in Figures S33 to S37.

5.5.1 Test Set Analysis

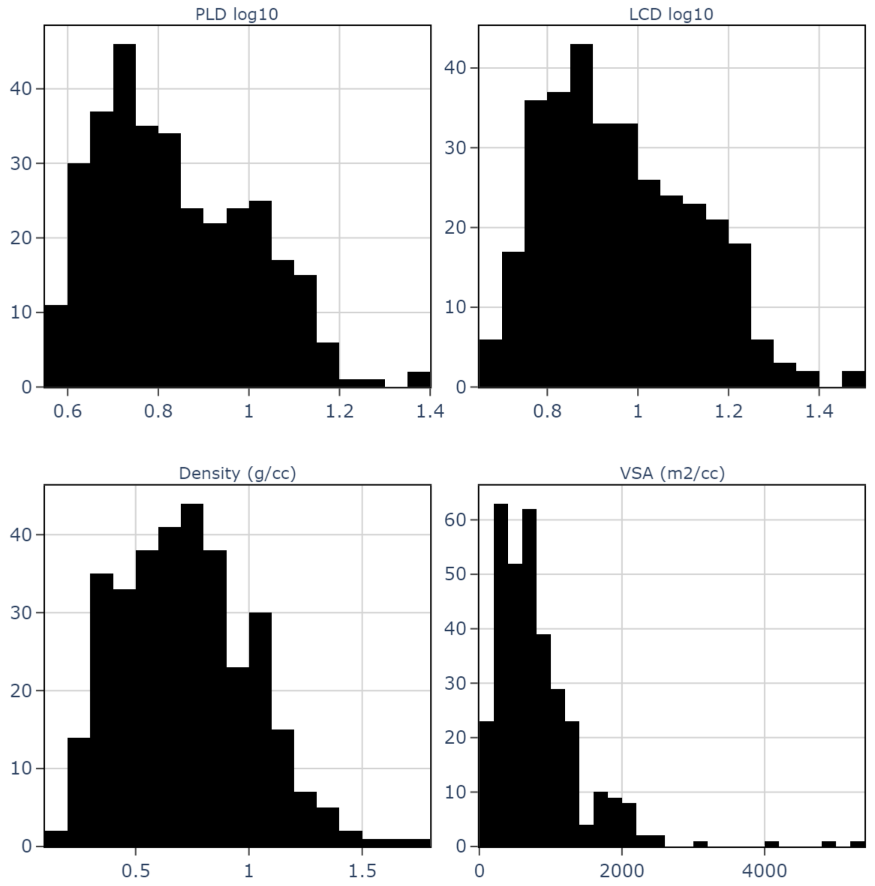

Fig. S33 Histogram of unseen test set descriptor ranges 1-4

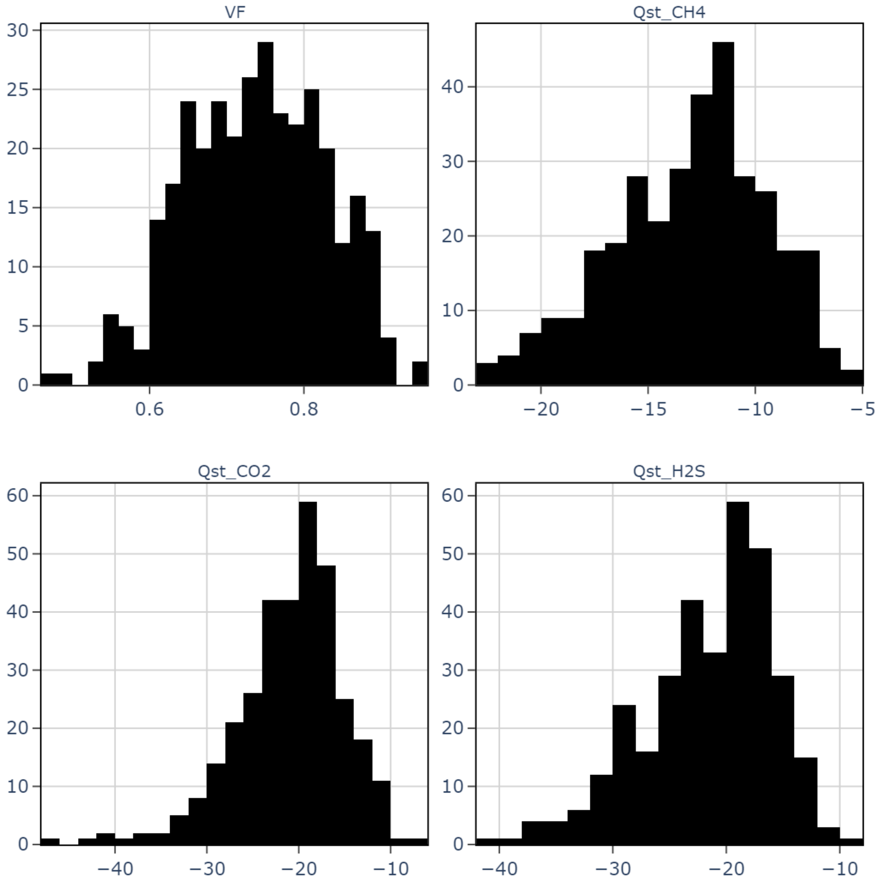

Fig. S34 Histogram of unseen test set descriptor ranges 5-8

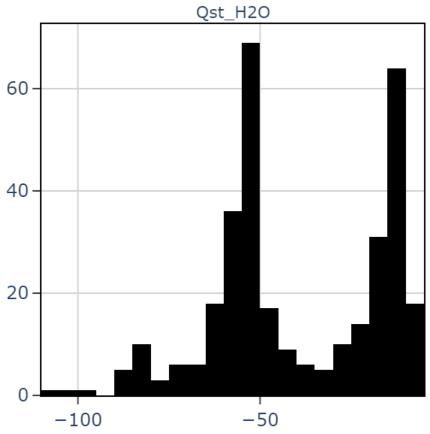

Fig. S35 Histogram of unseen test set descriptor range 9

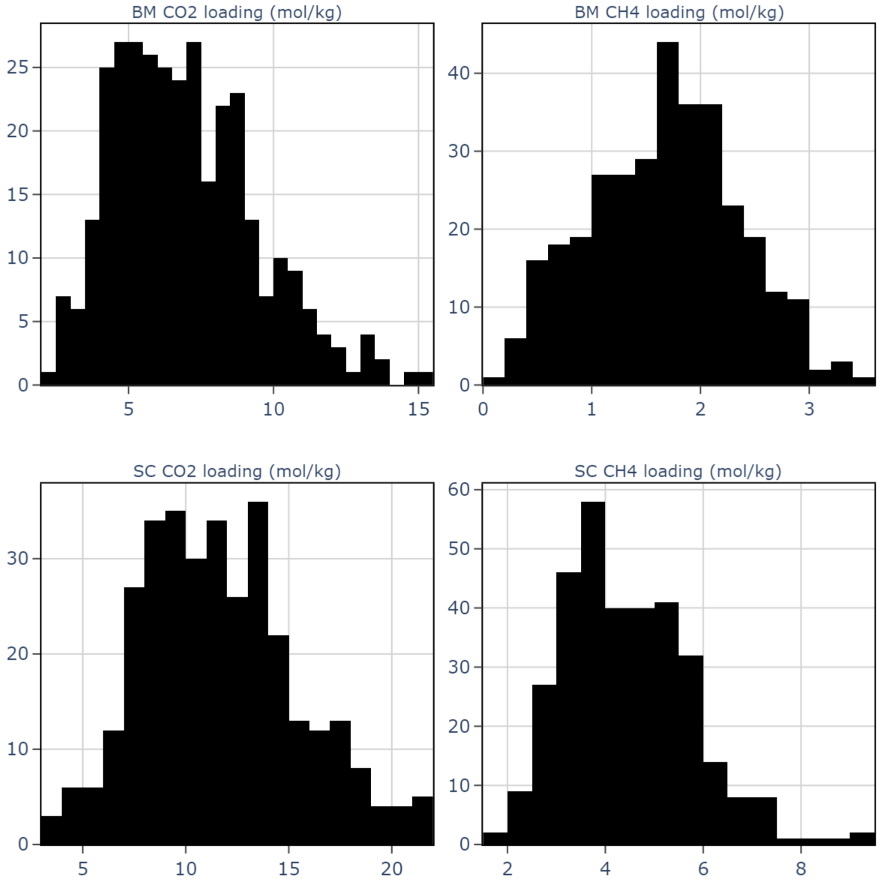

Fig. S36 Histogram of unseen test set target ranges 10-13

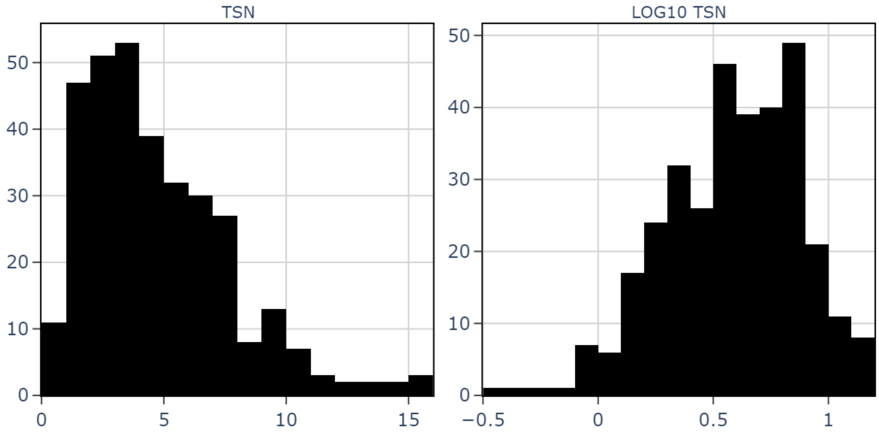

Fig. S37 Histogram of unseen test set target ranges 14-15

The correlation of the unseen test set descriptors can be seen in Figure S38. Correlation between $log_{10}(PLD)$ and $log_{10}(LCD)$ was greater than the training set.

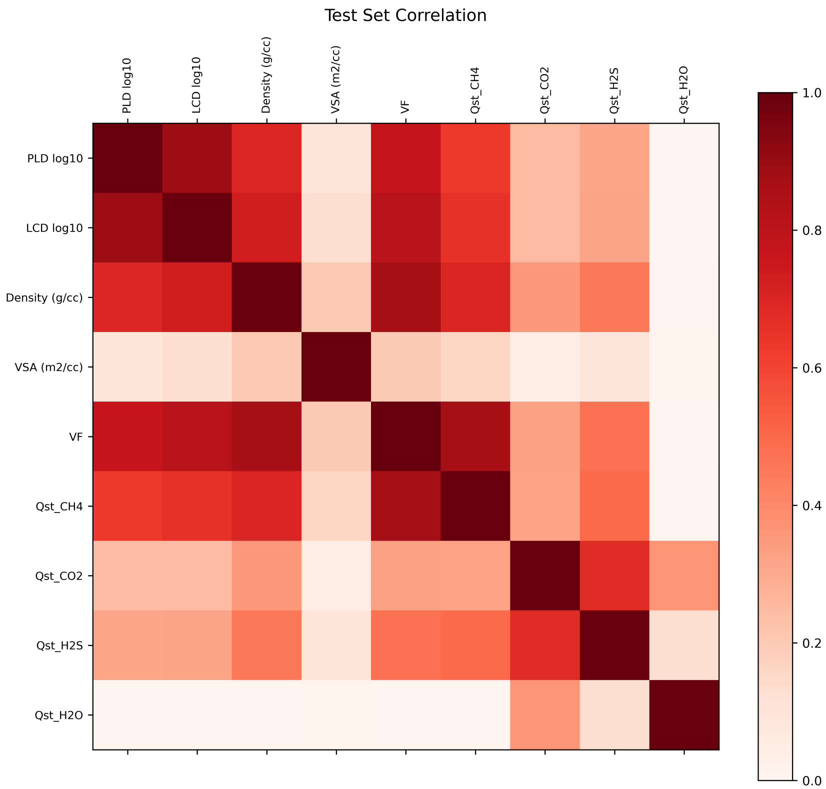

Fig. S38 Correlation of unseen test set descriptors

5.5.2 Regression Results

Models were trained with the full training dataset and tested on the unseen test set. The full prediction and error plots can be seen in Figures S39 to S44.

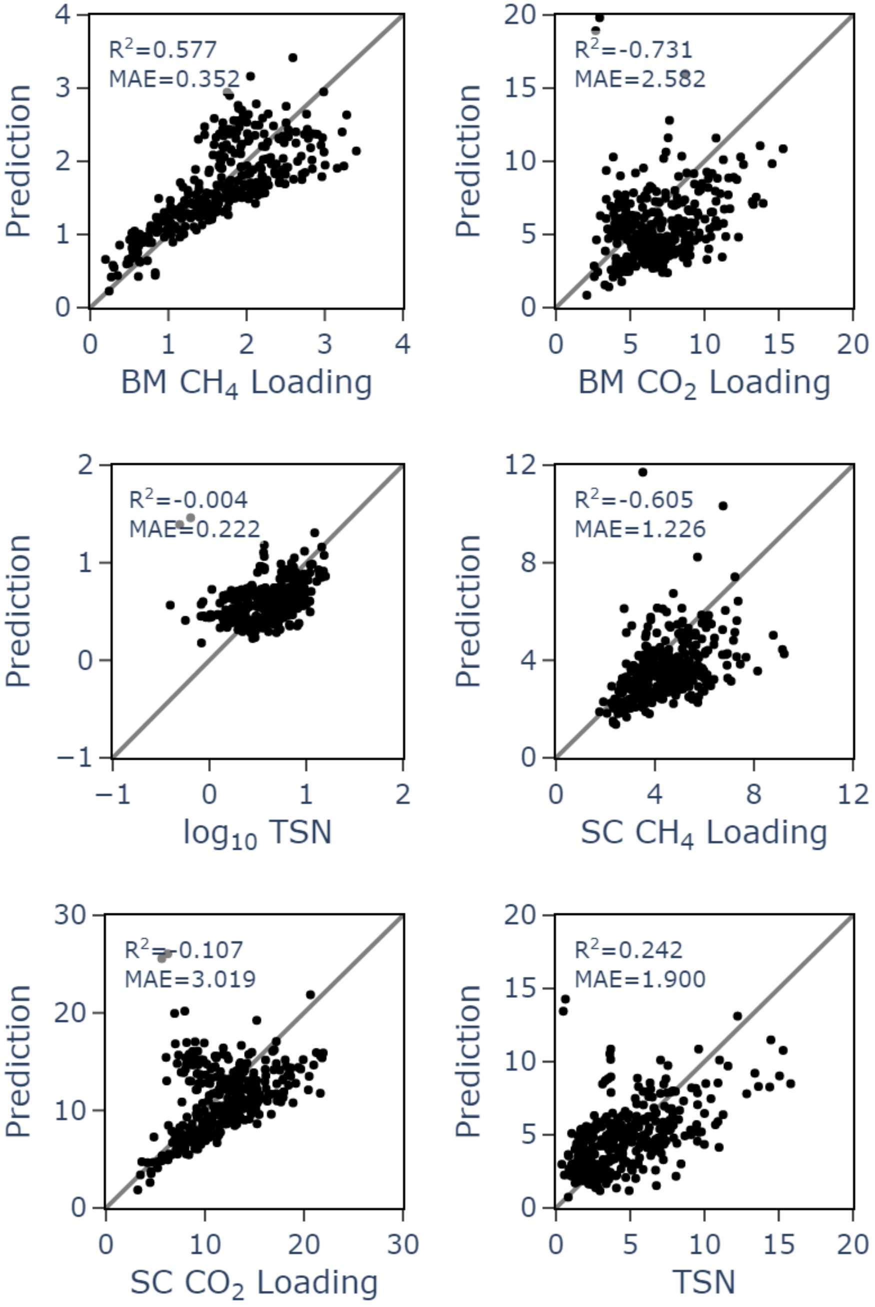

Fig. S39 Full unseen test set regression predictions for MLR

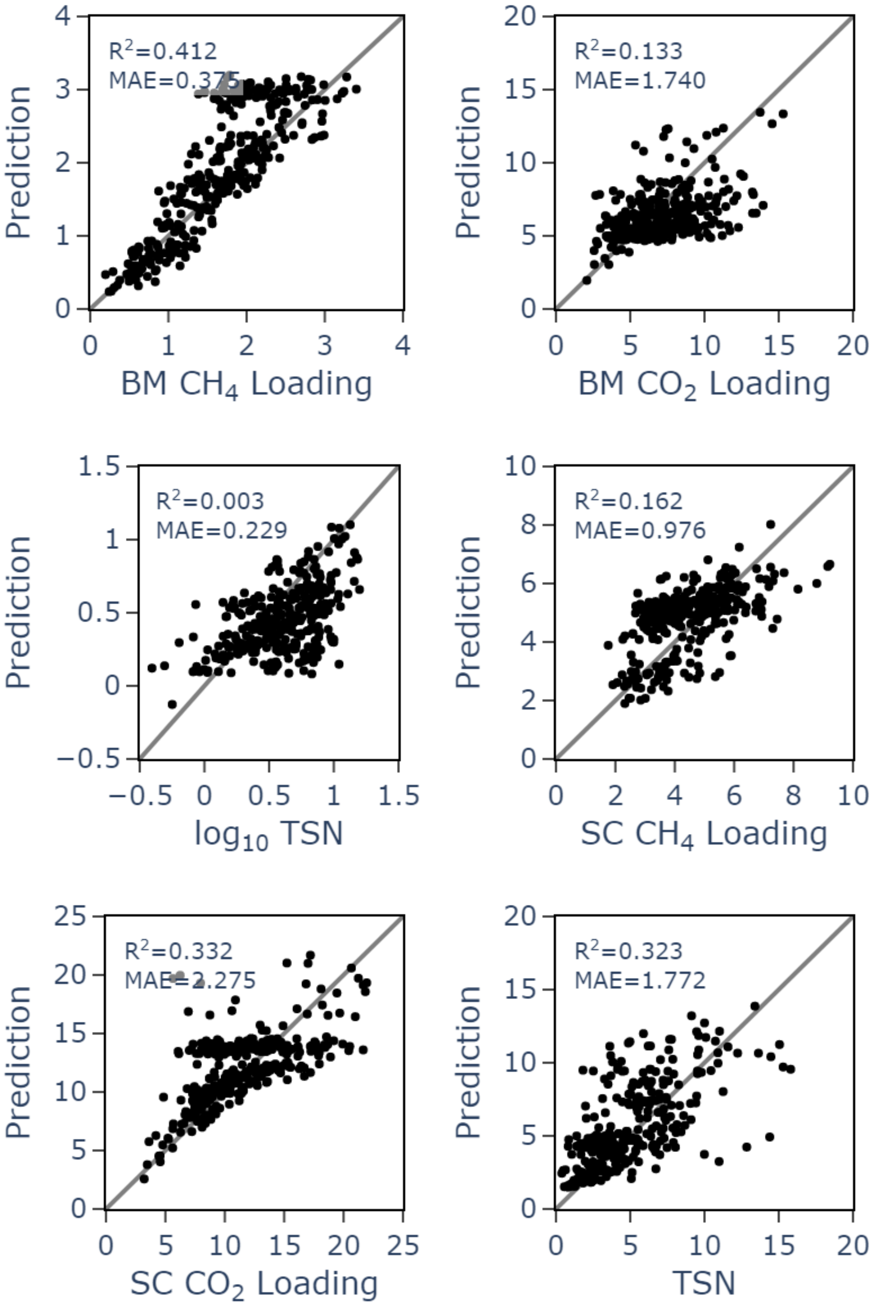

Fig. S40 Full unseen test set regression predictions for RF

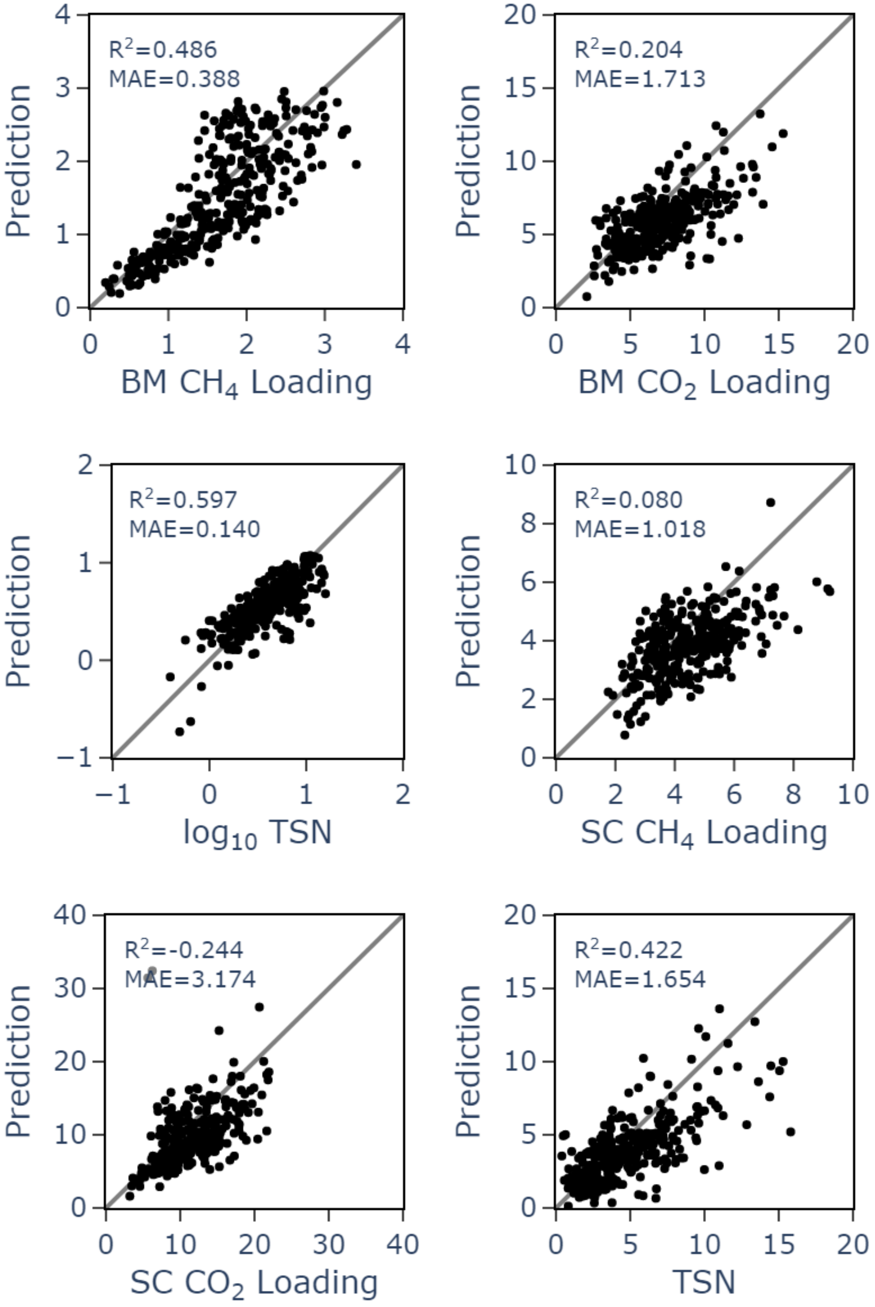

Fig. S41 Full unseen test set regression predictions for SVM

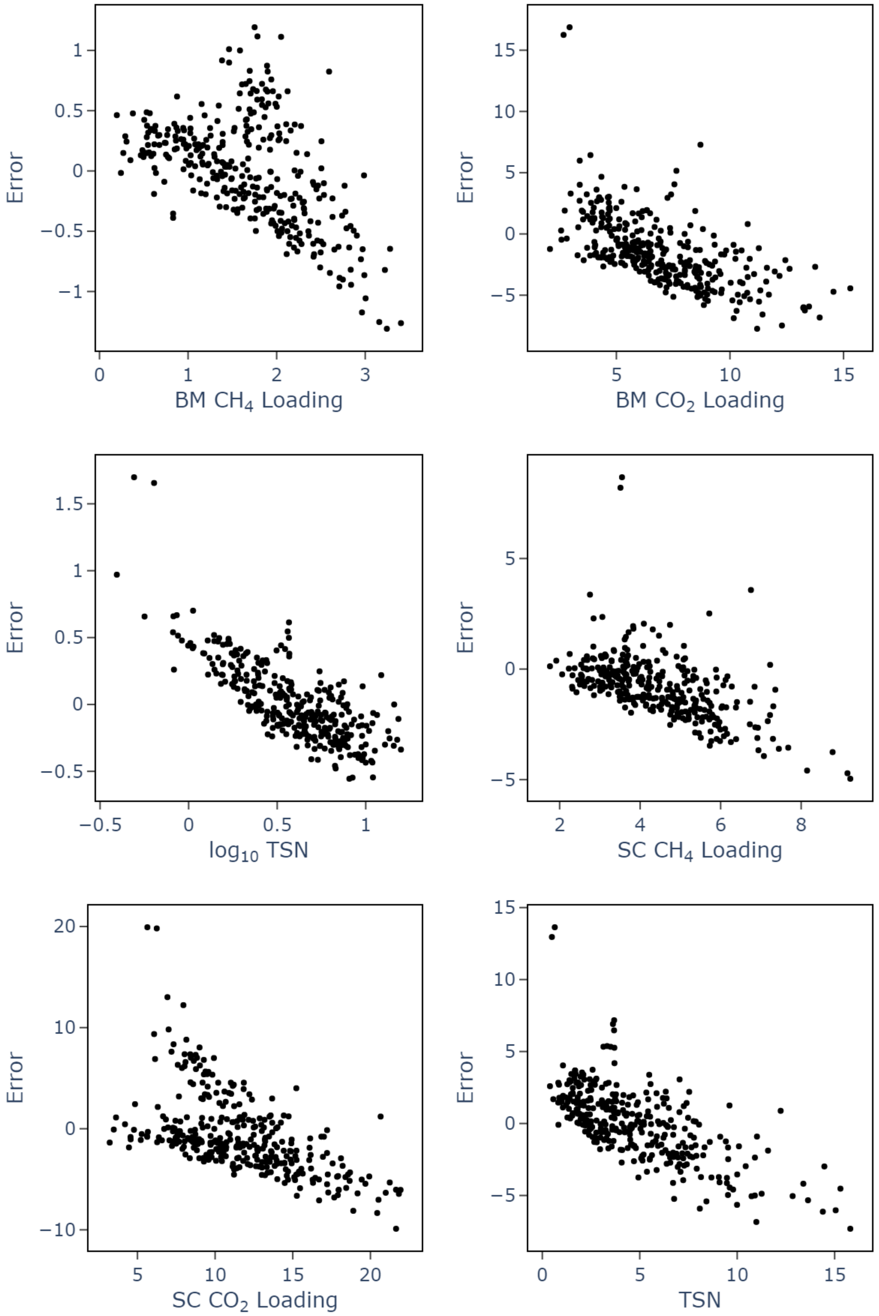

Fig. S42 Full unseen test set error against target range for MLR

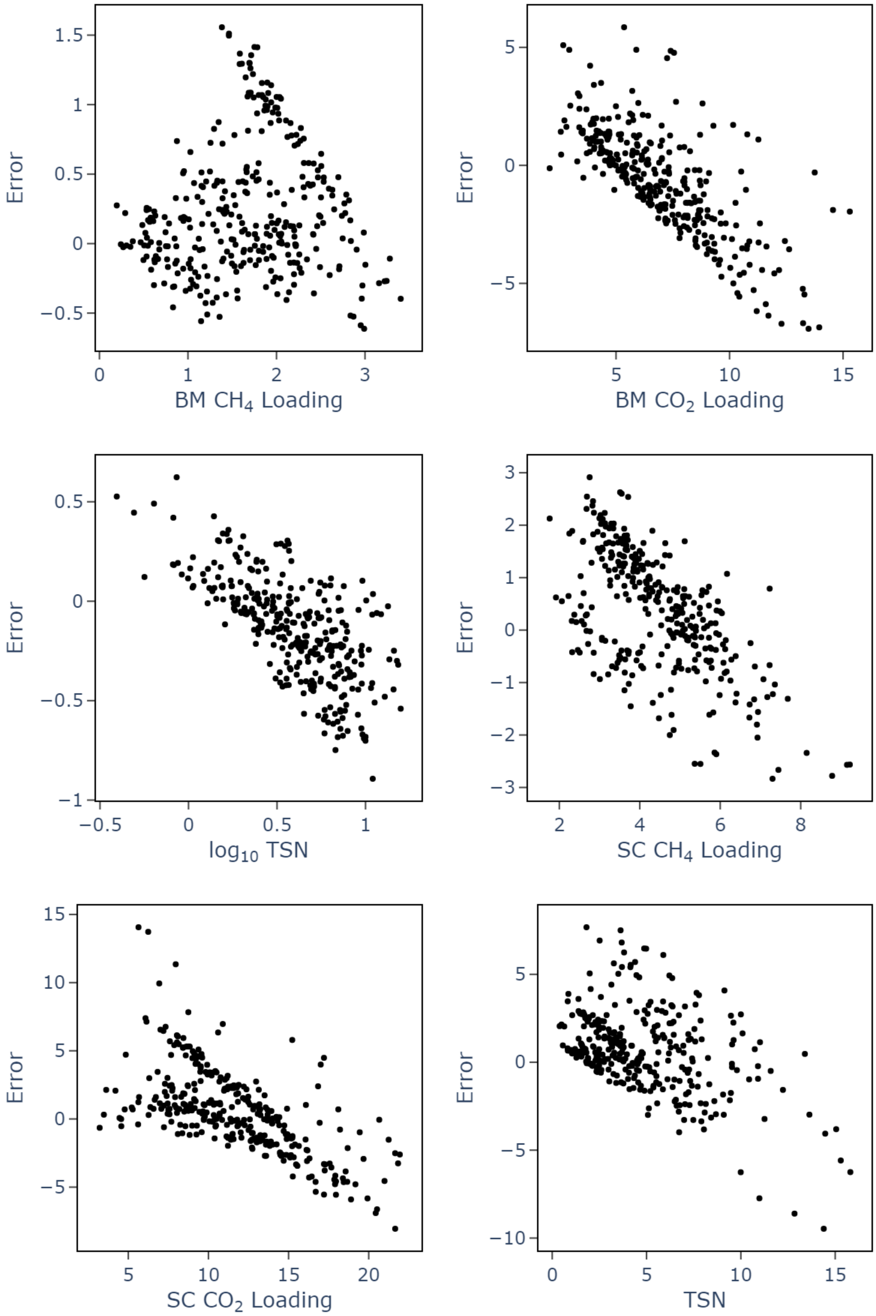

Fig. S43 Full unseen test set error against target range for RF

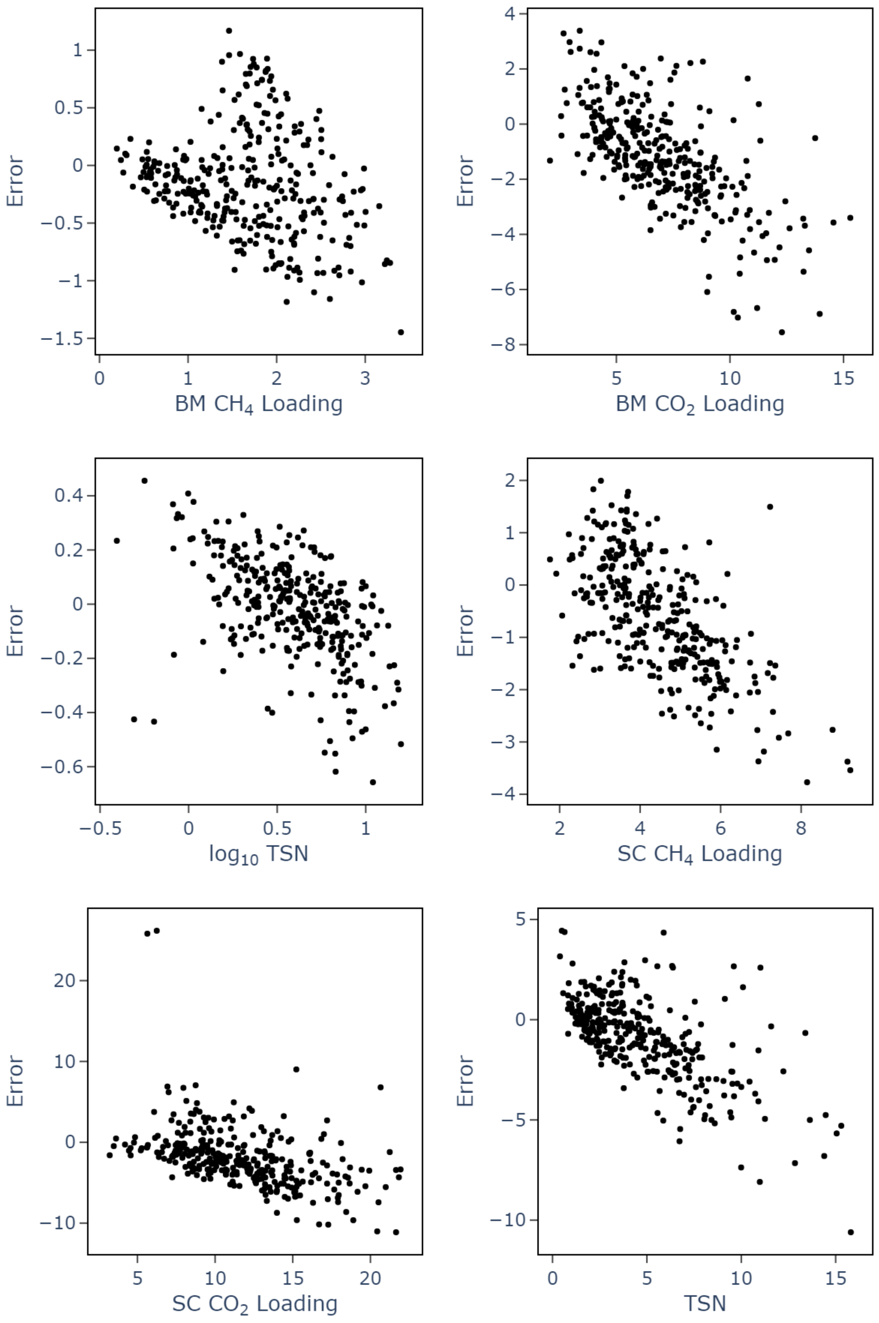

Fig. S44 Full unseen test set error against target range for SVM

5.5.3 Classification Results

The classification models were retrained using the full training set and tested on the unseen test set. The analysis for the cross validation was repeated and can be seen in Figures S45 and S46.

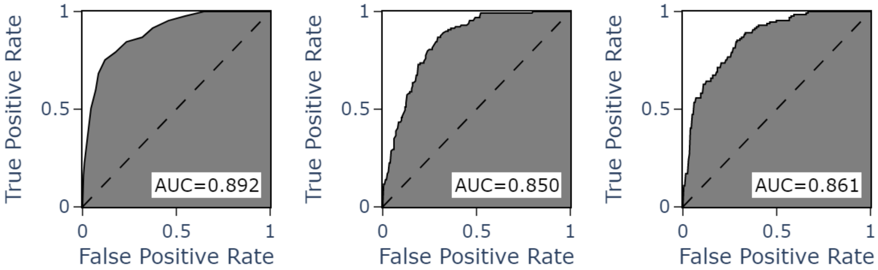

Fig. S45 Receiver operating characteristic curves for unseen test set: KNN (left); RF (middle); SVM (right)

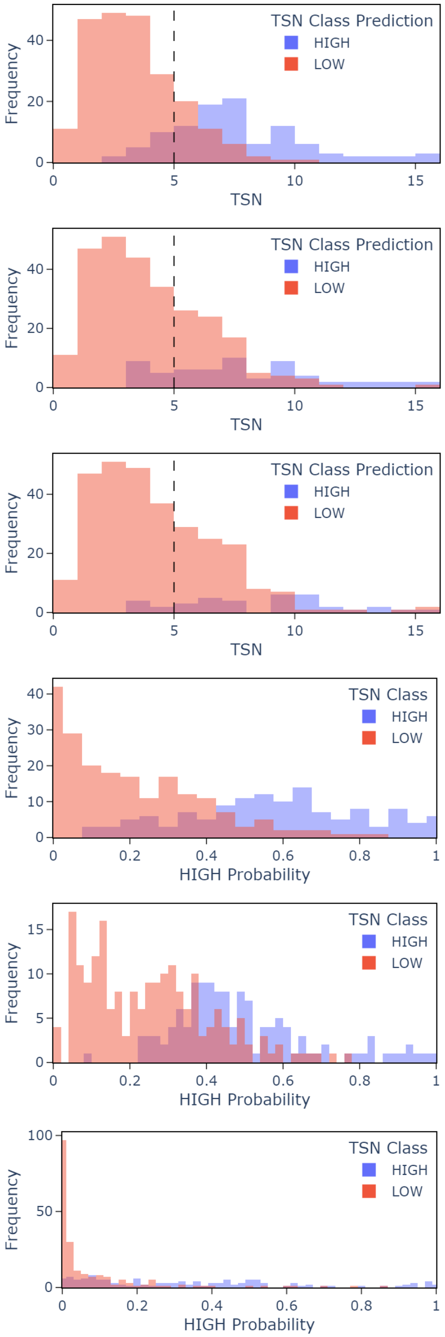

Fig. S46 Top half: Histogram of the TSN values of each predicted class for unseen test set: KNN (top); RF (middle); SVM (bottom). Bottom half: Histogram of HIGH class probability for each class: KNN (top); RF (middle); SVM (bottom).

5.5.4 Test Set Metrics in Full

The metrics for test set predictions are shown in Table S9.

Table S9 Metrics for test set predictions. MAE is in mol kg^{-1}, except for LOG10 TSN, for which MAE is in $\log_{10}$(mol kg^{-1}) units.

	Regression			
No	Target	Method	MAE	R^2
1	TSN	MLR	1.900	0.242
2	LOG10 TSN	MLR	0.222	-0.004
3	SC CO_2 loading	MLR	3.019	-0.107
4	BM CO_2 loading	MLR	2.582	-0.731
5	SC CH_4 loading	MLR	1.226	-0.605
6	BM CH_4 loading	MLR	0.352	0.577
7	TSN	SVM	1.654	0.422
8	LOG10 TSN	SVM	0.140	0.597
9	SC CO_2 loading	SVM	3.174	-0.244
10	BM CO_2 loading	SVM	1.713	0.204
11	SC CH_4 loading	SVM	1.018	0.080
12	BM CH_4 loading	SVM	0.388	0.486
13	TSN	RF	1.772	0.323
14	LOG10 TSN	RF	0.229	0.003
15	SC CO_2 loading	RF	2.275	0.332
16	BM CO_2 loading	RF	1.740	0.133
17	SC CH_4 loading	RF	0.976	0.162
18	BM CH_4 loading	RF	0.375	0.412
	Classification			
	Metric	Method	HIGH	LOW
19	Precision	kNN	0.838	0.818
20	Recall	kNN	0.682	0.915
21	F1 Score	kNN	0.752	0.864
			Value	
22	Accuracy	kNN	0.824	
23	Briers	kNN	0.136	
24	AUC	kNN	0.892	
	Metric	Method	HIGH	LOW
25	Precision	SVM	0.838	0.666
26	Recall	SVM	0.240	0.970
27	F1 Score	SVM	0.373	0.789
			Value	
28	Accuracy	SVM	0.685	
29	Briers	SVM	0.215	
30	AUC	SVM	0.861	
	Metric	Method	HIGH	LOW
31	Precision	RF	0.774	0.698
32	Recall	RF	0.372	0.930
33	F1 Score	RF	0.503	0.797
			Value	
34	Accuracy	RF	0.712	
35	Briers	RF	0.168	
36	AUC	RF	0.85	

Table S10 The six MOFs with the highest absolute prediction error and the six MOFs with the lowest absolute prediction error from the RF regression model for SC CO_2 loading, along with their calculated and predicted loading values, pore limiting diamter (PLD) and void fraction (VF).

	Refcode	Calculated Loading /mol kg^{-1}	Predicted Loading /mol kg^{-1}	Error /mol kg^{-1}	PLD /Å	VF
Large Error	LAWGEW	7.09	16.13	+9.05	6.78	0.74
	OWITIY	13.89	21.18	+7.29	5.80	0.77
	HAYZAL	17.82	11.66	−6.15	18.55	0.78
	TUWVEO	12.02	17.96	+5.94	9.32	0.83
	GITVIP01	5.17	10.41	+5.23	5.47	0.63
	JUTCIM	8.82	14.05	+5.23	14.88	0.74
Small Error	SISPUH	5.33	5.33	0.00	5.22	0.52
	EQOXEN	7.29	7.29	0.00	8.06	0.57
	IJASOC	10.96	10.96	0.00	5.88	0.65
	VOKCOP	3.37	3.37	0.00	4.04	0.47
	BECTAH	5.05	5.05	0.00	5.74	0.51
	NERMON	3.81	3.81	0.00	4.70	0.44

Supplementary Note 6
Analysis of Outliers

In addition to analysis on a statistical scale, the performance of models of both kinds was assessed on an individual-MOF level by analysis of outlying MOFs. The random forest model for SC CO_2 loading is taken as an example of the best-performing regression model. Calculated and predicted SC CO_2 loading of the six MOFs with the highest absolute prediction error are shown in Table S10, along with relevant structural features, VF, which showed the highest importance in the model, and PLD. To ensure any uniting factors observed among the six were not simply artifacts of the dataset as a whole, the six structures with the lowest prediction errors are also given in Table S10. The majority of the largest errors correspond to an overprediction of loading rather than an underprediction, and are associated with large-pored structures displaying higher void fraction, while in general the best-predicted structures display lower void fraction and smaller PLD than the poorly-predicted MOFs. The prevalence of high void fractions among the poorly-predicted structures likely relates to the peak in the relationship between void fraction and calculated SC CO_2 loading seen in the main text. The dataset as a whole contains relatively few structures with VF larger than that of the peak, so the model may have received insufficient training data in this region to effectively learn the dip in performance when VF is very high, leading to overprediction.

Outliers of the classification model are also identified. They include both structures with high TSN which were incorrectly classified as low, and structures with low TSN which were incorrectly classified as high. Details of the six worst-classified structures of each category are given in Table S11, with the worst-classified structures defined as those which are incorrectly classified and whose calculated TSN is furthest from the threshold of 5 mol kg^{-1} (highest

value for those incorrectly classified as low and lowest value for those incorrectly classified as high). Again, the best-classified MOFs are also included in the table for comparison, with the best-classified MOFs defined as those which are correctly classified with the highest confidence (highest probability for high-performing MOFs and lowest probability for low-performing MOFs). For the training and validation data several MOFs are classified with a probability of 1 or 0, so to obtain a range of well-classified MOFs, two sets of correctly classified high-performing MOFs are listed. These are the 6 correctly classified as high-performing with a probability of 1 and the highest GCMC TSN and the six correctly classified as high performing with a probability of 1 and the lowest GCMC TSN. A similar two sets of correctly classified low-performing MOFs are listed.

Several observations can be made relating to Table S11. For one thing, of the 6 selected incorrectly classified MOFs with particularly high GCMC-calculated performance, most are classified with probabilities fairly close to 0.5. This highlights the positive fact that the model is not confidently wrong about values of TSN in this extreme. Some of the 6 incorrectly classified MOFs with particularly low GCMC-calculated performance were also classified with probabilities close to 0.5, although in this category there were also examples of higher probabilities being assigned. In a similar vein, the very confidently classified MOFs do not include either low- or high-performers whose TSN values are very close to 5, showing that borderline MOFs are not classified with maximum confidence. Among the high-performing MOFs classified with a probability of 1 is QUDJEF, a structure that was identified as among the highest performers across the whole dataset. It was confirmed that the remaining top performers identified by the GCMC screening were also correctly classified by the model, all with high-performing probability above 0.9 with the exception of YOCSEQ (probability=0.63).

In terms of structural features among the well-classified and poorly-classified MOFs, PLD and VF vary significantly among incorrectly-classified MOFs of both kinds. High-performing MOFs which were well classified all have VF within a similar range, close to 0.6, and well-classified low-performing MOFs have smaller VFs in the range 0.4-0.55. The poorly classified MOFs do not have VFs which fit as neatly into this pattern. In particular, low-performing MOFs which were incorrectly classified as high-performing tend to have larger VF than well-classified low-performing MOFs. A similar effect appears to be seen here for the relationship between VF and TSN as was seen in the main text for the relationship between VF and CO_2 uptake.

Table S11 Details of the MOFs which are worst-classified and best-classified b ythe RF TSN model. The worst-classified MOFs are defined as those which a reincorrectly classified and have TSN furthest from the threshold. The best-classifi edMOFs are defined as those which are correctly classified with the highest confiden ce.Since several correctly classified MOFs have a probability of 0 or 1, they are selec tedfirst in descending order and then in ascending order of TSN. Details given are:Refcode, TSN, High-performing probability, pore limiting diameter (PLD) and voidfraction (VF).

	Refcode	TSN, mol kg^{-1}	Probability	PLD, Å	VF
High TSN, incorrectly classified	KEFZEC	12.87	0.302	4.06	0.48
	TARVOX	12.51	0.406	4.37	0.41
	TARVUD	11.17	0.426	5.05	0.43
	LASWIN	10.60	0.496	3.83	0.51
	WITLUI	8.76	0.416	4.54	0.51
	FAQLER	8.50	0.484	7.04	0.60
High TSN, correctly classified, maximise TSN	QUDJEF	30.67	1	4.27	0.70
	QISNAJ	21.25	1	5.40	0.73
	PASMUT	18.87	1	3.99	0.57
	GONNAA	18.25	1	5.82	0.69
	SIKYOB	18.06	1	4.44	0.69
	OHAHIP	17.44	1	4.90	0.65
High TSN, correctly classified, minimise TSN	YEBGOD	7.27	1	4.90	0.62
	WORKUL	7.58	1	5.65	0.67
	CEDFIC	7.81	1	4.94	0.62
	CAGSAG	8.47	1	5.29	0.66
	XUDYEC	8.52	1	5.05	0.64
	XUMVAD	8.70	1	5.10	0.62
Low TSN, incorrectly classified	OWITIY	1.96	0.614	5.80	0.77
	DITNUR	2.32	0.552	3.98	0.43
	IPUPIU	2.46	0.918	8.73	0.75
	PURQOJ	2.57	0.676	6.85	0.69
	GITVIP01	2.95	0.764	5.47	0.63
	EQOCOC01	2.73	0.946	5.48	0.72
Low TSN, correctly classified, minimise TSN	COKQIE	0.15	0	4.51	0.37
	VIXBEK	0.26	0	4.08	0.44
	SARHEA	0.27	0	4.35	0.41
	NENVAE	0.37	0	4.66	0.39
	RAXYOF	0.43	0	4.32	0.44
	TAQGOK	0.62	0	4.66	0.39
Low TSN, correctly classified, maximise TSN	TEQTEQ	3.51	0	5.80	0.52
	ADODII	3.30	0	3.52	0.54
	IVITOY	3.29	0	4.17	0.46
	LAGDAB	3.23	0	6.01	0.51
	PEKTOQ	3.14	0	4.18	0.48
	LENROM	3.11	0	4.28	0.44

Supplementary Note 7 Predictions from the External Test Set.

Table S12 details the six MOFs predicted to have the highest total CO_2 uptake among the external test set according to the RF regression models trained in this work. It includes predicted and calculated loading values, as

Table S12 Details of the six MOFs predicted to display the highest single component CO_2 loading among the external test set by the random forest regression model: the identifier, predicted loading, calculated (GCMC) loading, pore limiting diameter, void fraction, topology and degree of interpenetration (DI).

Numerical Identifier	Predicted SC CO_2 Loading /mol kg^{-1}	GCMC SC CO_2 Loading /mol kg^{-1}	PLD /Å	VF	Topology	DI
2512	21.7	17.2	7.04	0.80	pcu	2
2000673	21.0	15.2	9.86	0.81	tbo	1
5027410	21.0	17.0	6.96	0.80	pcu	2
5083419	20.6	20.7	11.5	0.82	pcu	2
3000121	20.0	6.24	10.46	0.87	dia	2
3886	19.7	21.2	6.69	0.79	pcu	2

well as PLD and VF, and topology and degree of interpenetration as determined by the CrystalNets.jl software. [24] A degree of interpenetration of one indicates a structure composed of only one net, i.e. a structure which is not interpenetrated. Figure S47 gives visualisations of their structures.

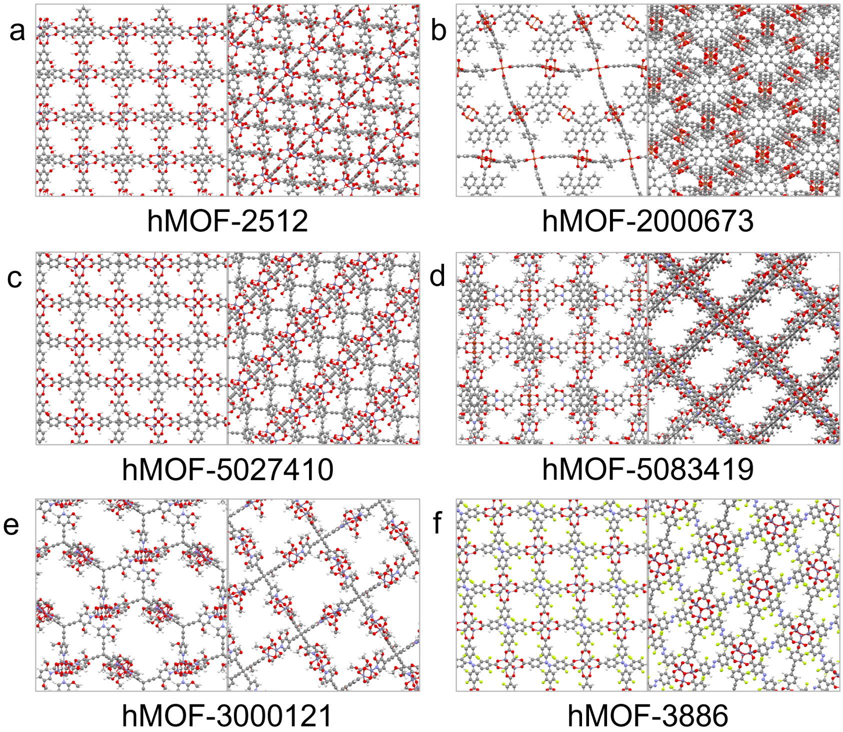

Fig. S47 Visualisations of the six MOFs predicted to have the highest SC CO_2 loading among the external test set by the RF regression model. The MOFs are a) hMOF-2512, metal is Zn, b) hMOF-2000673 metal is Cu c) hMOF-5027410, metal is Zn d) hMOF-5083419, metal is Cu f) hMOF-3000121, metal is Zn g) hMOF-3886, metal is Zn.

Supplementary References

[1] Glover, J., Besley, E.: A High-Throughput Screening of Metal-Organic Framework Based Membranes for Biogas Upgrading. Faraday Discuss. **231**, 235–257 (2021). https://doi.org/10.1039/D1FD00005E

[2] Altintas, C., Avci, G., Daglar, H., Nemati Vesali Azar, A., Erucar, I., Velioglu, S., Keskin, S.: An Extensive Comparative Analysis of Two MOF Databases: High-Throughput Screening of Computation-Ready MOFs for CH_4 and H_2 Adsorption. J. Mater. Chem. A **7**(16), 9593–9608 (2019). https://doi.org/10.1039/C9TA01378D

[3] Chen, T., Manz, T.A.: Identifying Misbonded Atoms in the 2019 CoRE Metal-Organic Framework Database. RSC Adv. **10**, 26944–26951 (2020). https://doi.org/10.1039/D0RA02498H

[4] Burner, J., Luo, J., White, A., Mirmiran, A., Kwon, O., Boyd, P.G., Maley, S., Gibaldi, M., Simrod, S., Ogden, V., Woo, T.K.: ARCMOF: A Diverse Database of Metal-Organic Frameworks with DFT-Derived Partial Atomic Charges and Descriptors for Machine Learning. Chem. Mater **35**, 900–916 (2023). https://doi.org/10.1021/acs.chemmater.2c02485

[5] Willems, T.F., Rycroft, C.H., Kazi, M., Meza, J.C., Haranczyk, M.: Algorithms and Tools for High-Throughput Geometry-Based Analysis of Crystalline Porous Materials. Micropor. Mesopor. Mater. **149**, 134–141 (2012). https://doi.org/10.1016/j.micromeso.2011.08.020

[6] Jablonka, K.M., Rosen, A.S., Krishnapriyan, A.S., Smit, B.: An Ecosystem for Digital Reticular Chemistry. ACS Cent. Sci. **9**(4), 563–581 (2023)

[7] Ongari, D., Talirz, L., Jablonka, K.M., Siderius, D.W., Smit, B.: Data-Driven Matching of Experimental Crystal Structures and Gas Adsorption Isotherms of Metal-Organic Frameworks. J. Chem. Eng. Data **67**(7), 1743–1756 (2022)

[8] Ong, S.P., Richards, W.D., Jain, A., Hautier, G., Kocher, M., Cholia, S., Gunter, D., Chevrier, V.L., Persson, K.A., Ceder, G.: Python Materials Genomics (pymatgen): A Robust, Open-Source Python Library for Materials Analysis. Comp. Mater. Sci. **68**, 314–319 (2013)

[9] Hagberg, A.A., Schult, D.A., Swart, P.J.: Exploring network structure, dynamics, and function using networkx. In: Varoquaux, G., Vaught, T., Millman, J. (eds.) Proceedings of the 7th Python in Science Conference, Pasadena, CA USA, pp. 11–15 (2008)

[10] Velioglu, S., Keskin, S.: Revealing the effect of structure curations on the

simulated co_2 separation performances of mofs. Mater. Adv. **1**, 341–353 (2020). https://doi.org/10.1039/D0MA00039F

[11] Moghadam, P.Z., Li, A., Wiggin, S.B., Tao, A., Maloney, A.G.P., Wood, P.A., Ward, S.C., Fairen-Jimenez, D.: Development of a Cambridge Structural Database Subset: A Collection of Metal-Organic Frameworks for Past, Present, and Future. Chem. Mater. **29**, 2618–2625 (2017). https://doi.org/10.1021/acs.chemmater.7b00441

[12] Wilmer, C.E., Leaf, M., Lee, C.Y., Farha, O.K., Hauser, B.G., Hupp, J.T., Snurr, R.Q.: Large-Scale Screening of Hypothetical Metal–Organic Frameworks. Nat. Chem. **4**, 83–89 (2012). https://doi.org/10.1038/nchem.1192

[13] Shah, M.S., Tsapatsis, M., Siepmann, J.I.: Identifying Optimal Zeolitic Sorbents for Sweetening of Highly Sour Natural Gas. Angew. Chem. Int. Ed. **55**, 5938–5942 (2016). https://doi.org/10.1002/anie.201600612

[14] Qiao, Z., Xu, Q., Jiang, J.: Computational Screening of Hydrophobic Metal–Organic Frameworks for the Separation of H_2S and CO_2 From Natural Gas. J. Mater. Chem. A **6**, 18898–18905 (2018). https://doi.org/10.1039/C8TA04939D

[15] Ghanbari, T., Abnisa, F., Wan Daud, W.M.A.: A Review on Production of Metal Organic Frameworks (MOF) for CO_2 Adsorption. Sci. Total Environ. **707**, 135090 (2020). https://doi.org/10.1016/J.SCITOTENV.2019.135090

[16] Wang, B., Côté, A.P., Furukawa, H., O'keeffe, M., Yaghi, O.M.: Colossal Cages in Zeolitic Imidazolate Frameworks as Selective Carbon Dioxide Reservoirs. Nature Lett. **453**, 207–212 (2008). https://doi.org/10.1038/nature06900

[17] Pan, L., Adams, K.M., Hernandez, H.E., Wang, X., Zheng, C., Hattori, Y., Kaneko, K.: Porous Lanthanide-Organic Frameworks: Synthesis, Characterization, and Unprecedented Gas Adsorption Properties. J. Am. Chem. Soc. **125**(10), 3062–3067 (2003). https://doi.org/10.1021/ja028996w

[18] Zhou, X., Huang, W., Miao, J., Xia, Q., Zhang, Z., Wang, H., Li, Z.: Enhanced Separation Performance of a Novel Composite Material GrO@MIL-101 for CO_2/CH_4 Binary Mixture. Chem. Eng. J. **266**, 339–344 (2015). https://doi.org/10.1016/j.cej.2014.12.021

[19] Aghaji, M.Z., Fernandez, M., Boyd, P.G., Daff, T.D., Woo, T.K.: Quantitative Structure–Property Relationship Models for Recognizing Metal Organic Frameworks (MOFs) with High CO_2 Working Capacity and CO_2/CH_4 Selectivity for Methane Purification. Eur J. Inorg. Chem. **2016**, 4505–4511 (2016). https://doi.org/10.1002/ejic.201600365

[20] Fernandez, M., Boyd, P.G., Daff, T.D., Aghaji, M.Z., Woo, T.K.: Rapid and Accurate Machine Learning Recognition of High Performing Metal Organic Frameworks for CO_2 Capture. J. Phys. Chem. Lett. **5**, 3056–3060 (2014). https://doi.org/10.1021/jz501331m

[21] Cavenati, S., Grande, C.A., Rodrigues, A.E.: Adsorption Equilibrium of Methane, Carbon Dioxide, and Nitrogen on Zeolite 13X at High Pressures. J. Chem. Eng. Data. **49**(4), 1095–1101 (2004). https://doi.org/10.1021/je0498917

[22] Sarker, A.I., Aroonwilas, A., Veawab, A.: Equilibrium and Kinetic Behaviour of CO2 Adsorption onto Zeolites, Carbon Molecular Sieve and Activated Carbons. Energy Procedia **114**, 2450–2459 (2017). https://doi.org/10.1016/J.EGYPRO.2017.03.1394

[23] Dubbeldam, D., Calero, S., Ellis, D.E., Snurr, R.Q.: RASPA: Molecular Simulation Software for Adsorption and Diffusion in Flexible Nanoporous Materials. Mol. Simul. **42**, 81–101 (2016). https://doi.org/10.1080/08927022.2015.1010082

[24] Zoubritzky, L., François-Xavier-Coudert: CrystalNets.jl: Identification of Crystal Topologies. SciPost Chem. **1**, 005 (2022)
